# Circulating α-Klotho and Multidimensional Aging and Frailty Outcomes: A Systematic Review and Meta-Analysis from the European Renal Association CKD-MBD Working Group

**DOI:** 10.1007/s00223-026-01537-3

**Published:** 2026-04-30

**Authors:** Mustafa Guldan, Lasin Ozbek, Derya G. Fidan, Arif E. Narin, Ditte Hansen, Juan M. Diaz-Tocados, Mathias Haarhaus, Martin H. de Borst, Carlo Alfieri, Pietro M. Ferraro, Antonio Bellasi, Mehmet Kanbay

**Affiliations:** 1 School of Medicine, Koç University, Istanbul, Turkey; 2 Department of Nephrology, Copenhagen University Hospital - Herlev, Copenhagen, Denmark; 3 Institute of Clinical Medicine, University of Copenhagen, Copenhagen, Denmark; 4 Vascular and Renal Translational Research Group, Institut de Recerca Biomèdica de Lleida (IRBLleida), Lleida, Spain; 5 Cooperative Research Network Oriented Towards Health Outcomes (RICORS), Carlos III Health Institute (ISCIII), 28029 Madrid, Spain; 6 Division of Renal Medicine, Department of Clinical Science, Intervention and Technology, Karolinska University Hospital, Karolinska Institutet, Stockholm, Sweden; 7 Diaverum Sweden, Malmö, Sweden; 8 Division of Nephrology, Department of Internal Medicine, University Medical Center Groningen, University of Groningen, Groningen, The Netherlands; 9 Nephrology, Dialysis and Renal Transplantation, Fondazione IRCCS Ca’ Granda Ospedale Policlinico Milan, 20122 Milan, Italy; 10 Department of Clinical Sciences and Community Health, University of Milan, 20122 Milan, Italy; 11 Section of Nephrology, Department of Medicine, Università degli Studi di Verona and Nephrology Unit, Azienda Ospedaliera Universitaria Integrata Verona, Verona, Italy; 12 Service of Nephrology, Ospedale Regionale di Lugano, Ospedale Civico, Ente Ospedaliero Cantonale, Lugano, Switzerland; 13 Faculty of Biomedical Sciences, Università della Svizzera italiana, Lugano, Switzerland; 14 Division of Nephrology, Department of Internal Medicine, School of Medicine, Koç University, Rumelifeneri Yolu, 34450 Sarıyer, Istanbul Turkey

**Keywords:** Klotho, Frailty, Physical activity, Exercise training, Body composition, Fracture

## Abstract

Although α-Klotho has gained attention as a promising biomarker of aging, its association with frailty and broader aging-related outcomes beyond chronic kidney disease–mineral and bone disorder remains incompletely characterized. This systematic review and meta-analysis aimed to investigate the association between circulating α-Klotho levels and aging-related outcomes, including frailty, effects of physical activity and exercise interventions, body composition, cognitive and neuropsychiatric status, sarcopenia, and bone mineral density (BMD). A comprehensive literature search was performed across multiple electronic databases, including Ovid MEDLINE, Web of Science, Scopus, PubMed, and the Cochrane Library, to identify relevant studies published up to April 30, 2025. Pooled analyses were conducted using random-effects models, with effect estimates synthesized as mean differences, odds ratios, or correlation coefficients, and heterogeneity assessed using the I^2^ statistic. Risk of bias was assessed using design-specific tools, including the Newcastle–Ottawa Scale, the Risk of Bias 2 tool (RoB2) of the Cochrane collaboration, and the Joanna Briggs Institute (JBI) checklists. A total of 109 studies met the inclusion criteria and were included. In our meta-analysis, higher circulating α-Klotho levels were significantly associated with lower odds of frailty (OR = 0.61, 95% CI: 0.49, 0.77; *p* < 0.0001, I^2^ = 0%). Exercise interventions increased circulating α-Klotho (95% CI: 93.93, 261.73, *p* < 0.0001; I^2^ = 89%), but habitual physical activity showed no significant effect on α-Klotho compared to inactive controls (*p* = 0.25; I^2^ = 100%). Lower circulating α-Klotho levels were observed in individuals with osteoporosis compared with those with normal BMD (95% CI: − 114.98, − 25.49; *p* = 0.002) and in individuals with osteopenia compared with those with normal BMD (95% CI: − 123.22, − 0.74; *p* = 0.05), and were associated with fractures. Based on qualitative synthesis, circulating α-Klotho levels were positively associated with multiple physical function outcomes, including grip strength, short physical performance battery scores, and sit-to-stand performance, with mixed findings for gait speed and the 6-min walk test. Circulating α-Klotho appears to be a promising biomarker for frailty, physical function, and bone health in aging; however, evidence for cognitive outcomes remains limited and inconsistent. Substantial heterogeneity and the observational nature of most studies highlight the need for more standardized and longitudinal research.

**Graphical abstract:**

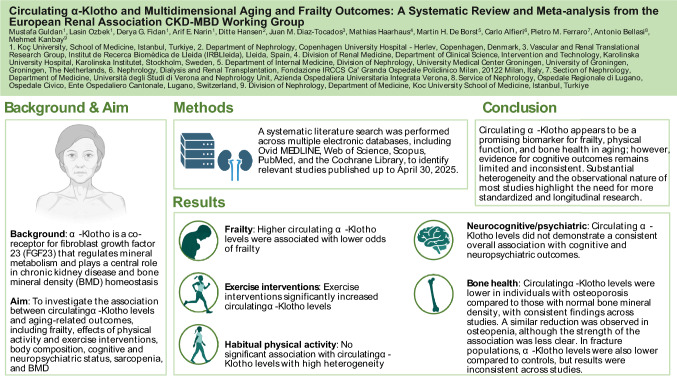

**Supplementary Information:**

The online version contains supplementary material available at 10.1007/s00223-026-01537-3.

## Introduction

Alpha-Klotho (α-Klotho), a recognized aging biomarker, was initially identified as an anti-aging gene in mice and serves as a co-receptor for fibroblast growth factor 23 (FGF23) [1, 2]. The kidneys are the principal source of α-Klotho, and chronic kidney disease (CKD) is consistently associated with reduced kidney expression and lower circulating levels [3, 4]. Beyond its central role in mineral metabolism and bone homeostasis, α-Klotho has been implicated in muscle regeneration, neuromuscular function, and cognitive resilience, highlighting its relevance in aging-related disorders [5–7]. Reduced α-Klotho, especially in CKD, has been associated with vascular calcification, sarcopenia, and increased mortality [5–8], underscoring its importance in populations vulnerable to accelerated aging and frailty.

Aging is a complex, multifactorial process characterized by progressive physiological decline, increased vulnerability to stressors, and a heightened risk of morbidity and mortality [9, 10]. Key changes include reductions in lean body mass (LBM), muscle strength, bone mineral density (BMD), and mobility, often accompanied by increased fat mass. These changes contribute to aging-associated disorders such as sarcopenia, frailty, and osteoporosis, as well as an overall functional decline, significantly impacting quality of life and increasing public health burdens [11].

Frailty, a clinical syndrome characterized by diminished physiological reserves and increased vulnerability to external stressors, is a critical concern in geriatric populations. Frailty, often assessed using the Fried Frailty Criteria (weight loss, exhaustion, grip strength, gait speed, and physical activity) [12], has traditionally been defined by physical decline and adverse outcomes in older adults but is now increasingly recognized as a multidimensional construct that also encompasses neuropsychiatric domains [13]. Depression, anxiety, and cognitive impairment are consistently linked with frailty [14], and these conditions may both accelerate and result from frailty through shared biological mechanisms, including chronic inflammation, hypothalamic–pituitary–adrenal axis dysregulation [15], highlighting the relevance of neuropsychiatric conditions to age-related outcomes and the broader conceptualization of frailty.

Previous studies suggest that higher circulating α-Klotho levels are associated with longevity, preserved muscle function, improved physical performance, and cognitive stability, whereas lower levels have been linked to frailty and functional decline [16–18]. However, there remains a lack of consensus in the literature on whether α-Klotho exerts consistent effects across diverse aging outcomes, mainly due to conflicting findings but also the predominantly cross-sectional design of most studies, limited longitudinal data, heterogeneity in outcome measures, focus on specific subpopulations (e.g., older adults, dialysis patients), absence of standardized thresholds for α-Klotho levels, and uncertainty surrounding potential confounders and underlying biological mechanisms.

Despite growing interest in α-Klotho as an aging biomarker, literature has primarily focused on its roles in kidney function, CKD, end-stage kidney disease, mineral and bone disorder, cardiovascular health, and mortality [7, 19], However, its associations across a broader aging-related outcomes, including frailty, sarcopenia, physical activity, cognitive decline, and neuropsychiatric conditions, remain inconsistent and understudied despite their relevance. Therefore, this study aims to systematically assess the relationship between circulating α-Klotho levels and frailty status, habitual physical activity, neurocognitive and neuropsychiatric conditions, musculoskeletal function, bone health, and body composition, to provide a multidimensional perspective on α-Klotho's role in aging and frailty. We also aim to determine whether exercise interventions can improve circulating α-Klotho levels.

## Materials and Methods

This meta-analysis was conducted in line with the Preferred Reporting Items for Systematic Reviews and Meta-Analyses (PRISMA) 2020 guidelines [20]. The study protocol was registered with PROSPERO (CRD42025630783).

### Search Strategy and Study Selection

We conducted a comprehensive literature search across databases, including Ovid MEDLINE, Web of Science, Scopus, PubMed, and the Cochrane Library, to identify peer-reviewed studies published up to 30 April 2025. The search strategy incorporated a set of keywords related to α-Klotho and outcome measures such as frailty, frailty components, and sarcopenia. A detailed description of the search strategy and keywords searched in each database is presented in Supplementary Table S1.

The title/abstract screening and full-text assessment of each article were independently performed by two authors with Covidence review management software based on predefined inclusion and exclusion criteria. Disagreements were settled by reaching a consensus, with a third author consulted when necessary. Furthermore, reference sections of eligible studies were manually screened to ensure the inclusion of relevant studies not captured by the literature search.

### Selection Criteria

Inclusion criteria for this meta-analysis are as follows: original peer-reviewed studies involving adult patients (≥ 18 years of age); studies reporting measurements of circulating α-Klotho levels with a clear stratification that allows a comparison; and studies evaluating the relationship between α-Klotho and predefined outcomes. Studies were excluded if they involved only urine or cerebrospinal fluid α-Klotho measurement, assessed unclear or unconventional frailty components, did not provide an objective frailty measurement, involved only pediatric populations, reported individual cases or case series, compared α-Klotho polymorphisms, lacked full-text English translation, or lacked relevant outcomes. Studies using overlapping datasets were excluded to ensure statistical independence; only the most comprehensive and up-to-date dataset was retained. Studies that did not provide extractable or comparable quantitative data (e.g., odds ratios, mean differences, or correlation coefficients) or did not clearly isolate α-Klotho-related outcomes were excluded from pooled analyses. To ensure consistency, circulating α-Klotho concentrations were converted to a uniform unit (pg/mL or ng/mL) across all included studies before pooling.

### Outcome Measures

The primary outcome of this meta-analysis was the association between circulating α-Klotho levels and frailty. Secondary outcomes included physical function parameters (e.g., grip strength, gait speed, short physical performance battery [SPPB], sit-to-stand test [STS], and 6-min walk test [6MWT]), body composition measures (body mass index [BMI], fat mass index [FMI]), physical activity and exercise interventions, bone health outcomes (osteopenia, osteoporosis, and fractures), and cognitive and neuropsychiatric outcomes.

### Quality Assessment

The quality of included studies was independently assessed by two authors using design-appropriate tools (Supplementary Tables S2–S6). Cohort and case–control studies were evaluated using the Newcastle–Ottawa Scale, which scores studies (0–9) based on selection, comparability, and outcome/exposure. Scores ≥ 7 were considered high, 4–6 moderate, and ≤ 3 low quality. Randomized controlled trials (RCTs) were assessed using the revised Cochrane Risk-of-Bias Tool (RoB 2), which evaluates five domains, including randomization and outcome reporting. For quasi-experimental and cross-sectional studies, the Joanna Briggs Institute (JBI) Critical Appraisal Checklists were applied, assessing key methodological criteria relevant to each design. None were excluded solely based on methodological quality. Instead, quality ratings were considered in interpreting the strength and reliability of the findings.

### Statistical Analysis

Meta-analyses were conducted using random-effects models to account for between-study variability. Pooled odds ratios (ORs) were calculated for dichotomous outcomes (e.g., presence or absence of frailty), and mean differences were used for continuous outcomes. Correlation coefficients (Pearson’s r) were synthesized using Fisher’s z-transformation. The heterogeneity of effect size was assessed using the Cochran Q test and the I^2^ statistic, where substantial heterogeneity was defined as values > 50% (Cochrane Handbook for Systematic Reviews of Interventions, version 5.3). Data analyses were conducted using Review Manager software (version 5.3, Cochrane Collaboration, London, UK; 2012). Sensitivity analyses were performed to assess the robustness of the pooled estimates and to identify potential sources of heterogeneity. A leave-one-out approach was applied, sequentially omitting each study at a time to evaluate its influence on the overall effect size and heterogeneity statistics.

## Results

**Fig. 1:**
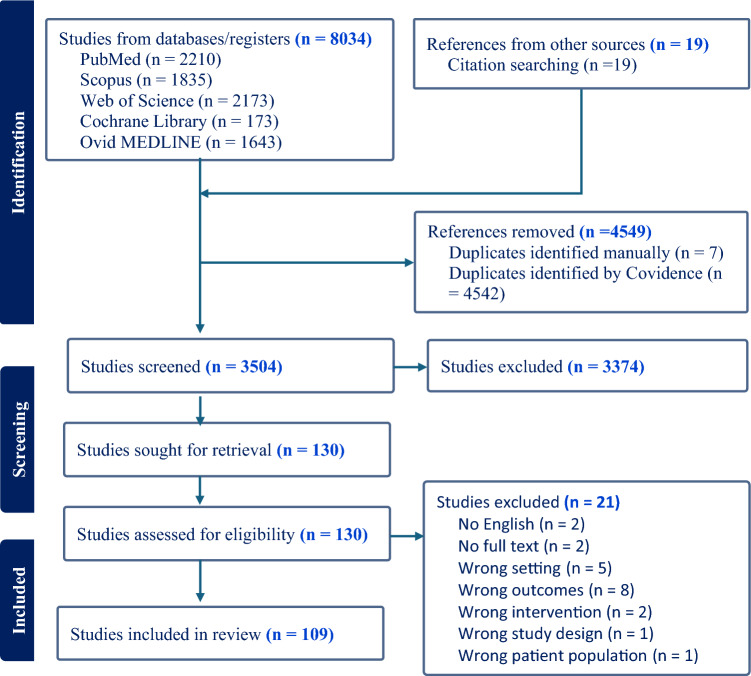
Flow diagram of the study selection process

The search identified 8,034 records from databases. An additional 19 studies were retrieved through manual searching. A final number of 109 studies were included in the synthesis (Fig. 1). The baseline characteristics of the included studies, full-text citations, outcome measures, methods of α-Klotho and outcome measurements, and key findings were outlined in Supplementary Tables S7–S10, respectively.

### Circulating α-Klotho and Frailty Status

**Fig. 2:**
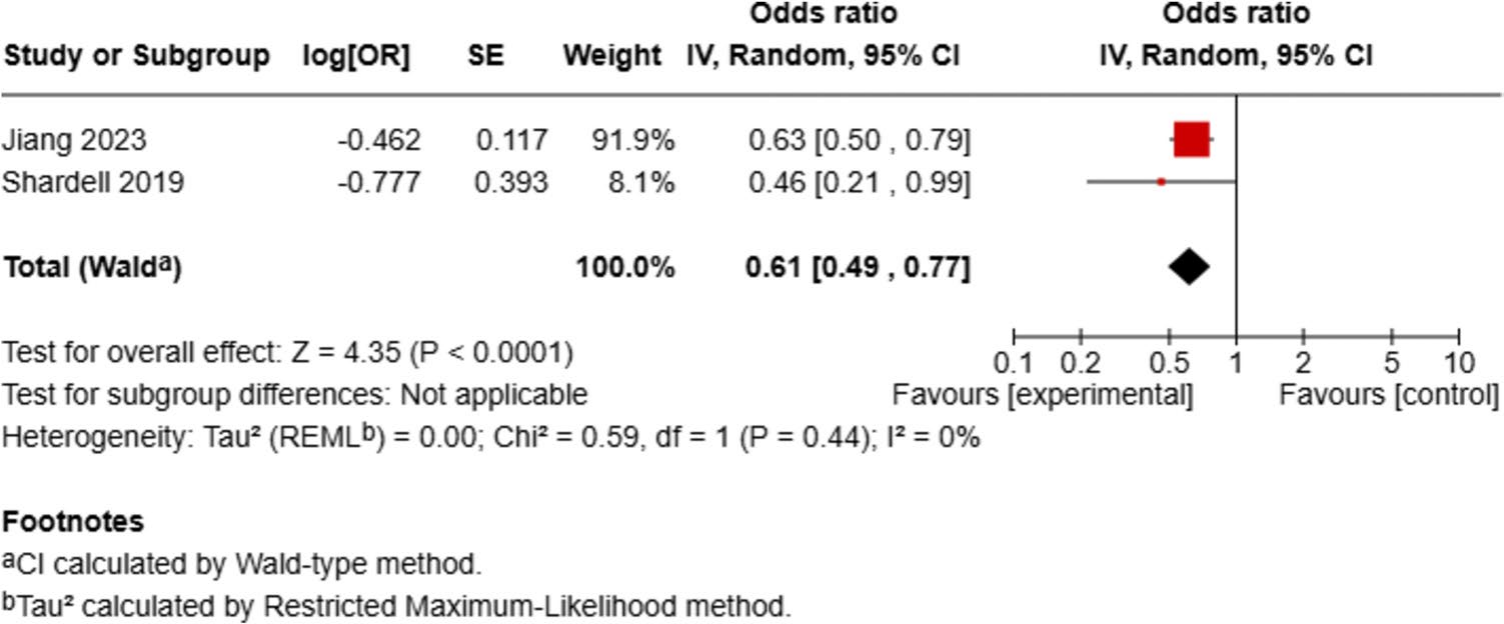
Meta-analysis of the association between circulating α-klotho levels and frailty. Odds ratios (ORs) with 95% confidence intervals are shown for each study and pooled using a random-effects model

A total of six studies investigating the association between circulating α-Klotho levels and frailty were included in this systematic review [11, 16, 21–24]. Of these, four studies provided sufficient data for pooled analysis using ORs. However, three of them were based on the same National Health and Nutrition Examination Survey (NHANES) dataset (2007–2016); thus, two studies were excluded to avoid duplication and ensure statistical independence. Only Jiang et al [11] was retained, as it provided the most comprehensive model and used continuous serum α-Klotho levels. Therefore, the analysis of two independent studies with a combined sample size of 7712 participants was included in the meta-analysis [11, 23]. Higher circulating α-Klotho levels were significantly associated with lower odds of frailty, with a pooled odds ratio of 0.61 (95% CI: 0.49, 0.77, *p* < 0.0001), with low heterogeneity (I^2^ = 0%) (Fig. 2).

Two studies comparing mean serum α-Klotho concentrations between frail and non-frail individuals were included in a separate meta-analysis [21, 22]. A total of 183 participants (104 frail, 79 non-frail) were analyzed using a random-effects model. The pooled mean difference in α-Klotho levels between frail and non-frail individuals was –0.04 ng/mL (95% CI: − 0.24, 0.16, *p* = 0.71), indicating no statistically significant difference between groups. Moderate heterogeneity was observed (I^2^ = 43%, *p* = 0.19), though not statistically significant (Supplementary Fig. S1). The pooled correlation between α-Klotho levels and frailty reported in two studies was r = − 0.18 (95% CI: − 0.32, − 0.02) [21, 22].

### Circulating α-Klotho and Exercise Interventions

**Fig. 3:**
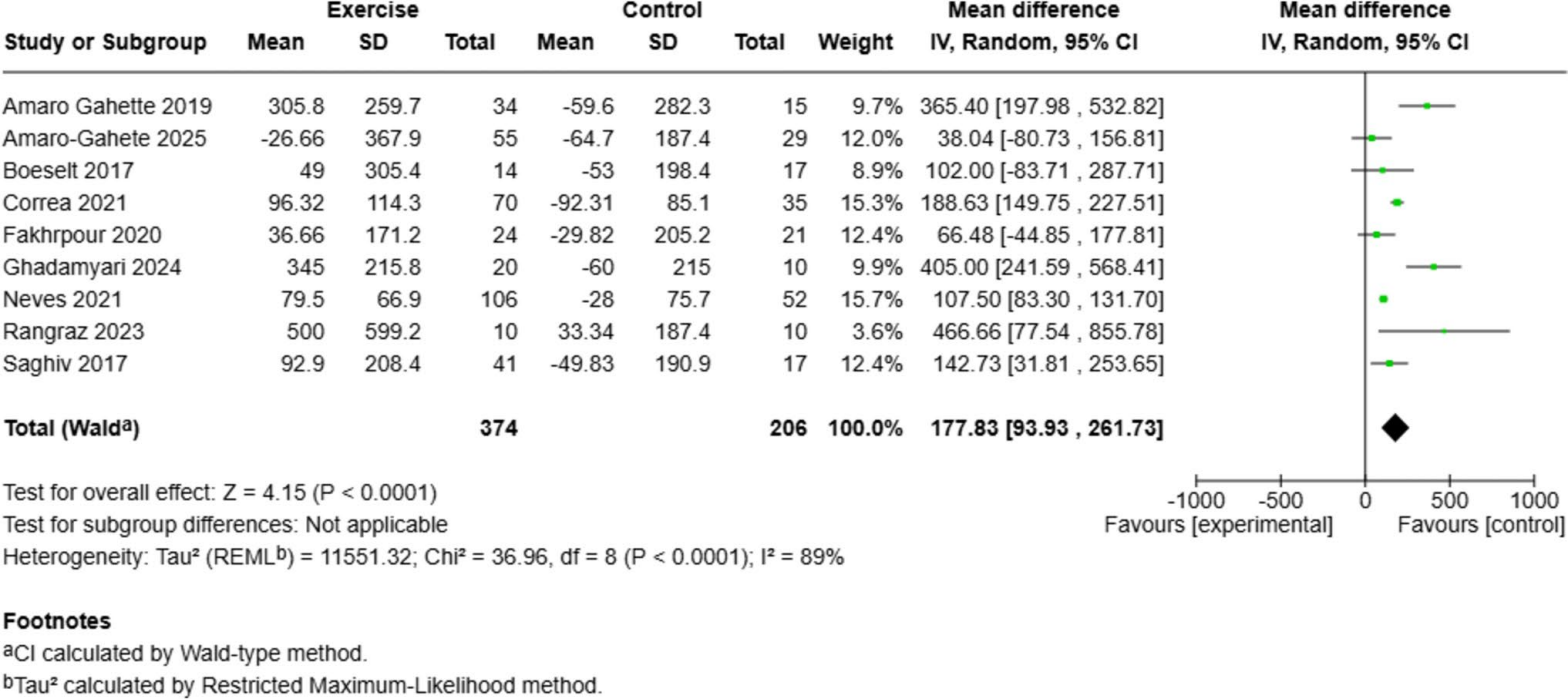
Meta-analysis of the effect of structured exercise interventions on circulating α-klotho levels. Mean differences (MDs) in circulating α-klotho levels (pg/mL) with 95% confidence intervals are shown for each study and pooled using a random-effects model

A meta-analysis of nine studies was conducted to assess the effect of exercise interventions on circulating α-Klotho levels [25–30]. The pooled analysis using a random-effects model showed statistically significantly higher circulating α-Klotho levels in the exercise groups compared to the control groups, with a mean difference of 177.83 pg/mL (95% CI: 93.93, 261.73; *p* < 0.0001). However, substantial heterogeneity was observed across studies (*p* < 0.0001; I^2^ = 89%) (Fig. 3). Due to heterogeneity, a sensitivity analysis with leave-one-out approach conducted. Sequential exclusion of individual studies did not alter the pooled mean difference and heterogeneity significantly. The overall effect size remained stable and statistically significant in all iterations. Subgroup analyses showed consistent effects across both healthy individuals and patients with chronic disease, with no significant differences between these groups (*p* for subgroup differences = 0.65). Similarly, while significant effects were observed for resistance and combined training but not for aerobic training alone, there were no significant differences between exercise modalities (*p* for subgroup differences = 0.65) (Supplementary Fig. S2).

### Circulating α-Klotho and Habitual Physical Activity

A meta-analysis of seven studies was conducted to compare circulating α-Klotho levels between physically active individuals and healthy inactive controls [31–37]. The overall pooled analysis using a random-effects model revealed a mean difference of 284.45 pg/mL (95% CI: − 199.15, 768.05; *p* = 0.25), indicating no statistically significant difference between the two groups. The analysis showed an I^2^ of 100% (*p* < 0.00001) significant (Supplementary Fig. S3).

### Circulating α-Klotho and Neuropsychiatric Conditions

A meta-analysis was conducted to compare circulating α-Klotho levels between individuals with neuropsychiatric conditions and those without, including data from 20 studies [38–57]. The pooled mean difference was − 4.84 ng/mL (95% CI: − 11.52, 1.84; *p* = 0.16), with high heterogeneity across studies (Supplementary Fig. S4). Subgroup analyses were conducted by running separate meta-analyses on distinct subsets (Supplementary Fig. S5–S10). The subgroup analysis showed that individuals with depression had significantly lower α-klotho levels than those without depression (mean difference =  − 0.13 ng/mL, 95% CI: − 0.26, − 0.01; *p* = 0.04), with high heterogeneity (I^2^ = 94%), and circulating α-klotho levels were higher in the schizophrenia group (mean difference = 0.29 ng/mL, 95% CI: 0.09, 0.50, *p* = 0.005), while all other categories, including major depressive disorder, neuroinflammatory, and bipolar disorder, showed non-significant differences. Subgroup analysis by different conditions and assay systems revealed markedly different results across subgroups (test for subgroup differences: *p* < 0.00001) (Supplementary Fig. S11). Therefore, the association between circulating α-klotho levels and the outcomes varied significantly depending on the study population and assay methodology.

**Fig. 4:**
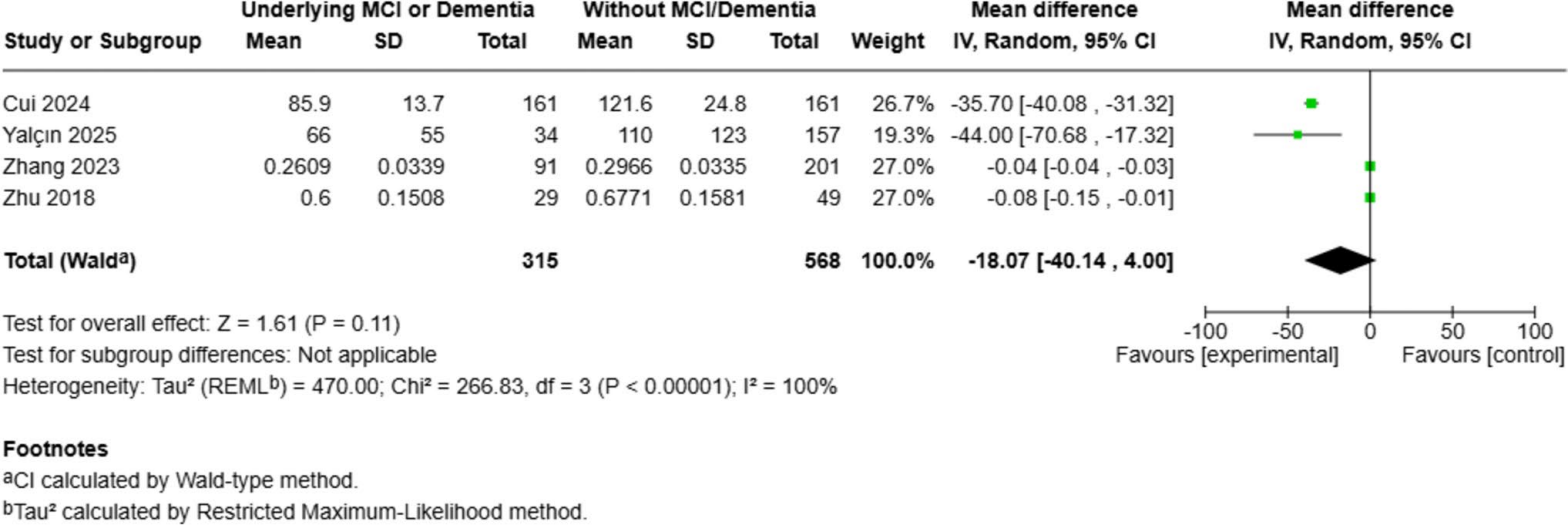
Meta-analysis of circulating α-klotho levels in individuals with mild cognitive impairment or dementia compared to controls. Mean differences (MDs) in circulating α-klotho levels (ng/mL) with 95% confidence intervals are shown for each study and pooled using a random-effects model

*Mild Cognitive Impairment & Dementia*. A separate analysis of four studies was performed to evaluate differences in circulating α-Klotho levels between individuals with and without underlying mild cognitive impairment or dementia [42, 55–57]. Using a random-effects model, the pooled analysis yielded a mean difference of − 18.07 ng/mL (95% CI: − 40.14, 4.00, *p* = 0.11), indicating that circulating α-Klotho levels were generally lower in individuals with cognitive impairment, though the result did not reach statistical significance. There was substantial heterogeneity among studies (Fig. 4). Leave-one-out sensitivity analysis showed that sequential exclusion of individual studies did not materially change the pooled estimate or the heterogeneity.

### Circulating α-Klotho, Musculoskeletal Function, and Aerobic Capacity

Twenty-six studies reported data on the relationship between circulating α-Klotho and bone-related parameters; 6 of them were eligible to be included in the meta-analysis.

**Fig. 5:**
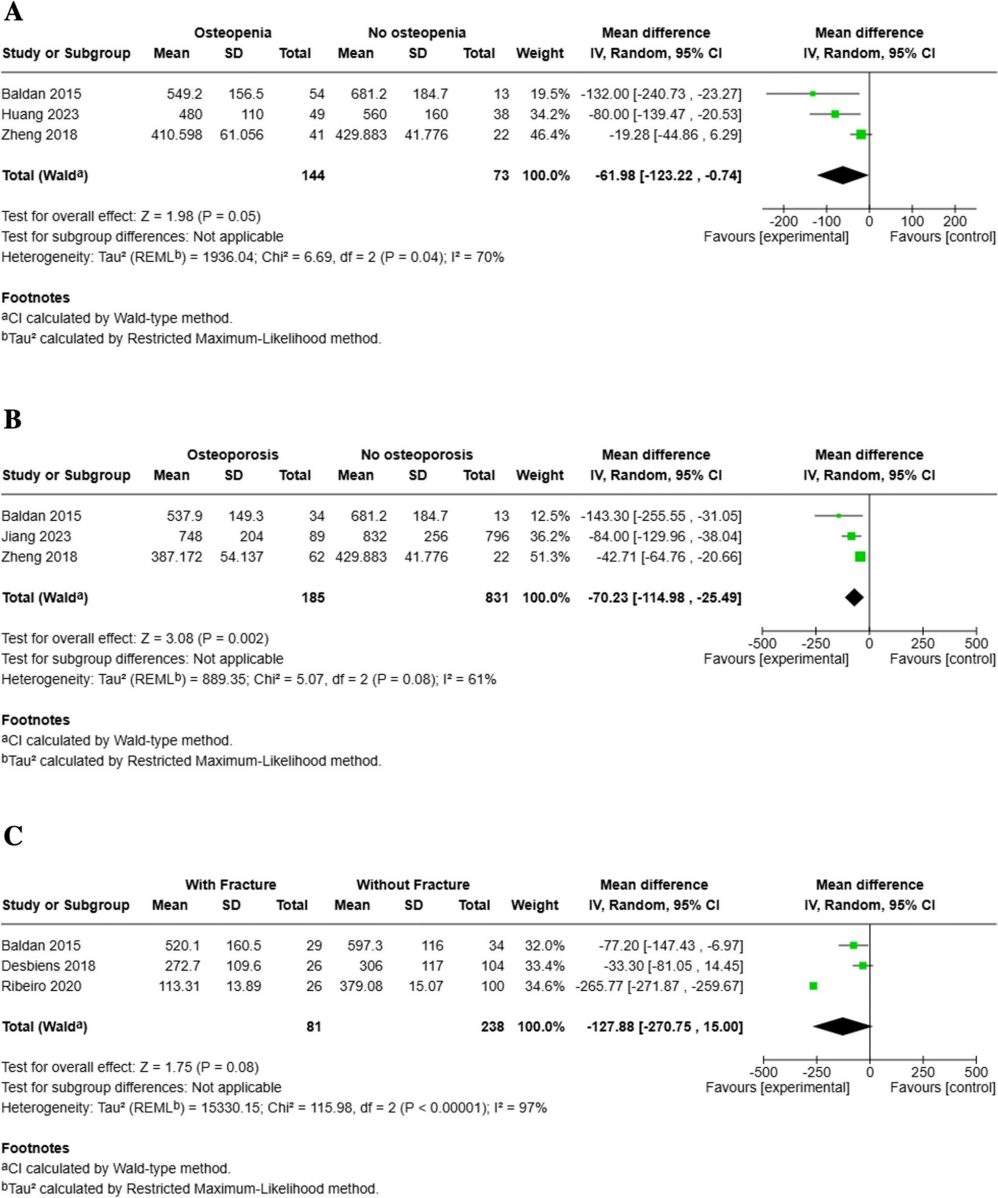
Meta-analysis of circulating α-klotho levels (pg/mL) in relation to bone health outcomes: **A)** osteopenia vs. non-osteopenia, **B)** osteoporosis vs. non-osteoporosis, and **C)** fracture vs. non-fracture populations. Mean differences (MDs) with 95% confidence intervals are shown for each study and pooled using a random-effects model

*Osteopenia.* Individuals in the osteopenia group had lower circulating α-Klotho levels compared to controls, with a pooled mean difference of − 61.98 pg/mL (95% CI: − 123.22, − 0.74), with borderline statistical significance (*p* = 0.05) in three studies combined [58–60] (Fig. 5A). Sensitivity analysis using the leave-one-out approach revealed that exclusion of Zheng et al. (2018) (60), reduced heterogeneity from 70 to 0% and strengthened the pooled effect (MD − 91.97, 95% CI: − 144.15, − 39.80; *p* < 0.001) (Supplementary Fig. S12).

*Osteoporosis*. Three studies reported α-Klotho levels for individuals with osteoporosis versus without osteoporosis. Individuals with osteoporosis had significantly lower α-Klotho levels compared to those with normal BMD, with a pooled mean difference of − 70.23 pg/mL (95% CI: − 114.98, − 25.49, *p* = 0.002), and moderate heterogeneity was observed (I^2^ = 61%) (Fig. 5B) [11, 58, 60]. Sensitivity analysis excluding Zheng et al., (2018) (60) reduced heterogeneity from 61 to 0% and strengthened the pooled effect (MD − 92.52, 95% CI: − 135.05, − 49.98; *p* < 0.001) (Supplementary Fig. S12).

*Fractures*. A total of 81 of individuals with fractures were identified in 3 studies with 319 participants. Individuals with fractures had lower circulating α-Klotho levels compared to those without fractures, with a pooled mean difference of − 127.88 pg/mL (95% CI: − 270.75, 15.00), although the result did not reach quite statistical significance (*p* = 0.08). High heterogeneity was observed across studies (I^2^ = 97%) (Fig. 5C) [58, 61, 62]. Sensitivity analysis excluding Ribeiro et al. [61], who demonstrated a considerably greater reduction of α-Klotho in patient with fractures than the other two studies, reduced heterogeneity (I^2^ = 3%) and yielded a significant pooled estimate (MD − 47.39, 95% CI − 87.55 to − 7.22; *p* = 0.02) (Supplementary Fig. S12).

### Qualitative Analysis

Due to substantial heterogeneity in outcome definitions and reporting across studies, meta-analyses were not feasible for 6MWT, grip strength, SPPB, gait speed, STS and body composition, and these outcomes were therefore evaluated through qualitative synthesis.

*Six-minute walk test.* Three studies assessed the association between circulating α-Klotho levels and walking endurance using the 6MWT [5, 22, 63]. In Valenzuela et al. [63], participants were dichotomized into low and high α-Klotho groups based on the sample median of 369 pg/mL. The study reported a significant association between low plasma α-Klotho levels and impaired 6MWT performance (OR = 11.0, 95% CI: 2.0, 60.6, *p* = 0.003) [63]. Arroyo et al [5] reported a Pearson correlation of r = 0.26 (*p* = 0.022) between serum α-Klotho and 6MWT distance. Sanz et al. [22] found no significant association, reporting r = − 0.017 (*p* = 0.87) between serum α-Klotho levels and 6MWT distance in a cohort of older adults.

*Grip strength.* Seven studies were included that assessed the association between circulating α-Klotho levels and physical function, including grip strength, in diverse populations: four observational studies in healthy or community-dwelling older adults [5, 21, 22, 64, 65], one study in patients with β-thalassemia major [58], and one in individuals with chronic conditions, specifically chronic kidney disease/dialysis [63]. Across studies, grip strength was assessed using hand dynamometry, and a narrative synthesis was preferred over meta-analysis due to the various reporting of effect measures.

Among eight studies, six reported a significant positive association between circulating α-Klotho levels and grip strength in older or clinical populations, while two found no such relationship. In a large cohort study of 1,983 participants conducted by Semba et al. [64], higher α-Klotho concentrations were also associated with a reduced decline in grip strength over time (β =  − 0.0061, SE = 0.0021, *p* = 0.005). In their previous study of a large cohort of 804 community-dwelling older adults, Semba et al. [17] reported that in those with plasma α-Klotho < 681 pg/mL, a one standard deviation increase in plasma α-Klotho levels was associated with a 1.20 kg higher grip strength (β = 1.20, SE = 0.35, *p* = 0.0009), adjusted for covariates. In contrast, Polat et al. [21] (N = 89) reported a weak and non-significant correlation between serum α-Klotho and grip strength (r = 0.117, *p* = 0.270) in the elderly. Similarly, Sanz et al., [22] (N = 103) found no significant correlation between serum α-Klotho and grip strength (r = 0.025, *p* = 0.805), though α-Klotho was positively associated with functional independence (Barthel Index, r = 0.253, *p* = 0.010) and inversely associated with the number of falls (Poisson β =  − 3.311, *p* < 0.001) in nursing home residents.

In a healthy adult population spanning a wide age range, Arroyo et al. [5] (N = 80) reported a small, non-significant positive correlation between serum α-Klotho levels and grip strength (r = 0.15, *p* = 0.19). Valenzuela et al. [63] (N = 30) found that elderly dialysis patients with lower plasma α-Klotho levels (< 369 pg/mL) had significantly higher odds of exhibiting low handgrip strength (OR = 5.5x, 95% CI: 1.15, 26.41, *p* = 0.028), and that poor grip strength predicted increased mortality risk (RR = 3.0x, 95% CI: 1.01, 8.95, *p* = 0.025). A positive association of α-Klotho plasma levels with grip strength is shown in Amaro-Gahete et al. [65]. Finally, Baldan et al. [58] found that plasma α-Klotho levels were significantly lower in β-thalassemia major patients than in controls and were directly correlated with handgrip strength (significant at *p* = 0.01, N = 106).

*Short Physical Performance Battery (SPPB)*. Three studies report a consistent positive association between circulating α-Klotho levels and physical function as measured by the SPPB, a validated composite score of balance, gait speed, and chair stand tests [22, 66, 67].

In the Shardell et al. [66] study using the InCHIANTI cohort, higher plasma α-Klotho concentrations were significantly associated with higher SPPB scores across all models. In the fully adjusted model (Model 3), a one-unit increase in ln(α-Klotho) was associated with a 0.51-point increase in SPPB score (95% CI: 0.11, 0.91, *p* = 0.01), controlling for demographics, nutritional status, comorbidities, cognitive function, and parathyroid hormone levels [66]. Similarly, based on the same cohort, Crasto et al. [67] found that participants in the lowest tertile of plasma α-Klotho had significantly poorer SPPB scores. Among 802 older adults in the InCHIANTI study, 18.8% of those in the lowest tertile had SPPB scores < 6 (vs. 8.6% in the highest tertile, *p* = 0.02). In contrast, Sanz et al. (2021) found no significant correlation between serum α-Klotho levels and SPPB scores in a sample of 98 older adults (r = 0.025, *p* = 0.806) 8.6% in the highest tertile, *p* = 0.02) [67]. In contrast, Sanz et al. [22] found no significant correlation between serum α-Klotho levels and SPPB scores in a sample of 98 older adults (r = 0.025, *p* = 0.806).

*Gait speed*. Three studies examined the association between circulating α-Klotho levels and gait speed in older adults and clinical populations; however, none found a statistically significant relationship [5, 21, 22]. Arroyo et al. [5] (N = 80) assessed usual gait speed in healthy adults across the lifespan and reported a small, non-significant positive correlation with serum α-Klotho levels (r = 0.06, *p* = 0.63). Similarly, Sanz et al. [22] (N = 103), who evaluated 4-m gait speed in older adults, found no meaningful correlation between gait speed and serum α-Klotho (r =  − 0.009, *p* = 0.927), suggesting no linear association in a community-based sample. Polat et al. [21] (N = 89) also reported a non-significant correlation (r =  − 0.131, *p* = 0.135) between walking speed and serum α-Klotho in geriatric outpatients.

*Sit-to-stand tests (STS).* Three studies reported an association between circulating α-Klotho levels and STS tests [5, 63, 66]. In a study by Valenzuela et al. [63] involving 30 elderly male dialysis patients, lower plasma α-Klotho levels (< 369 pg/mL) were significantly associated with poorer performance in the STS test (OR = 26.0, 95% CI: 3.69, 183.42, *p* < 0.001). Moreover, low STS performance predicted increased all-cause mortality over 18 months (risk ratio [RR] = 3.0, 95% CI: 1.01, 8.95, *p* = 0.025), indicating its prognostic value beyond biomarker levels. Similarly, Shardell et al. [66] showed that in minimally adjusted models, ln(α-Klotho) was significantly associated with higher chair stand scores (β = 0.17, *p* = 0.04), though the association weakened after full adjustment for comorbidities, cognition, and nutritional factors (*p* = 0.09). Lastly, findings from Arroyo et al. [5] did not show a statistically significant correlation between klotho levels and chair stands completed in 30 s and time to complete 5 chair stands (r = -0.10, *p* = 0.39) among healthy adults.

*Others*. Semba et al. [64] also found that individuals in the highest tertile of plasma α-Klotho had significantly higher knee extensor strength at baseline compared to those in the lowest tertile (β = 0.72, SE = 0.018, *p* < 0.0001), even after adjusting for age, sex, race, study site, inflammation markers (c-reactive protein [CRP], interleukin [IL-6]), and diabetes. More importantly, over four years of follow-up, participants with higher baseline α-Klotho experienced significantly less decline in normalized knee strength (β =  − 0.025, SE = 0.010, *p* = 0.02), suggesting that α-Klotho may play a protective role in the maintenance of muscle function. Stratified analyses indicated that this effect was more pronounced in men and participants with lower systemic inflammation (IL-6 < 2.5 pg/mL).

### Circulating α-Klotho and Body Composition

Ten studies were included in this narrative synthesis [5, 68–76]. Collectively, the studies demonstrate that higher circulating α-Klotho levels are positively associated with favorable body composition, particularly increased lean mass and reduced fat mass. In a cross-sectional study by Amaro-Gahete et al. [68], involving 74 sedentary middle-aged adults, plasma α-Klotho levels were strongly associated with lean mass index (LMI; β = 74.8, *p* < 0.001), independent of FMI, age, and sex. Associations with BMI and BMD were also observed initially, but lost significance after adjusting for LMI. In their subsequent 12-week RCT (n = 74), the same group found that all exercise interventions led to increased α-Klotho, with positive associations between increases in LMI and plasma α-Klotho levels, and inverse associations with reductions in FMI.

A large cross-sectional analysis by Xie et al. [69] using NHANES data (n = 3,803; ages 40–59) further supports these results. Serum α-Klotho was inversely associated with low muscle mass risk (OR = 0.81, 95% CI: 0.71, 0.92), defined by appendicular lean mass adjusted for BMI (ALM/BMI), with stronger effects in women. ALM/BMI increased across α-Klotho tertiles, and restricted cubic spline analysis confirmed a linear dose response. Similarly, in a behavioral intervention study of 152 adults with overweight/obesity, Collins [70] found that participants achieving ≥ 10% weight loss over 6 months had significant increases in serum α-Klotho levels, which were inversely correlated with reductions in fat mass (r = − 0.18, *p* = 0.026) and waist circumference (r = − 0.22, *p* = 0.008).

In healthy aging populations, α-Klotho may also track with body composition. Arroyo et al. [5] evaluated 80 adults across four age groups and reported that α-Klotho levels were significantly lower in individuals aged 50–64 and ≥ 65 compared to those aged 20–34. α-Klotho was negatively correlated with body fat percentage (r = − 0.24, *p* = 0.037) and positively with 6MW distance (r = 0.26, *p* = 0.022), though no differences were seen between high vs. low performers in grip strength or chair stand tests. Complementing this, Salamanna et al. [71] found that in 18 spinal surgery patients, serum klotho is significantly lower in osteopenic, sarcopenic, and osteosarcopenic patients than in controls, with osteosarcopenic patients having the lowest concentration; however, the age difference between the patients and the controls was wide. In contrast, Fukasawa et al. [72] studied 77 hemodialysis patients and found no significant correlation between plasma α-Klotho and abdominal muscle area (r = 0.032; *p* = 0.78) or creatinine production (r = 0.061, *p* = 0.60), used as muscle mass proxies, suggesting α-Klotho may be less reflective of body composition in advanced chronic kidney disease.

## Discussion

This systematic review and meta-analysis comprehensively examined the associations between circulating α-Klotho levels and a range of aging-related health outcomes, including frailty, frailty components, physical function, exercise response, cognition, and body composition. Our key findings include that higher circulating α-Klotho levels were significantly associated with lower odds of frailty and better physical performance, including stronger grip strength and higher SPPB scores, though associations with gait speed were inconsistent; however, differential study designs and study populations impaired optimum pooling, requiring further evidence on the issue. Furthermore, exercise interventions robustly increased circulating α-Klotho levels, while habitual physical activity showed no significant effect. Higher circulating α-Klotho concentrations were also linked to more favorable body composition, characterized by increased lean mass and reduced fat mass.

Several studies have demonstrated that circulating α-Klotho levels increase following structured (12–16 week) exercise interventions. However, our study showed no significant differences between habitual long-term exercisers, such as professional athletes, and sedentary individuals. This suggests that circulating α-Klotho may be more acutely responsive to recent bouts of exercise rather than sustained habitual activity. One possible explanation is that increased circulating α-Klotho levels may reflect a transient adaptive response to physical stress, oxidative balance, or metabolic demand triggered by new or intensified exercise stimuli, which may diminish with long-term physiological adaptation in chronically trained individuals. A previous systematic review and meta-analysis by Corrêa et al. [77] found that exercise interventions spanning a minimum of 12 weeks significantly increase circulating α-Klotho levels, with effects influenced by training duration, volume, and intensity, except in combined aerobic and resistance protocols. However, these discrepancies may also be attributed to selection bias and differences in study design between the exercise intervention and habitual physical activity studies.

The findings from the meta-analysis highlight a clear association between reduced circulating α-Klotho levels and compromised bone health. Individuals with osteopenia and osteoporosis consistently exhibited lower α-Klotho levels compared to healthy controls, supporting the hypothesis that α-Klotho deficiency may contribute to the pathophysiology of bone mineral loss. The trend observed in osteopenia suggests that the reduction in circulating α-Klotho levels may begin early in the trajectory of bone deterioration, potentially serving as an early biomarker for skeletal fragility. However, the included study populations were heterogeneous. In cohorts that include hemodialysis or advanced CKD patients, the observed reductions may reflect renal impairment rather than bone status itself or may alternatively reflect the role of klotho in bone anabolism. In contrast, in populations without overt CKD (postmenopausal osteoporosis, β-thalassemia), the association is less likely to be explained by eGFR alone and may reflect disease-specific mechanisms. These contexts suggest that the observed association cannot be attributed to a single pathway: in postmenopausal women, age-related and hormonal influences likely predominate; in β-thalassemia, chronic inflammation and endocrine dysfunction may underlie the decrease; and in hemodialysis, impaired renal production is the key driver. Thus, while diminished klotho levels consistently accompany low bone mass, the mechanisms appear population-specific, and low klotho may represent a convergent biomarker of skeletal vulnerability rather than a uniform causal factor.

In osteoporosis, the association appeared more pronounced and consistent across studies, reinforcing the biological plausibility of α-Klotho’s role in maintaining bone integrity through its influence on mineral metabolism, oxidative stress, and inflammation [78]. Although a similar reduction in circulating α-Klotho levels was observed in individuals with fractures, the results were not statistically conclusive, likely due to high variability across studies. The sensitivity analysis, removing the study by Ribeiro et al. [60], rendered a considerable reduction of variability and revealed a significant association of lower klotho with increased fracture risk. This heterogeneity could in fact be attributed to a considerably larger difference in α-Klotho levels between groups in the study by Ribeiro et al. [60] than in the other two studies reporting fractures. Thus, while the study clearly confirms the association of lower circulating α-Klotho with increased fracture risk, its addition to the meta-analysis caused a disproportional increase of the confidence interval. In osteoporosis, the association appeared more pronounced and consistent across studies, reinforcing the biological plausibility of α-Klotho’s role in maintaining bone integrity through its influence on mineral metabolism, oxidative stress, and inflammation [78]. Although a similar reduction in circulating α-Klotho levels was observed in individuals with fractures, the results were not statistically conclusive, likely due to high variability across studies. The sensitivity analysis, removing the study by Ribeiro et al. [60], rendered a considerable reduction of variability and revealed a significant association of lower klotho with increased fracture risk. This heterogeneity could in fact be attributed to a considerably larger difference in α-Klotho levels between groups in the study by Ribeiro et al. [60] than in the other two studies reporting fractures. Thus, while the study clearly confirms the association of lower circulating α-Klotho with increased fracture risk, its addition to the meta-analysis caused a disproportional increase of the confidence interval.

Collectively, these findings suggest that α-Klotho has potential as a biomarker for bone health deterioration, particularly in the context of osteopenia and osteoporosis. However, the inconclusive evidence in fracture outcomes underscores the need for more standardized methodologies and longitudinal data to better understand the temporal relationship between α-Klotho levels and bone fragility events. Overall, the role of α-Klotho in bone metabolism and its association with osteopenia and osteoporosis further underscores its relevance to frailty risk.

A key strength of this meta-analysis is its comprehensive synthesis of the relationship between α-Klotho and frailty, encompassing both the overall association and specific frailty components. To our knowledge, this is the first meta-analysis to comprehensively synthesize evidence on circulating α-Klotho across interconnected domains, including frailty, physical activity, neuropsychiatric and cognitive outcomes, musculoskeletal function, and body composition, providing an integrated view of α-Klotho’s potential role within the complex, multidimensional aging phenotype.

### Limitations

A notable limitation of this meta-analysis is the substantial heterogeneity among the included studies, both in terms of study design and the reporting of effect measures, which limited the ability to perform a robust quantitative synthesis. The study populations varied widely, from older adults in geriatric cohorts to individuals with chronic kidney disease, patients on hemodialysis, and even middle-aged adults, further contributing to the variability. While the analysis aimed to provide a broad and detailed overview of the relationship between α-Klotho and frailty, including its cognitive, neuropsychiatric, musculoskeletal, body composition, and bone-related components, the number of studies directly assessing the α-Klotho–frailty relationship was relatively small and methodologically diverse. This limited the capacity to draw definitive conclusions about a direct, consistent association between α-Klotho levels and frailty across populations. Furthermore, the direct evidence on the frailty and klotho relationship was very limited. Instead, we have conducted separate meta-analyses regarding each domain of frailty, including physical and cognitive aspects, to better evaluate the klotho and frailty-related concepts. Additionally, most included studies focused on established frailty or advanced clinical populations, with limited data on the association between α-Klotho and pre-frailty or early functional decline, which may be more amenable to intervention. Additionally, given that α-Klotho is a circulating protein influenced by a range of physiological and environmental factors, including inflammation, kidney function, nutritional status, and potential gender or ethnic differences, its levels are subject to substantial variability. Although several studies adjusted for these confounding variables, inconsistencies in how these factors were measured and controlled may have contributed to the heterogeneity observed across the literature and the variability in reported associations with frailty. Finally, it is also important to acknowledge the variability in assay methods used to measure circulating α-Klotho levels across studies. Differences in assay type (e.g., ELISA kits from various manufacturers), sample handling, and detection sensitivity can introduce measurement inconsistencies that affect comparability. These analytical variations and lack of specific thresholds may contribute to the heterogeneity observed in pooled analyses and highlight the need for standardized, validated protocols for soluble α-Klotho assessment in clinical research.

### Future Perspectives

Given the limited number of studies directly examining the α-Klotho–frailty relationship, we have made our best efforts to utilize multiple domains and concepts related to frailty in this meta-analysis to better understand the klotho-frailty relationship. However, future studies examining the relationship between klotho and frailty to a greater extent are required. Particularly those focusing on early or preclinical stages of frailty. Stratified analyses, especially by age, but also sex, and ethnicity, could also clarify potential differential effects. Secondly, interventional studies examining whether increasing circulating α-Klotho levels, through pharmacological, nutritional, or lifestyle means, can prevent or mitigate frailty would provide critical insight into its therapeutic potential. Finally, future studies should further explore the potential role of α-Klotho in neuropsychiatric conditions, particularly given the observed trend toward lower levels in individuals with mild cognitive impairment and dementia. Additional research is warranted to clarify α-Klotho’s relevance across specific conditions, such as schizophrenia, where differential effects may exist. Importantly, exploration in CKD is particularly warranted, as reduced α-Klotho expression is a hallmark of CKD pathophysiology and is strongly implicated in premature aging, vascular calcification, and sarcopenia. Frailty is highly prevalent in this population and has direct prognostic implications for hospitalization, dialysis outcomes, and transplantation eligibility. Investigating α-Klotho within CKD cohorts may therefore not only illuminate mechanistic links between kidney dysfunction, accelerated aging, and frailty, but also identify potential therapeutic targets to improve both survival and quality of life in this vulnerable group.

## Conclusion

This meta-analysis highlights a potential link between reduced α-Klotho levels, frailty, reduced bone mineral content, and fracture risk, suggesting that α-Klotho may play a role in the complex biological pathways underlying age-related decline. Overall, this meta-analysis offers a multidimensional perspective, positioning α-Klotho as a central biomarker in the complex pathophysiology of frailty. Although the evidence supports associations with various frailty components, such as cognitive impairment, musculoskeletal deterioration, adverse body composition, and bone loss, the overall findings are limited by heterogeneity in study populations, methodologies, and outcome measures. While circulating α-Klotho shows promise as a biomarker and possible therapeutic target, further high-quality, longitudinal, and mechanistic studies are needed to confirm its role in frailty development and progression. A more standardized and nuanced approach to studying α-Klotho in the context of aging could offer valuable insights into early detection and intervention strategies for frailty for the future.

## Supplementary Information

Below is the link to the electronic supplementary material.

Supplementary file1 (DOCX 1839 KB)

## Data Availability

This study did not generate any original data. All information analyzed is publicly available in the literature and has been cited accordingly within the manuscript.
