# Supplementary material for: Circulating α-Klotho and Multidimensional Aging and Frailty Outcomes: A Systematic Review and Meta-Analysis from the European Renal Association CKD-MBD Working Group
Source: Calcif Tissue Int. 2026 Apr 30;117(1):72. doi: 10.1007/s00223-026-01537-3 (PMC13132909; doi:10.1007/s00223-026-01537-3)
Supplement: Supplementary file 1 — Supplementary file1 (DOCX 1839 KB) [file 223_2026_1537_MOESM1_ESM.docx]

**Supplementary Material – Table of Contents**

**Supplementary Table S1**. Search strategy and keywords for systematic database search

**Supplementary Table S2**. Quality assessment of cross-sectional studies using the JBI critical appraisal tool

**Supplementary Table S3**. Quality assessment of cohort studies using the Newcastle–Ottawa Scale (NOS)

**Supplementary Table S4**. Quality assessment of case-control studies using the Newcastle–Ottawa Scale (NOS)

**Supplementary Table S5**. Quality assessment of randomized controlled trials using the Cochrane Risk-of-Bias 2 tool

**Supplementary Table S6**. Quality assessment of quasi-experimental studies using the JBI critical appraisal tool

**Supplementary Table S7**. Baseline characteristics of included studies

**Supplementary Table S8**. Full citations of included studies

**Supplementary Table S9**. Summary of the outcome measures and methods of measurement employed in the included studies.

**Supplement Table S10**. Summary of the key findings in the included studies.

**Supplementary Figure S1**. Meta-analysis of the association between circulating α-klotho levels and frailty (frail vs. non-frail groups)

**Supplementary Figure S2**. Subgroup analyses of exercise effects on circulating α-klotho levels (A) by population (healthy vs. chronic disease) (B) by exercise modality (aerobic, resistance, combined)

**Supplementary Figure S3**. Meta-analysis of the association between habitual physical activity and circulating α-klotho levels

**Supplementary Figure S4**. Meta-analysis of circulating α-klotho levels in individuals with and without neuropsychiatric conditions

**Supplementary Figure S5**. Meta-analysis of circulating α-klotho levels across neuropsychiatric subgroups (detailed in Supplementary Figures S6–S10)

**Supplementary Figure S6.** Meta-analysis of circulating α-klotho levels in individuals with versus without bipolar disorder

**Supplementary Figure S7**. Meta-analysis of circulating α-klotho levels in individuals with versus without depression

**Supplementary Figure S8**. Meta-analysis of circulating α-klotho levels in individuals with versus without schizophrenia

**Supplementary Figure S9**. Meta-analysis of circulating α-klotho levels in individuals with versus without neuroinflammatory conditions

**Supplementary Figure S10**. Meta-analysis of circulating α-klotho levels in individuals with versus without dementia, mild cognitive impairment, Alzheimer’s disease, and Parkinson’s disease dementia

**Supplementary Figure S11**. Meta-analysis of circulating α-klotho levels across subgroups defined by ELISA assay type and clinical outcomes

**Supplementary Figure S12**. Sensitivity analyses for osteopenia (A), osteoporosis (B), and fractures (C)

**Supplementary Table S1**. Search strategy and keywords for systematic database search.

| **Total Records: n= 8034** |
| --- |
| - After Delete of Duplicate Records (Covidence and manual identification): **n=3503** (n=4542 duplicates identified by Covidence, n=7 duplicates identified manually) |
| **PubMed (n=2210)** |
| **7** ((Klotho[Title/Abstract] OR S-Klotho[Title/Abstract] OR α-Klotho[Title/Abstract] OR alpha-Klotho[Title/Abstract]) OR ("Klotho Proteins"[Mesh])) AND ((Frail*[Title/Abstract] OR frailness[Title/Abstract] OR "Muscular Weakness"[Title/Abstract] OR "biological aging"[Title/Abstract] OR frail*[Title/Abstract] OR "sarcopenia"[Title/Abstract] OR "sarcopenic"[Title/Abstract] OR Debilit*[Title/Abstract] OR muscle[Title/Abstract] OR "Body Composition*"[Title/Abstract] OR "hand strength"[Title/Abstract] OR "grip strength"[Title/Abstract] OR Grasp[Title/Abstract] OR aging[Title/Abstract] OR aged[Title/Abstract] OR elderly[Title/Abstract] OR Older[Title/Abstract] OR elder[Title/Abstract] OR prefrailty[Title/Abstract] OR walking speed[Title/Abstract] OR vulnerable[Title/Abstract] OR vulnerability[Title/Abstract] OR pre-frail[Title/Abstract] OR multimorbid[Title/Abstract] OR multimorbidity[Title/Abstract] OR gait disorders[Title/Abstract] OR aged, 80[Title/Abstract] OR cognition[Title/Abstract] OR prefrail[Title/Abstract] OR geriatric[Title/Abstract] OR geriatry[Title/Abstract] OR aged, 60[Title/Abstract] OR Physical Fitness[Title/Abstract] OR Cardiorespiratory Fitness[Title/Abstract] OR Functional Performance[Title/Abstract] OR Physical Performance[Title/Abstract] OR Postural Balance[Title/Abstract] OR Balance[Title/Abstract] OR flexibility[Title/Abstract] OR agility[Title/Abstract] OR Physical Endurance[Title/Abstract] OR Walking Speed[Title/Abstract] OR geriatric syndrome[Title/Abstract] OR geriatric disorder nutritional assessment[Title/Abstract] OR fragility[Title/Abstract] OR Gait Speed[Title/Abstract] OR Walking speed[Title/Abstract] OR Short Physical Performance Battery[Title/Abstract] OR old adult[Title/Abstract] OR Functionally-Impaired[Title/Abstract] OR Functionally Impaired[Title/Abstract] OR functional impairment[Title/Abstract] OR function decline[Title/Abstract] OR Decreased Physiologic Reserve[Title/Abstract] OR oldest adults[Title/Abstract] OR old population[Title/Abstract] OR oldest population[Title/Abstract] OR old individuals[Title/Abstract] OR oldest individuals[Title/Abstract] OR old patients[Title/Abstract] OR oldest patients[Title/Abstract] OR old persons[Title/Abstract] OR disability[Title/Abstract] OR disabled[Title/Abstract] OR physical disability[Title/Abstract] OR disabilit*[Title/Abstract] OR disable[Title/Abstract] OR activities of daily living[Title/Abstract] OR fat free mass[Title/Abstract] OR lean mass[Title/Abstract] OR Malnutrition[Title/Abstract] OR muscle function[Title/Abstract] OR muscular strength[Title/Abstract] OR muscular weakness[Title/Abstract] OR "handgrip strength"[Title/Abstract] OR lean body mass[Title/Abstract] OR muscular atrophy[Title/Abstract] OR physical function[Title/Abstract] OR pre-sarcopenia[Title/Abstract] OR presarcopenia[Title/Abstract] OR cachexia[Title/Abstract] OR cachectic[Title/Abstract] OR wasting[Title/Abstract] OR malnutrition[Title/Abstract] OR malnourish[Title/Abstract] OR undernutrition[Title/Abstract] OR undernourished[Title/Abstract] OR malnutrition[Title/Abstract] OR undernutrition[Title/Abstract] OR malnourishment[Title/Abstract]) OR ("Aging"[Mesh] OR "Aged"[Mesh] OR "Hand Strength"[Mesh] OR "Body Composition"[Mesh] OR "Muscles"[Mesh] OR "Frailty"[Mesh] OR "Sarcopenia"[Mesh] OR "Muscle Weakness"[Mesh] OR "Muscle, Skeletal"[Mesh] OR "Frail Elderly"[Mesh] OR "Walking Speed"[Mesh] OR "Multimorbidity"[Mesh] OR "Cognition"[Mesh] OR "Physical Fitness"[Mesh] OR "Cardiorespiratory Fitness"[Mesh] OR "Physical Functional Performance"[Mesh] OR "Postural Balance"[Mesh] OR "Physical Endurance"[Mesh] OR "Malnutrition"[Mesh])) Publication Date ("Klotho"[Title/Abstract] OR "S-Klotho"[Title/Abstract] OR "alpha-Klotho"[Title/Abstract] OR "alpha-Klotho"[Title/Abstract] OR "Klotho Proteins"[MeSH Terms]) AND ("frail*"[Title/Abstract] OR "frailness"[Title/Abstract] OR "Muscular Weakness"[Title/Abstract] OR "biological aging"[Title/Abstract] OR "frail*"[Title/Abstract] OR "Sarcopenia"[Title/Abstract] OR "sarcopenic"[Title/Abstract] OR "debilit*"[Title/Abstract] OR "muscle"[Title/Abstract] OR "body composition*"[Title/Abstract] OR "Hand Strength"[Title/Abstract] OR "grip strength"[Title/Abstract] OR "Grasp"[Title/Abstract] OR "Aging"[Title/Abstract] OR "Aged"[Title/Abstract] OR "elderly"[Title/Abstract] OR "Older"[Title/Abstract] OR "elder"[Title/Abstract] OR "prefrailty"[Title/Abstract] OR "Walking Speed"[Title/Abstract] OR "vulnerable"[Title/Abstract] OR "vulnerability"[Title/Abstract] OR "pre-frail"[Title/Abstract] OR "multimorbid"[Title/Abstract] OR "Multimorbidity"[Title/Abstract] OR "gait disorders"[Title/Abstract] OR "aged 80"[Title/Abstract] OR "Cognition"[Title/Abstract] OR "prefrail"[Title/Abstract] OR "geriatric"[Title/Abstract] OR "geriatry"[Title/Abstract] OR "aged 60"[Title/Abstract] OR "Physical Fitness"[Title/Abstract] OR "Cardiorespiratory Fitness"[Title/Abstract] OR "functional performance"[Title/Abstract] OR "physical performance"[Title/Abstract] OR "Postural Balance"[Title/Abstract] OR "Balance"[Title/Abstract] OR "flexibility"[Title/Abstract] OR "agility"[Title/Abstract] OR "Physical Endurance"[Title/Abstract] OR "Walking Speed"[Title/Abstract] OR "geriatric syndrome"[Title/Abstract] OR ((("geriatric"[All Fields] OR "geriatrics"[MeSH Terms] OR "geriatrics"[All Fields]) AND ("disease"[MeSH Terms] OR "disease"[All Fields] OR "disorder"[All Fields] OR "disorders"[All Fields] OR "disorder s"[All Fields] OR "disordes"[All Fields])) AND "nutritional assessment"[Title/Abstract]) OR "fragility"[Title/Abstract] OR "gait speed"[Title/Abstract] OR "Walking Speed"[Title/Abstract] OR "short physical performance battery"[Title/Abstract] OR "old adult"[Title/Abstract] OR "Functionally-Impaired"[Title/Abstract] OR "Functionally-Impaired"[Title/Abstract] OR "functional impairment"[Title/Abstract] OR "function decline"[Title/Abstract] OR "decreased physiologic reserve"[Title/Abstract] OR "oldest adults"[Title/Abstract] OR "old population"[Title/Abstract] OR "oldest population"[Title/Abstract] OR "old individuals"[Title/Abstract] OR "oldest individuals"[Title/Abstract] OR "old patients"[Title/Abstract] OR "oldest patients"[Title/Abstract] OR "old persons"[Title/Abstract] OR "disability"[Title/Abstract] OR "disabled"[Title/Abstract] OR "physical disability"[Title/Abstract] OR "disabilit*"[Title/Abstract] OR "disable"[Title/Abstract] OR "activities of daily living"[Title/Abstract] OR "fat free mass"[Title/Abstract] OR "lean mass"[Title/Abstract] OR "Malnutrition"[Title/Abstract] OR "muscle function"[Title/Abstract] OR "muscular strength"[Title/Abstract] OR "Muscular Weakness"[Title/Abstract] OR "handgrip strength"[Title/Abstract] OR "lean body mass"[Title/Abstract] OR "muscular atrophy"[Title/Abstract] OR "physical function"[Title/Abstract] OR "pre-sarcopenia"[Title/Abstract] OR "presarcopenia"[Title/Abstract] OR "cachexia"[Title/Abstract] OR "cachectic"[Title/Abstract] OR "wasting"[Title/Abstract] OR "Malnutrition"[Title/Abstract] OR "malnourish"[Title/Abstract] OR "undernutrition"[Title/Abstract] OR "undernourished"[Title/Abstract] OR "Malnutrition"[Title/Abstract] OR "undernutrition"[Title/Abstract] OR "malnourishment"[Title/Abstract] OR ("Aging"[MeSH Terms] OR "Aged"[MeSH Terms] OR "Hand Strength"[MeSH Terms] OR "Body Composition"[MeSH Terms] OR "Muscles"[MeSH Terms] OR "Frailty"[MeSH Terms] OR "Sarcopenia"[MeSH Terms] OR "Muscle Weakness"[MeSH Terms] OR "muscle, skeletal"[MeSH Terms] OR "Frail Elderly"[MeSH Terms] OR "Walking Speed"[MeSH Terms] OR "Multimorbidity"[MeSH Terms] OR "Cognition"[MeSH Terms] OR "Physical Fitness"[MeSH Terms] OR "Cardiorespiratory Fitness"[MeSH Terms] OR "Physical Functional Performance"[MeSH Terms] OR "Postural Balance"[MeSH Terms] OR "Physical Endurance"[MeSH Terms] OR "Malnutrition"[MeSH Terms]))  **n =2,210**  **6** (Frail*[Title/Abstract] OR frailness[Title/Abstract] OR "Muscular Weakness"[Title/Abstract] OR "biological aging"[Title/Abstract] OR frail*[Title/Abstract] OR "sarcopenia"[Title/Abstract] OR "sarcopenic"[Title/Abstract] OR Debilit*[Title/Abstract] OR muscle[Title/Abstract] OR "Body Composition*"[Title/Abstract] OR "hand strength"[Title/Abstract] OR "grip strength"[Title/Abstract] OR Grasp[Title/Abstract] OR aging[Title/Abstract] OR aged[Title/Abstract] OR elderly[Title/Abstract] OR Older[Title/Abstract] OR elder[Title/Abstract] OR prefrailty[Title/Abstract] OR walking speed[Title/Abstract] OR vulnerable[Title/Abstract] OR vulnerability[Title/Abstract] OR pre-frail[Title/Abstract] OR multimorbid[Title/Abstract] OR multimorbidity[Title/Abstract] OR gait disorders[Title/Abstract] OR aged, 80[Title/Abstract] OR cognition[Title/Abstract] OR prefrail[Title/Abstract] OR geriatric[Title/Abstract] OR geriatry[Title/Abstract] OR aged, 60[Title/Abstract] OR Physical Fitness[Title/Abstract] OR Cardiorespiratory Fitness[Title/Abstract] OR Functional Performance[Title/Abstract] OR Physical Performance[Title/Abstract] OR Postural Balance[Title/Abstract] OR Balance[Title/Abstract] OR flexibility[Title/Abstract] OR agility[Title/Abstract] OR Physical Endurance[Title/Abstract] OR Walking Speed[Title/Abstract] OR geriatric syndrome[Title/Abstract] OR geriatric disorder nutritional assessment[Title/Abstract] OR fragility[Title/Abstract] OR Gait Speed[Title/Abstract] OR Walking speed[Title/Abstract] OR Short Physical Performance Battery[Title/Abstract] OR old adult[Title/Abstract] OR Functionally-Impaired[Title/Abstract] OR Functionally Impaired[Title/Abstract] OR functional impairment[Title/Abstract] OR function decline[Title/Abstract] OR Decreased Physiologic Reserve[Title/Abstract] OR oldest adults[Title/Abstract] OR old population[Title/Abstract] OR oldest population[Title/Abstract] OR old individuals[Title/Abstract] OR oldest individuals[Title/Abstract] OR old patients[Title/Abstract] OR oldest patients[Title/Abstract] OR old persons[Title/Abstract] OR disability[Title/Abstract] OR disabled[Title/Abstract] OR physical disability[Title/Abstract] OR disabilit*[Title/Abstract] OR disable[Title/Abstract] OR activities of daily living[Title/Abstract] OR fat free mass[Title/Abstract] OR lean mass[Title/Abstract] OR Malnutrition[Title/Abstract] OR muscle function[Title/Abstract] OR muscular strength[Title/Abstract] OR muscular weakness[Title/Abstract] OR "handgrip strength"[Title/Abstract] OR lean body mass[Title/Abstract] OR muscular atrophy[Title/Abstract] OR physical function[Title/Abstract] OR pre-sarcopenia[Title/Abstract] OR presarcopenia[Title/Abstract] OR cachexia[Title/Abstract] OR cachectic[Title/Abstract] OR wasting[Title/Abstract] OR malnutrition[Title/Abstract] OR malnourish[Title/Abstract] OR undernutrition[Title/Abstract] OR undernourished[Title/Abstract] OR malnutrition[Title/Abstract] OR undernutrition[Title/Abstract] OR malnourishment[Title/Abstract]) OR ("Aging"[Mesh] OR "Aged"[Mesh] OR "Hand Strength"[Mesh] OR "Body Composition"[Mesh] OR "Muscles"[Mesh] OR "Frailty"[Mesh] OR "Sarcopenia"[Mesh] OR "Muscle Weakness"[Mesh] OR "Muscle, Skeletal"[Mesh] OR "Frail Elderly"[Mesh] OR "Walking Speed"[Mesh] OR "Multimorbidity"[Mesh] OR "Cognition"[Mesh] OR "Physical Fitness"[Mesh] OR "Cardiorespiratory Fitness"[Mesh] OR "Physical Functional Performance"[Mesh] OR "Postural Balance"[Mesh] OR "Physical Endurance"[Mesh] OR "Malnutrition"[Mesh]) Publication Date "frail*"[Title/Abstract] OR "frailness"[Title/Abstract] OR "Muscular Weakness"[Title/Abstract] OR "biological aging"[Title/Abstract] OR "frail*"[Title/Abstract] OR "Sarcopenia"[Title/Abstract] OR "sarcopenic"[Title/Abstract] OR "debilit*"[Title/Abstract] OR "muscle"[Title/Abstract] OR "body composition*"[Title/Abstract] OR "Hand Strength"[Title/Abstract] OR "grip strength"[Title/Abstract] OR "Grasp"[Title/Abstract] OR "Aging"[Title/Abstract] OR "Aged"[Title/Abstract] OR "elderly"[Title/Abstract] OR "Older"[Title/Abstract] OR "elder"[Title/Abstract] OR "prefrailty"[Title/Abstract] OR "Walking Speed"[Title/Abstract] OR "vulnerable"[Title/Abstract] OR "vulnerability"[Title/Abstract] OR "pre-frail"[Title/Abstract] OR "multimorbid"[Title/Abstract] OR "Multimorbidity"[Title/Abstract] OR "gait disorders"[Title/Abstract] OR "aged 80"[Title/Abstract] OR "Cognition"[Title/Abstract] OR "prefrail"[Title/Abstract] OR "geriatric"[Title/Abstract] OR "geriatry"[Title/Abstract] OR "aged 60"[Title/Abstract] OR "Physical Fitness"[Title/Abstract] OR "Cardiorespiratory Fitness"[Title/Abstract] OR "functional performance"[Title/Abstract] OR "physical performance"[Title/Abstract] OR "Postural Balance"[Title/Abstract] OR "Balance"[Title/Abstract] OR "flexibility"[Title/Abstract] OR "agility"[Title/Abstract] OR "Physical Endurance"[Title/Abstract] OR "Walking Speed"[Title/Abstract] OR "geriatric syndrome"[Title/Abstract] OR ((("geriatric"[All Fields] OR "geriatrics"[MeSH Terms] OR "geriatrics"[All Fields]) AND ("disease"[MeSH Terms] OR "disease"[All Fields] OR "disorder"[All Fields] OR "disorders"[All Fields] OR "disorder s"[All Fields] OR "disordes"[All Fields])) AND "nutritional assessment"[Title/Abstract]) OR "fragility"[Title/Abstract] OR "gait speed"[Title/Abstract] OR "Walking Speed"[Title/Abstract] OR "short physical performance battery"[Title/Abstract] OR "old adult"[Title/Abstract] OR "Functionally-Impaired"[Title/Abstract] OR "Functionally-Impaired"[Title/Abstract] OR "functional impairment"[Title/Abstract] OR "function decline"[Title/Abstract] OR "decreased physiologic reserve"[Title/Abstract] OR "oldest adults"[Title/Abstract] OR "old population"[Title/Abstract] OR "oldest population"[Title/Abstract] OR "old individuals"[Title/Abstract] OR "oldest individuals"[Title/Abstract] OR "old patients"[Title/Abstract] OR "oldest patients"[Title/Abstract] OR "old persons"[Title/Abstract] OR "disability"[Title/Abstract] OR "disabled"[Title/Abstract] OR "physical disability"[Title/Abstract] OR "disabilit*"[Title/Abstract] OR "disable"[Title/Abstract] OR "activities of daily living"[Title/Abstract] OR "fat free mass"[Title/Abstract] OR "lean mass"[Title/Abstract] OR "Malnutrition"[Title/Abstract] OR "muscle function"[Title/Abstract] OR "muscular strength"[Title/Abstract] OR "Muscular Weakness"[Title/Abstract] OR "handgrip strength"[Title/Abstract] OR "lean body mass"[Title/Abstract] OR "muscular atrophy"[Title/Abstract] OR "physical function"[Title/Abstract] OR "pre-sarcopenia"[Title/Abstract] OR "presarcopenia"[Title/Abstract] OR "cachexia"[Title/Abstract] OR "cachectic"[Title/Abstract] OR "wasting"[Title/Abstract] OR "Malnutrition"[Title/Abstract] OR "malnourish"[Title/Abstract] OR "undernutrition"[Title/Abstract] OR "undernourished"[Title/Abstract] OR "Malnutrition"[Title/Abstract] OR "undernutrition"[Title/Abstract] OR "malnourishment"[Title/Abstract] OR ("Aging"[MeSH Terms] OR "Aged"[MeSH Terms] OR "Hand Strength"[MeSH Terms] OR "Body Composition"[MeSH Terms] OR "Muscles"[MeSH Terms] OR "Frailty"[MeSH Terms] OR "Sarcopenia"[MeSH Terms] OR "Muscle Weakness"[MeSH Terms] OR "muscle, skeletal"[MeSH Terms] OR "Frail Elderly"[MeSH Terms] OR "Walking Speed"[MeSH Terms] OR "Multimorbidity"[MeSH Terms] OR "Cognition"[MeSH Terms] OR "Physical Fitness"[MeSH Terms] OR "Cardiorespiratory Fitness"[MeSH Terms] OR "Physical Functional Performance"[MeSH Terms] OR "Postural Balance"[MeSH Terms] OR "Physical Endurance"[MeSH Terms] OR "Malnutrition"[MeSH Terms])  **n =6,984,293**  **5** "Aging"[Mesh] OR "Aged"[Mesh] OR "Hand Strength"[Mesh] OR "Body Composition"[Mesh] OR "Muscles"[Mesh] OR "Frailty"[Mesh] OR "Sarcopenia"[Mesh] OR "Muscle Weakness"[Mesh] OR "Muscle, Skeletal"[Mesh] OR "Frail Elderly"[Mesh] OR "Walking Speed"[Mesh] OR "Multimorbidity"[Mesh] OR "Cognition"[Mesh] OR "Physical Fitness"[Mesh] OR "Cardiorespiratory Fitness"[Mesh] OR "Physical Functional Performance"[Mesh] OR "Postural Balance"[Mesh] OR "Physical Endurance"[Mesh] OR "Malnutrition"[Mesh] Publication Date "Aging"[MeSH Terms] OR "Aged"[MeSH Terms] OR "Hand Strength"[MeSH Terms] OR "Body Composition"[MeSH Terms] OR "Muscles"[MeSH Terms] OR "Frailty"[MeSH Terms] OR "Sarcopenia"[MeSH Terms] OR "Muscle Weakness"[MeSH Terms] OR "muscle, skeletal"[MeSH Terms] OR "Frail Elderly"[MeSH Terms] OR "Walking Speed"[MeSH Terms] OR "Multimorbidity"[MeSH Terms] OR "Cognition"[MeSH Terms] OR "Physical Fitness"[MeSH Terms] OR "Cardiorespiratory Fitness"[MeSH Terms] OR "Physical Functional Performance"[MeSH Terms] OR "Postural Balance"[MeSH Terms] OR "Physical Endurance"[MeSH Terms] OR "Malnutrition"[MeSH Terms]  **n =4,905,111**  **4** Frail*[Title/Abstract] OR frailness[Title/Abstract] OR "Muscular Weakness"[Title/Abstract] OR "biological aging"[Title/Abstract] OR frail*[Title/Abstract] OR "sarcopenia"[Title/Abstract] OR "sarcopenic"[Title/Abstract] OR Debilit*[Title/Abstract] OR muscle[Title/Abstract] OR "Body Composition*"[Title/Abstract] OR "hand strength"[Title/Abstract] OR "grip strength"[Title/Abstract] OR Grasp[Title/Abstract] OR aging[Title/Abstract] OR aged[Title/Abstract] OR elderly[Title/Abstract] OR Older[Title/Abstract] OR elder[Title/Abstract] OR prefrailty[Title/Abstract] OR walking speed[Title/Abstract] OR vulnerable[Title/Abstract] OR vulnerability[Title/Abstract] OR pre-frail[Title/Abstract] OR multimorbid[Title/Abstract] OR multimorbidity[Title/Abstract] OR gait disorders[Title/Abstract] OR aged, 80[Title/Abstract] OR cognition[Title/Abstract] OR prefrail[Title/Abstract] OR geriatric[Title/Abstract] OR geriatry[Title/Abstract] OR aged, 60[Title/Abstract] OR Physical Fitness[Title/Abstract] OR Cardiorespiratory Fitness[Title/Abstract] OR Functional Performance[Title/Abstract] OR Physical Performance[Title/Abstract] OR Postural Balance[Title/Abstract] OR Balance[Title/Abstract] OR flexibility[Title/Abstract] OR agility[Title/Abstract] OR Physical Endurance[Title/Abstract] OR Walking Speed[Title/Abstract] OR geriatric syndrome[Title/Abstract] OR geriatric disorder nutritional assessment[Title/Abstract] OR fragility[Title/Abstract] OR Gait Speed[Title/Abstract] OR Walking speed[Title/Abstract] OR Short Physical Performance Battery[Title/Abstract] OR old adult[Title/Abstract] OR Functionally-Impaired[Title/Abstract] OR Functionally Impaired[Title/Abstract] OR functional impairment[Title/Abstract] OR function decline[Title/Abstract] OR Decreased Physiologic Reserve[Title/Abstract] OR oldest adults[Title/Abstract] OR old population[Title/Abstract] OR oldest population[Title/Abstract] OR old individuals[Title/Abstract] OR oldest individuals[Title/Abstract] OR old patients[Title/Abstract] OR oldest patients[Title/Abstract] OR old persons[Title/Abstract] OR disability[Title/Abstract] OR disabled[Title/Abstract] OR physical disability[Title/Abstract] OR disabilit*[Title/Abstract] OR disable[Title/Abstract] OR activities of daily living[Title/Abstract] OR fat free mass[Title/Abstract] OR lean mass[Title/Abstract] OR Malnutrition[Title/Abstract] OR muscle function[Title/Abstract] OR muscular strength[Title/Abstract] OR muscular weakness[Title/Abstract] OR "handgrip strength"[Title/Abstract] OR lean body mass[Title/Abstract] OR muscular atrophy[Title/Abstract] OR physical function[Title/Abstract] OR pre-sarcopenia[Title/Abstract] OR presarcopenia[Title/Abstract] OR cachexia[Title/Abstract] OR cachectic[Title/Abstract] OR wasting[Title/Abstract] OR malnutrition[Title/Abstract] OR malnourish[Title/Abstract] OR undernutrition[Title/Abstract] OR undernourished[Title/Abstract] OR malnutrition[Title/Abstract] OR undernutrition[Title/Abstract] OR malnourishment[Title/Abstract] Publication Date "frail*"[Title/Abstract] OR "frailness"[Title/Abstract] OR "Muscular Weakness"[Title/Abstract] OR "biological aging"[Title/Abstract] OR "frail*"[Title/Abstract] OR "sarcopenia"[Title/Abstract] OR "sarcopenic"[Title/Abstract] OR "debilit*"[Title/Abstract] OR "muscle"[Title/Abstract] OR "body composition*"[Title/Abstract] OR "hand strength"[Title/Abstract] OR "grip strength"[Title/Abstract] OR "Grasp"[Title/Abstract] OR "aging"[Title/Abstract] OR "aged"[Title/Abstract] OR "elderly"[Title/Abstract] OR "Older"[Title/Abstract] OR "elder"[Title/Abstract] OR "prefrailty"[Title/Abstract] OR "walking speed"[Title/Abstract] OR "vulnerable"[Title/Abstract] OR "vulnerability"[Title/Abstract] OR "pre-frail"[Title/Abstract] OR "multimorbid"[Title/Abstract] OR "multimorbidity"[Title/Abstract] OR "gait disorders"[Title/Abstract] OR "aged 80"[Title/Abstract] OR "cognition"[Title/Abstract] OR "prefrail"[Title/Abstract] OR "geriatric"[Title/Abstract] OR "geriatry"[Title/Abstract] OR "aged 60"[Title/Abstract] OR "physical fitness"[Title/Abstract] OR "cardiorespiratory fitness"[Title/Abstract] OR "functional performance"[Title/Abstract] OR "physical performance"[Title/Abstract] OR "postural balance"[Title/Abstract] OR "Balance"[Title/Abstract] OR "flexibility"[Title/Abstract] OR "agility"[Title/Abstract] OR "physical endurance"[Title/Abstract] OR "walking speed"[Title/Abstract] OR "geriatric syndrome"[Title/Abstract] OR ((("geriatric"[All Fields] OR "geriatrics"[MeSH Terms] OR "geriatrics"[All Fields]) AND ("disease"[MeSH Terms] OR "disease"[All Fields] OR "disorder"[All Fields] OR "disorders"[All Fields] OR "disorder s"[All Fields] OR "disordes"[All Fields])) AND "nutritional assessment"[Title/Abstract]) OR "fragility"[Title/Abstract] OR "gait speed"[Title/Abstract] OR "walking speed"[Title/Abstract] OR "short physical performance battery"[Title/Abstract] OR "old adult"[Title/Abstract] OR "Functionally-Impaired"[Title/Abstract] OR "Functionally-Impaired"[Title/Abstract] OR "functional impairment"[Title/Abstract] OR "function decline"[Title/Abstract] OR "decreased physiologic reserve"[Title/Abstract] OR "oldest adults"[Title/Abstract] OR "old population"[Title/Abstract] OR "oldest population"[Title/Abstract] OR "old individuals"[Title/Abstract] OR "oldest individuals"[Title/Abstract] OR "old patients"[Title/Abstract] OR "oldest patients"[Title/Abstract] OR "old persons"[Title/Abstract] OR "disability"[Title/Abstract] OR "disabled"[Title/Abstract] OR "physical disability"[Title/Abstract] OR "disabilit*"[Title/Abstract] OR "disable"[Title/Abstract] OR "activities of daily living"[Title/Abstract] OR "fat free mass"[Title/Abstract] OR "lean mass"[Title/Abstract] OR "Malnutrition"[Title/Abstract] OR "muscle function"[Title/Abstract] OR "muscular strength"[Title/Abstract] OR "Muscular Weakness"[Title/Abstract] OR "handgrip strength"[Title/Abstract] OR "lean body mass"[Title/Abstract] OR "muscular atrophy"[Title/Abstract] OR "physical function"[Title/Abstract] OR "pre-sarcopenia"[Title/Abstract] OR "presarcopenia"[Title/Abstract] OR "cachexia"[Title/Abstract] OR "cachectic"[Title/Abstract] OR "wasting"[Title/Abstract] OR "Malnutrition"[Title/Abstract] OR "malnourish"[Title/Abstract] OR "undernutrition"[Title/Abstract] OR "undernourished"[Title/Abstract] OR "Malnutrition"[Title/Abstract] OR "undernutrition"[Title/Abstract] OR "malnourishment"[Title/Abstract]  **n = 3,513,152**  **3** (Klotho[Title/Abstract] OR S-Klotho[Title/Abstract] OR α-Klotho[Title/Abstract] OR alpha-Klotho[Title/Abstract]) OR ("Klotho Proteins"[Mesh]) Publication Date "Klotho"[Title/Abstract] OR "S-Klotho"[Title/Abstract] OR "alpha-Klotho"[Title/Abstract] OR "alpha-Klotho"[Title/Abstract] OR "Klotho Proteins"[MeSH Terms]  **n = 3,999**  **2** "Klotho Proteins"[Mesh] Publication Date "Klotho Proteins"[MeSH Terms]  **n = 2,224**  **1** Klotho[Title/Abstract] OR S-Klotho[Title/Abstract] OR α-Klotho[Title/Abstract] OR alpha-Klotho[Title/Abstract] Publication Date "Klotho"[Title/Abstract] OR "S-Klotho"[Title/Abstract] OR "alpha-Klotho"[Title/Abstract] OR "alpha-Klotho"[Title/Abstract]  **3,785** |
| **Scopus (n=1835)** |
| TITLE-ABS ( klotho OR s-klotho OR &#945;-klotho OR alpha-klotho ) AND TITLE-ABS ( frail* OR frailness OR "Muscular Weakness" OR "biological aging" OR frail* OR "sarcopenia" OR "sarcopenic" OR debilit* OR muscle OR "Body Composition*" OR "hand strength" OR "grip strength" OR grasp OR aging OR aged OR elderly OR older OR elder OR prefrailty OR "walking speed" OR vulnerable OR vulnerability OR pre-frail OR multimorbid OR multimorbidity OR "gait disorders" OR "aged, 80" OR cognition OR prefrail OR geriatric OR geriatry OR "aged, 60" OR "Physical Fitness" OR "Cardiorespiratory Fitness" OR "Functional Performance" OR "Physical Performance" OR "Postural Balance" OR balance OR flexibility OR agility OR "Physical Endurance" OR "Walking Speed" OR "geriatric syndrome" OR "geriatric disorder nutritional assessment" OR fragility OR "Gait Speed" OR "Walking speed" OR "Short Physical Performance Battery" OR "old adult" OR "Functionally-Impaired" OR "Functionally Impaired" OR "functional impairment" OR "function decline" OR "Decreased Physiologic Reserve" OR "oldest adults" OR "old population" OR "oldest population" OR "old individuals" OR "oldest individuals" OR "old patients" OR "oldest patients" OR "old persons" OR disability OR disabled OR "physical disability" OR disabilit* OR disable OR "activities of daily living" OR "fat free mass" OR "lean mass" OR malnutrition OR "muscle function" OR "muscular strength" OR "muscular weakness" OR "handgrip strength" OR "lean body mass" OR "muscular atrophy" OR "physical function" OR "pre-sarcopenia" OR presarcopenia OR cachexia OR cachectic OR wasting OR malnutrition OR malnourish OR undernutrition OR undernourished OR malnutrition OR undernutrition OR malnourishment )  **n= 1835** |
| **Web of Science (n=2173)** |
| **1:** (TI=( klotho OR s-klotho OR α-klotho OR alpha-klotho)) OR AB=( klotho OR s-klotho OR α-klotho OR alpha-klotho) Date Run: Mon Dec 30 2024 16:17:09 GMT+0300 (GMT+03:00)  **Results: 4452**  **2:** (TI=(frail* OR frailness OR "Muscular Weakness" OR "biological aging" OR frail* OR "sarcopenia" OR "sarcopenic" OR debilit* OR muscle OR "Body Composition*" OR "hand strength" OR "grip strength" OR grasp OR aging OR aged OR elderly OR older OR elder OR prefrailty OR "walking speed" OR vulnerable OR vulnerability OR pre-frail OR multimorbid OR multimorbidity OR "gait disorders" OR "aged, 80" OR cognition OR prefrail OR geriatric OR geriatry OR "aged, 60" OR "Physical Fitness" OR "Cardiorespiratory Fitness" OR "Functional Performance" OR "Physical Performance" OR "Postural Balance" OR balance OR flexibility OR agility OR "Physical Endurance" OR "Walking Speed" OR "geriatric syndrome" OR "geriatric disorder nutritional assessment" OR fragility OR "Gait Speed" OR "Walking speed" OR "Short Physical Performance Battery" OR "old adult" OR "Functionally-Impaired" OR "Functionally Impaired" OR "functional impairment" OR "function decline" OR "Decreased Physiologic Reserve" OR "oldest adults" OR "old population" OR "oldest population" OR "old individuals" OR "oldest individuals" OR "old patients" OR "oldest patients" OR "old persons" OR disability OR disabled OR "physical disability" OR disabilit* OR disable OR "activities of daily living" OR "fat free mass" OR "lean mass" OR malnutrition OR "muscle function" OR "muscular strength" OR "muscular weakness" OR "handgrip strength" OR "lean body mass" OR "muscular atrophy" OR "physical function" OR "pre-sarcopenia" OR presarcopenia OR cachexia OR cachectic OR wasting OR malnutrition OR malnourish OR undernutrition OR undernourished OR malnutrition OR undernutrition OR malnourishment)) OR AB=(frail* OR frailness OR "Muscular Weakness" OR "biological aging" OR frail* OR "sarcopenia" OR "sarcopenic" OR debilit* OR muscle OR "Body Composition*" OR "hand strength" OR "grip strength" OR grasp OR aging OR aged OR elderly OR older OR elder OR prefrailty OR "walking speed" OR vulnerable OR vulnerability OR pre-frail OR multimorbid OR multimorbidity OR "gait disorders" OR "aged, 80" OR cognition OR prefrail OR geriatric OR geriatry OR "aged, 60" OR "Physical Fitness" OR "Cardiorespiratory Fitness" OR "Functional Performance" OR "Physical Performance" OR "Postural Balance" OR balance OR flexibility OR agility OR "Physical Endurance" OR "Walking Speed" OR "geriatric syndrome" OR "geriatric disorder nutritional assessment" OR fragility OR "Gait Speed" OR "Walking speed" OR "Short Physical Performance Battery" OR "old adult" OR "Functionally-Impaired" OR "Functionally Impaired" OR "functional impairment" OR "function decline" OR "Decreased Physiologic Reserve" OR "oldest adults" OR "old population" OR "oldest population" OR "old individuals" OR "oldest individuals" OR "old patients" OR "oldest patients" OR "old persons" OR disability OR disabled OR "physical disability" OR disabilit* OR disable OR "activities of daily living" OR "fat free mass" OR "lean mass" OR malnutrition OR "muscle function" OR "muscular strength" OR "muscular weakness" OR "handgrip strength" OR "lean body mass" OR "muscular atrophy" OR "physical function" OR "pre-sarcopenia" OR presarcopenia OR cachexia OR cachectic OR wasting OR malnutrition OR malnourish OR undernutrition OR undernourished OR malnutrition OR undernutrition OR malnourishment) Date Run: Mon Dec 30 2024 16:17:33 GMT+0300 (GMT+03:00)  **Results: 9517407**  **3:** #2 AND #1  Date Run: Mon Dec 30 2024 16:17:45 GMT+0300 (GMT+03:00)  **Results: 2173** |
| **Cochrane Library (n=173)** |
| Klotho OR S-Klotho OR α-Klotho OR alpha-Klotho in Title Abstract Keyword AND Frail* OR frailness OR "Muscular Weakness" OR "biological aging" OR frail* OR "sarcopenia" OR "sarcopenic" OR Debilit* OR muscle OR Body Composition* OR "hand strength" OR "grip strength" OR Grasp OR aging OR aged OR elderly OR Older OR elder OR prefrailty OR "walking speed" OR vulnerable OR vulnerability OR pre-frail OR multimorbid OR multimorbidity OR "gait disorders" OR "aged, 80" OR cognition OR prefrail OR geriatric OR geriatry OR "aged, 60" OR "Physical Fitness" OR "Cardiorespiratory Fitness" OR "Functional Performance" OR "Physical Performance" OR "Postural Balance" OR Balance OR flexibility OR agility OR "Physical Endurance" OR "Walking Speed" OR "geriatric syndrome" OR "geriatric disorder nutritional assessment" OR fragility OR "Gait Speed" OR "Walking speed" OR "Short Physical Performance Battery" OR "old adult" OR "Functionally-Impaired" OR "Functionally Impaired" OR "functional impairment" OR "function decline" OR "Decreased Physiologic Reserve" OR "oldest adults" OR "old population" OR "oldest population" OR "old individuals" OR "oldest individuals" OR "old patients" OR "oldest patients" OR "old persons" OR disability OR disabled OR "physical disability" OR disabilit* OR disable OR "activities of daily living" OR "fat free mass" OR "lean mass" OR Malnutrition OR "muscle function" OR "muscular strength" OR "muscular weakness" OR "handgrip strength" OR "lean body mass" OR "muscular atrophy" OR "physical function" OR "pre-sarcopenia" OR presarcopenia OR cachexia OR cachectic OR wasting OR malnutrition OR malnourish OR undernutrition OR undernourished OR malnutrition OR undernutrition OR malnourishment in Title Abstract Keyword - (Word variations have been searched)  **n=173** |
| **Ovid MEDLINE (n=1643)** |
| **Ovid MEDLINE(R) and Epub Ahead of Print, In-Process, In-Data-Review & Other Non-Indexed Citations, Daily and Versions <1946 to December 27, 2024>**  **1** (klotho or "s-klotho" or "α-klotho" or "alpha-klotho").ti. or (klotho or "s-klotho" or "α-klotho" or "alpha-klotho").ab. 3668  2 (frail* or frailness or "Muscular Weakness" or "biological aging" or frail* or "sarcopenia" or "sarcopenic" or debilit* or muscle or "Body Composition*" or "hand strength" or "grip strength" or grasp or aging or aged or elderly or older or elder or prefrailty or "walking speed" or vulnerable or vulnerability or pre-frail or multimorbid or multimorbidity or "gait disorders" or "aged, 80" or cognition or prefrail or geriatric or geriatry or "aged, 60" or "Physical Fitness" or "Cardiorespiratory Fitness" or "Functional Performance" or "Physical Performance" or "Postural Balance" or balance or flexibility or agility or "Physical Endurance" or "Walking Speed" or "geriatric syndrome" or "geriatric disorder nutritional assessment" or fragility or "Gait Speed" or "Walking speed" or "Short Physical Performance Battery" or "old adult" or "Functionally-Impaired" or "Functionally Impaired" or "functional impairment" or "function decline" or "Decreased Physiologic Reserve" or "oldest adults" or "old population" or "oldest population" or "old individuals" or "oldest individuals" or "old patients" or "oldest patients" or "old persons" or disability or disabled or "physical disability" or disabilit* or disable or "activities of daily living" or "fat free mass" or "lean mass" or malnutrition or "muscle function" or "muscular strength" or "muscular weakness" or "handgrip strength" or "lean body mass" or "muscular atrophy" or "physical function" or "pre-sarcopenia" or presarcopenia or cachexia or cachectic or wasting or malnutrition or malnourish or undernutrition or undernourished or malnutrition or undernutrition or malnourishment).ti. or (frail* or frailness or "Muscular Weakness" or "biological aging" or frail* or "sarcopenia" or "sarcopenic" or debilit* or muscle or "Body Composition*" or "hand strength" or "grip strength" or grasp or aging or aged or elderly or older or elder or prefrailty or "walking speed" or vulnerable or vulnerability or pre-frail or multimorbid or multimorbidity or "gait disorders" or "aged, 80" or cognition or prefrail or geriatric or geriatry or "aged, 60" or "Physical Fitness" or "Cardiorespiratory Fitness" or "Functional Performance" or "Physical Performance" or "Postural Balance" or balance or flexibility or agility or "Physical Endurance" or "Walking Speed" or "geriatric syndrome" or "geriatric disorder nutritional assessment" or fragility or "Gait Speed" or "Walking speed" or "Short Physical Performance Battery" or "old adult" or "Functionally-Impaired" or "Functionally Impaired" or "functional impairment" or "function decline" or "Decreased Physiologic Reserve" or "oldest adults" or "old population" or "oldest population" or "old individuals" or "oldest individuals" or "old patients" or "oldest patients" or "old persons" or disability or disabled or "physical disability" or disabilit* or disable or "activities of daily living" or "fat free mass" or "lean mass" or malnutrition or "muscle function" or "muscular strength" or "muscular weakness" or "handgrip strength" or "lean body mass" or "muscular atrophy" or "physical function" or "pre-sarcopenia" or presarcopenia or cachexia or cachectic or wasting or malnutrition or malnourish or undernutrition or undernourished or malnutrition or undernutrition or malnourishment).ab. 3448716  3 1 and 2  **n= 1643** |

**Supplementary Table S2**. Quality assessment of cross-sectional studies with JBI critical appraisal tool

| **No** | **Study Name** | **Q1** | **Q2** | **Q3** | **Q4** | **Q5** | **Q6** | **Q7** | **Q8** | **%Yes** | **Risk** |
| --- | --- | --- | --- | --- | --- | --- | --- | --- | --- | --- | --- |
| 1 | Aczel (2023) | No | No | Yes | Yes | Yes | No | Yes | Yes | 62.5 | Moderate |
| 2 | Amaro-Gahete (2019)[1] | Yes | Yes | Yes | Yes | Yes | Yes | Yes | Yes | 100 | Low |
| 3 | Amaro-Gahete (2019)[2] | Yes | Yes | Yes | Yes | Yes | Yes | Yes | Yes | 100 | Low |
| 4 | Arroyo (2023) | Yes | Yes | Yes | Yes | Yes | No | Yes | Yes | 87.5 | Low |
| 5 | Baldan (2015) | Yes | Yes | Yes | Yes | Yes | No | Yes | Yes | 87.5 | Low |
| 6 | Barbosa (2017) | Yes | Yes | Yes | Yes | Yes | No | Yes | Yes | 87.5 | Low |
| 7 | Brombo (2018) | Yes | Yes | Yes | Yes | Yes | Yes | Yes | Yes | 100 | Low |
| 8 | Chen (2024) | Yes | Yes | Yes | Yes | Yes | Yes | Yes | Yes | 100 | Low |
| 9 | Crasto (2012) | Yes | Yes | Yes | Yes | Yes | Yes | Yes | Yes | 100 | Low |
| 10 | Emir (2023) | Yes | Yes | Yes | Yes | Yes | No | Yes | Yes | 87.5 | Low |
| 11 | Ferreira (2021) | Yes | Yes | Yes | Yes | Yes | No | Yes | Yes | 87.5 | Low |
| 12 | Fukasawa (2014) | Yes | Yes | Yes | Yes | Yes | Yes | Yes | Yes | 100 | Low |
| 13 | Gao (2021) | Yes | Yes | Yes | Yes | Yes | No | Yes | Yes | 87.5 | Low |
| 14 | Ge (2024) | Yes | Yes | Yes | Yes | Yes | Yes | Yes | Yes | 100 | Low |
| 15 | Girginer (2019) | Yes | Yes | No | Yes | Yes | No | Yes | Yes | 75 | Moderate |
| 16 | Guan (2023) | Yes | Yes | Yes | Yes | Yes | Yes | Yes | Yes | 100 | Low |
| 17 | Guo (2017) | Yes | Yes | Yes | Yes | Yes | Yes | Yes | Yes | 100 | Low |
| 18 | Han (2018) | Yes | Yes | Yes | Yes | Yes | Yes | Yes | Yes | 100 | Low |
| 19 | Huang (2018) | Yes | Yes | Yes | Yes | Yes | No | Yes | Yes | 87.5 | Low |
| 20 | Jiang (2023)[3] | Yes | Yes | Yes | Yes | Yes | Yes | Yes | Yes | 100 | Low |
| 21 | Jiang (2023)[4] | Yes | Yes | Yes | Yes | Yes | Yes | Yes | Yes | 100 | Low |
| 22 | Kato (2024) | Yes | Yes | Yes | Yes | Yes | Yes | Yes | Yes | 100 | Low |
| 23 | Katsaounis (2022) | Yes | Yes | Yes | Yes | Yes | No | Yes | Yes | 87.5 | Low |
| 24 | Kılıçarslan (2022) | Yes | Yes | Yes | Yes | Yes | No | Yes | Yes | 87.5 | Low |
| 25 | Koyama (2015) | Yes | Yes | Yes | Yes | Yes | No | Yes | Yes | 87.5 | Low |
| 26 | Kundu (2022) | Yes | Yes | Yes | Yes | Yes | No | Yes | Yes | 87.5 | Low |
| 27 | Kureya (2016) | Yes | Yes | Yes | Yes | No | No | Yes | Yes | 75 | Moderate |
| 28 | Kuzmova (2021) | Yes | Yes | Yes | Yes | Yes | No | Yes | No | 75 | Moderate |
| 29 | Li (2023) | Yes | Yes | Yes | Yes | Yes | Yes | Yes | Yes | 100 | Low |
| 30 | Lima (2019) | Yes | Yes | Yes | Yes | Yes | No | Yes | Yes | 87.5 | Low |
| 31 | Linghui (2023) | Yes | Yes | Yes | Yes | Yes | Yes | Yes | Yes | 100 | Low |
| 32 | Marchelek-Mysliwiec (2018) | Yes | No | Yes | Yes | Yes | No | Yes | Yes | 75 | Moderate |
| 33 | Martín-González et al. (2023) | Yes | No | Yes | Yes | Yes | Yes | Yes | Yes | 87.5 | Low |
| 34 | Matei (2022) | Yes | Yes | Yes | Yes | Yes | Yes | Yes | Yes | 100 | Low |
| 35 | Mostafidi (2016) | Yes | No | No | No | No | No | Yes | Yes | 37.5 | High |
| 36 | Polat (2020) | Yes | Yes | Yes | Yes | Yes | Yes | Yes | Yes | 100 | Low |
| 37 | Pedersen (2013) | Yes | Yes | Yes | Yes | Yes | Yes | Yes | Yes | 100 | Low |
| 38 | Prather (2015) | Yes | Yes | Yes | Yes | Yes | Yes | Yes | Yes | 100 | Low |
| 39 | Saghiv (2017) | Yes | No | No | Yes | No | No | Yes | Yes | 50 | High |
| 40 | Sagi (2020) | Yes | No | Yes | Yes | No | Yes | Yes | Yes | 75 | Moderate |
| 41 | Salamanna (2024) | No | No | Yes | Yes | Yes | No | Yes | Yes | 62.5 | Moderate |
| 42 | Santos Rosa (2020) | Yes | Yes | Yes | Yes | No | No | Yes | Yes | 75 | Moderate |
| 43 | Sanz (2021) | Yes | Yes | Yes | Yes | Yes | No | Yes | Yes | 87.5 | Low |
| 44 | Sawada (2024) | Yes | Yes | No | No | Yes | Yes | Yes | Yes | 75 | Moderate |
| 45 | Teng Lee (2023) | Yes | Yes | Yes | Yes | Yes | Yes | Yes | Yes | 100 | Low |
| 46 | Wang (2024) | Yes | Yes | Yes | Yes | Yes | No | Yes | Yes | 87.5 | Low |
| 47 | Wu (2023) | Yes | Yes | Yes | Yes | Yes | Yes | Yes | Yes | 100 | Low |
| 48 | Wu (2024) | Yes | Yes | Yes | Yes | Yes | Yes | Yes | Yes | 100 | Low |
| 49 | Xie (2024) | Yes | Yes | Yes | Yes | Yes | Yes | Yes | Yes | 100 | Low |
| 50 | Xiong (2020) | Yes | Yes | Yes | Yes | Yes | Yes | Yes | Yes | 100 | Low |
| 51 | Yalçın (2025) | Yes | Yes | Yes | Yes | Yes | Yes | No | Yes | 87.5 | Low |
| 52 | Yokoyama (2017) | Yes | Yes | Yes | Yes | Yes | Yes | Yes | Yes | 100 | Low |
| 53 | Yuan (2024) | Yes | Yes | Yes | Yes | Yes | Yes | Yes | Yes | 100 | Low |
| 54 | Żelaźniewicz (2022) | Yes | No | Yes | Yes | Yes | No | Yes | Yes | 75 | Moderate |
| 55 | Zhang (2022) | Yes | Yes | No | Yes | Yes | Yes | Yes | Yes | 87.5 | Low |
| 56 | Zhang (2023)[5] | Yes | Yes | Yes | Yes | Yes | Yes | Yes | Yes | 100 | Low |
| 57 | Zhang (2023)[6] | Yes | Yes | Yes | Yes | Yes | No | Yes | Yes | 87.5 | Low |
| 58 | Zhang (2023)[7] | Yes | Yes | Yes | Yes | Yes | Yes | Yes | Yes | 100 | Low |
| 59 | Zheng (2018) | Yes | Yes | Yes | Yes | Yes | Yes | Yes | Yes | 100 | Low |
| 60 | Zhu (2018) | Yes | Yes | Yes | Yes | Yes | Yes | Yes | Yes | 100 | Low |

**Supplementary Table S3.** Quality assessment of cohort studies with Newcastle – Ottawa Quality Assessment Scale (NOS)

|  |  | **Selection** | | | | **Comparability** | **Outcome** | | |  |  |
| --- | --- | --- | --- | --- | --- | --- | --- | --- | --- | --- | --- |
| No | Study | Representativeness of exposed cohort | Selection of non-exposed cohort | Ascertainment of exposure | Demonstration that outcome was not present at start | Study controlled for key confounders | Assessment of outcome | Sufficient follow-up duration | Adequacy of follow-up | Total Stars | Overall Bias |
| 1 | Branco (2023) |  | ⭐ | ⭐ | ⭐ | ⭐⭐ | ⭐ | ⭐ | ⭐ | 8 | Low |
| 2 | Chuang (2023) | ⭐ | ⭐ | ⭐ | ⭐ | ⭐ | ⭐ | ⭐ | ⭐ | 8 | Low |
| 3 | Chalhoub (2016) | ⭐ | ⭐ | ⭐ | ⭐ | ⭐ | ⭐ | ⭐ | ⭐ | 8 | Low |
| 4 | Ferreira (2022) | ⭐ |  | ⭐ |  | ⭐ | ⭐ |  | ⭐ | 5 | Moderate |
| 5 | Hughes-Austin (2020) |  | ⭐ | ⭐ | ⭐ | ⭐⭐ | ⭐ | ⭐ | ⭐ | 8 | Low |
| 6 | Longo (2022) |  |  | ⭐ |  |  | ⭐ |  | ⭐ | 3 | High |
| 7 | Luo (2024) | ⭐ | ⭐ | ⭐ | ⭐ | ⭐⭐ | ⭐ | ⭐ | ⭐ | 9 | Low |
| 8 | Ribeiro (2020) | ⭐ | ⭐ | ⭐ | ⭐ | ⭐ | ⭐ | ⭐ | ⭐ | 8 | Low |
| 9 | Semba (2012) | ⭐ | ⭐ | ⭐ | ⭐ | ⭐⭐ | ⭐ | ⭐ |  | 8 | Low |
| 10 | Semba (2016) | ⭐ | ⭐ | ⭐ | ⭐ | ⭐⭐ | ⭐ | ⭐ |  | 8 | Low |
| 11 | Shardell (2015) |  | ⭐ | ⭐ | ⭐ | ⭐⭐ | ⭐ | ⭐ |  | 7 | Low |
| 12 | Shardell (2016) |  | ⭐ | ⭐ | ⭐ | ⭐⭐ | ⭐ | ⭐ |  | 7 | Low |
| 13 | Shardell (2019) | ⭐ | ⭐ | ⭐ | ⭐ | ⭐⭐ | ⭐ | ⭐ |  | 8 | Low |
| 14 | Shardell (2020) |  | ⭐ | ⭐ | ⭐ | ⭐⭐ | ⭐ | ⭐ |  | 7 | Low |
| 15 | Valenzuela (2019) |  | ⭐ | ⭐ | ⭐ |  | ⭐ | ⭐ | ⭐ | 6 | Moderate |
| 16 | Wei (2019) | ⭐ | ⭐ | ⭐ | ⭐ | ⭐⭐ | ⭐ |  | ⭐ | 8 | Low |
| 17 | Yun (2020) | ⭐ | ⭐ | ⭐ | ⭐ | ⭐⭐ | ⭐ | ⭐ | ⭐ | 9 | Low |

**Supplementary Table S4.** Quality assessment of Case Control Studies with Newcastle – Ottawa Quality Assessment Scale

|  |  | **Selection** | | | | **Comparability** | **Exposure** | | |  |  |
| --- | --- | --- | --- | --- | --- | --- | --- | --- | --- | --- | --- |
|  | Study | Case Definition | Representativeness of cases | Selection of Control | Definition of control | Comparability of cases and controls on the basis of the design or analysis | Ascertainment of exposure | Same method of ascertainment for cases and control | Non-Response rate | Total Stars | Overall Bias |
| 1 | Ahmadi (2016) | ⭐ | ⭐ | ⭐ | ⭐ | ⭐ | ⭐ | ⭐ | ⭐ | 8 | Low |
| 2 | Cui (2024) | ⭐ | ⭐ | ⭐ | ⭐ | ⭐ | ⭐ | ⭐ | ⭐ | 8 | Low |
| 3 | Çelebi (2025) | ⭐ |  | ⭐ | ⭐ | ⭐⭐ | ⭐ | ⭐ | ⭐ | 8 | Low |
| 4 | Desbiens (2018) | ⭐ | ⭐ | ⭐ | ⭐ | ⭐⭐ | ⭐ | ⭐ | ⭐ | 9 | Low |
| 5 | Desbiens (2022) | ⭐ | ⭐ | ⭐ | ⭐ | ⭐⭐ | ⭐ | ⭐ | ⭐ | 9 | Low |
| 6 | Ellidağ (2016) | ⭐ | ⭐ |  | ⭐ | ⭐ | ⭐ | ⭐ | ⭐ | 7 | Low |
| 7 | Gözükara (2019) | ⭐ | ⭐ | ⭐ |  | ⭐ | ⭐ | ⭐ | ⭐ | 7 | Low |
| 8 | Jiang (2024) | ⭐ | ⭐ | ⭐ |  | ⭐ | ⭐ | ⭐ | ⭐ | 7 | Low |
| 9 | Kuriyama (2017) | ⭐ |  | ⭐ | ⭐ | ⭐⭐ | ⭐ | ⭐ | ⭐ | 8 | Low |
| 10 | Sartorius (2019) | ⭐ | ⭐ | ⭐ | ⭐ | ⭐⭐ | ⭐ | ⭐ | ⭐ | 9 | Low |
| 11 | Ushigusa (2016) | ⭐ | ⭐ |  | ⭐ | ⭐⭐ | ⭐ | ⭐ | ⭐ | 8 | Low |

**Supplementary Table S5.** Quality assessment of Randomized Controlled Studies with Cochrane Risk-of-Bias 2 Tool

| **No** | **Study Author** | **Risk of Bias** | **Inconsistency** | **Indirectness** | **Imprecision** | **Publication Bias** | **Overall** |
| --- | --- | --- | --- | --- | --- | --- | --- |
| 1 | Amaro-Gahete Exercise (2019) ^[17]^ | Low risk | Low risk | Some concern | Low risk | Low risk | Some concerns |
| 2 | Amaro-Gahete (2024) | Low risk | Low risk | Some concerns | Low risk | Low risk | Some concerns |
| 3 | Collins (2023) | Low risk | Low risk | Some concerns | Low risk | Low risk | Some concerns |
| 4 | Correa (2021) | Low risk | Low risk | Low risk | Low risk | Some concerns | Some concerns |
| 5 | Fakhrpour (2020) | Some concerns | Low risk | Low risk | Low risk | Low risk | Some concerns |
| 6 | Gaitan (2021) | Low risk | Low risk | Low risk | Low risk | Low risk | Low risk |
| 7 | Ghadamyari (2024) | Some concerns | Low risk | Low risk | Low risk | Low risk | Some concerns |
| 8 | Snigdha (2024) | Low risk | Low risk | Low risk | Low risk | Low risk | Low risk |
| 9 | Vazquez-Lorente (2024) | Low risk | Some concerns | Low risk | Low risk | Low risk | Some concerns |
| 10 | Middelbeek (2021) | Low risk | Low risk | Low risk | Low risk | Low risk | Low risk |
| 11 | Neves (2020) | Low risk | Low risk | Low risk | Low risk | Low risk | Low risk |

**Supplementary Table S6.** Quality assessment of Quasi-experimental studies with JBI Critical Appraisal Tool

| **No** | **Study Name** | **Q1** | **Q2** | **Q3** | **Q4** | **Q5** | **Q6** | **Q7** | **Q8** | **Q9** | **Quality** |
| --- | --- | --- | --- | --- | --- | --- | --- | --- | --- | --- | --- |
| 1 | Boeselt (2017) | Yes | Yes | Yes | Yes | Yes | Yes | Yes | Yes | Yes | 9/9 |
| 2 | Ercan (2022) | Yes | Yes | Yes | Yes | Yes | Yes | Yes | Yes | Yes | 9/9 |
| 3 | Iturriga (2021) | Yes | No | Yes | No | Yes | Yes | Yes | Yes | Yes | 7/9 |
| 4 | Matsubara (2014) | Yes | Yes | Yes | Yes | Yes | Yes | Yes | Yes | Yes | 9/9 |
| 5 | Morishima (2021) | Yes | No | Yes | No | Yes | Yes | Yes | Yes | Yes | 7/9 |
| 6 | Rahimi (2018) | Yes | Yes | Yes | No | Yes | Yes | Yes | Yes | Yes | 8/9 |
| 7 | Rangraz (2023) | Yes | Yes | Yes | Yes | Yes | Yes | Yes | Yes | Yes | 9/9 |
| 8 | Saghiv (2017) | Yes | Yes | Yes | Yes | Yes | Yes | Yes | Yes | Yes | 9/9 |
| 9 | Tan (2018) | Yes | No | Yes | No | Yes | Yes | Yes | Yes | Yes | 7/9 |
| 10 | Tetik Dündar (2023) | Yes | No | Yes | No | Yes | Yes | Yes | Yes | Yes | 7/9 |

**Supplementary Table S7**. Baseline Characteristics of Included Studies*

| **Study** | **Study design** | **Country** | **Studied population**  **(n)** | **Age, years (Mean ±SD)/ Female (%)** | **Klotho levels** | **Inclusion criteria** | **Exclusion criteria** |
| --- | --- | --- | --- | --- | --- | --- | --- |
| **Frailty and Multidimensional Geriatric Assessment** | | | | | | | |
| **Branco (2023)[8]** | Single-center, prospective study | Portugal | Incident patients with peritoneal biopsy (58) | Biopsy score:  S0:59(median)/  42%;  S1: 61/33%;  S2:54/18% | Peritoneal dialysis failure: 724 pg/ml  Major cardiovascular event: 698 pg/ml | Patients over 18 years of age and having a stable clinical condition, defined by the absence of serious abdominal infections or active neoplasia, and on peritoneal dialysis with biopsy of the peritoneal membrane | History of previous aggressions to the peritoneal membrane (such as surgeries or peritonitis) |
| **Chuang (2023)[9]** | Population-based cohort study | Taiwan | Adults aged 40-79 years (13583) | 56.1±0.2/52.2% | Group 1:≤575.8  Group 2:575.9-678.8 Group 3:678.9-761.0 Group 4:761.1-849.9 Group 5:850.0-961.3 Group 6: 961.4-1140.0  Group 7:≥1140.1 pg/ml. | Middle-aged and older adults aged 40 to 79 years | Individuals without soluble Klotho testing, whose survival status was not available, who had received dialysis in the past 12 months, who had no serum creatinine data, or who had eGFR < 30 mL/min/1.73 m |
| **Guan (2023)[10]** | Cross-sectional study | China | Middle-Aged and Older Adults  (7107) | ≥45/ 48.1% | 838.0 pg/mL | Adults 45 years or older, with available Klotho levels and frailty assessment data | Aged <45 years or with missing data on Klotho and frailty |
| **Jiang**  **(2023)[4]** | Cross-sectional study | China | People aged 40 to 79 (7052) | 62.76±0.18/ 51.05 | Q1: ≤641.775 pg/ml;  Q2: 641.775–785.7 pg/ml;  Q3: 785.7–973.9 pg/ml;  Q4: >973.9 pg/ml | Participants aged 40 to 79 years | Pregnancy or incomplete data |
| **Koyama (2015)[11]** | Cross-sectional study | Japan | People aged over 60 with CKD (52) | 78.2±8.2/ 61.5% | 787.6 pg/ml (median) | People aged over 60 | Infection, neoplastic disease, and kidney injury |
| **Li (2023)[12]** | Cross-sectional | USA | People aged  40 to 79 years (4388) | 58.1±11.0/51.1% | Q1: 152.5–611.9 mg/ml  Q2: 612.4–733.9 pg/ml  Q3: 734.0–857.1 pg/ml  Q4: 857.2–1048.4 pg/ml  Q5: 1048.8–3829.7 pg/ml | Participants aged 40–79 years with available serum Klotho and phenotypic age data | Participants with missing data on key covariates, including smoking status, sleep, hypertension, alcohol use, BMI, education, marital status, and energy intake |
| **Longo (2022)[13]** | Longitudinal study | Brazil | Older adult women (15) | 66.0±6.2 / 100% | Light physical activity strongly correlated with Klotho | Adults with no cognitive decline, eligible for regular physical activity | Adults with unstable cardiovascular disease, infections, tumors, or knee/hip prosthesis |
| **Luo (2024)[14]** | Population-based longitudinal study | USA | Frail individuals (2608) | 60.78 ±10.48 / 59.89% | 829.20±329.02 pg/  ml. | Frail middle-aged and elderly individuals with a frailty index ≥ 0.25 | Participants under 40 years of age, those assessed as non-frail, those lacking serum Klotho data, and those with missing mortality follow-up information |
| **Polat (2020)[15]** | Cross-sectional study | Turkey | Patients ≥65, classified by frailty status (89) | Non-frail group: 72.70±4.45/61.4%  Frail group:  79.36±6.91/68.9% | Non-frail group: 0.76±1.01 ng/ml;  Frail group:  0.54±0.61 ng/ml | Elderly adults from Ankara geriatric outpatient | Physical disability, active infection, non-remission malignancy, acute disease, advanced dementia, recent hospitalization/surgery (<1 month), or cognitive impairment |
| **Sagi (2020)[16]** | Observational study | Hungary | 96 hemodialysis patients + 20 controls | 65.06 ± 14.09 / 48.9% | Hemodialysis patients:  299.3 ± 124.7 pg/ml  Controls: 602 ± 100 pg/ml | Consecutive chronic hemodialysis patients in stable clinical condition treated in 2 dialysis centers | Individuals with lower extremity amputation, acute infection, malignancy, acute myocardial infarction, pulmonary edema, or hemodynamic instability |
| **Semba (2012)[17]** | Longitudinal study | USA | Community-dwelling adults aged ≥65 years (804) | 75 (median)/ 55.8% | 681 pg/mL, | Participants ≥65 years from the InCHIANTI study | Age <65 years |
| **Shardell (2019)[18]** | Prospective cohort study | USA | Community-dwelling adults aged ≥65 years (774) | Klotho ≤660 group:  74.6±7.1/ 52.7%  Klotho >660 group:  73.0±6.2/ 59.2% | ≤660 pg/ml: n=387  >660 pg/ml: n=387 | Participants ≥65 years from the InCHIANTI study | Age <65 years |
| **Yuan (2024)[19]** | Retrospective study | USA | Middle-aged and older adults aged 40-79 years (9597) | 40–79 / 49.6% | Q1: 534.30 pg/ml;  Q2: 683.20 pg/ml; Q3: 800.00 pg/ml; Q4: 942.95 pg/mL | Participants aged 40-79 years with complete data on klotho, frailty index, relevant covariates | The lack of KL, frailty index, relevant covariates |
| **Zelazniewicz (2022)[20]** | Cross-sectional study | Poland | Healthy men between 30-45 years (189) | 35.33±3.47/ 0% | 1144.38±461.85 pg/ml | No diagnosed chronic disease (DM, cardiovascular disease, etc.), no hormonal treatment, not smoking, no ongoing infections | Age below 30 years, elevated CRP (> 10 μg/ml), indicating inflammation, or missing data |
| **Zhang (2022)[21]** | Cross-sectional | USA | Participants aged 40–79 years (10928) | 56.00(median)/  51.45% | 805.20 (657.58–1001.12) pg/ml | Participants who completed a full 24-hour dietary history and underwent serum Klotho testing | Participants with incomplete dietary recall or serum Klotho data, and those diagnosed with cancer or stroke |
| **Physical Function, Exercise, and Mobility Interventions** | | | | | | | |
| **Aczel (2023)[22]** | Cross-sectional study | USA | Subjects between the ages of 37 and 85  Trained group (131)  Sedentary group (71) | 37–85/ 38.9% (trained), 61.9% (sedentary) | Trained group Woman:  5.39±0.15 pg/ml  Men:  5.92±0.09 pg/ml  Sedentary group  Woman:  5.71±0.15 pg/ml  Men:  6.13±0.23 pg/ml | Subjects between the ages of 37 and 85 | N/A |
| **Amaro-Gahete (2019)[23]** | RCT | Spain | Middle-aged adults (45-65 years old) (74) | 53.4 ± 5.0/52.7% | In all patients:  805.1±358.8 pg/ml;  In PAR group:  714.3±294.5 pg/ml;  In HIIT group:  788.5±276.8 pg/ml;  In HIIT-EMS group:  808.5±499.0 pg/ml | Participants who were sedentary, and had maintained a stable weight for the previous six months. | History of cardiovascular disease, diabetes, pregnancy or lactation, beta-blocker use, or major illness limiting exercise ability |
| **Amaro-Gahete (2019)[2]** | RCT | Spain | Middle-aged sedentary adults (74) | 53.7 ± 5.1/52.7% | **All participants: 775±364 pg/mL Men: 814±452 pg/mL; Women:741±266 pg/mL** | Participants who were sedentary, non-smokers, not taking any medication, had no acute or chronic illnesses, maintained a stable body weight in the last 3 months (<3 kg change), and were not pregnant. | Participants who did not wear the accelerometers for at least 16h/day during 4 days (including 1 weekend day) |
| **Amaro-Gahete (2024)[24]** | RCT | Spain | Young adults  aged between 18 and 25 years (144) (110 completed) | 22.2 ± 2.2/ 68.2% | 789.0±506.4 pg/ml | Aged 18–25, sedentary, stable weight, BMI 18.5–35 | Non-smoker, no exercise contraindication, BMI 18.5–35 kg/m², sedentary, stable body weight during the last 12 weeks, not taking any medication for chronic illness. |
| **Arroyo (2023)[25]** | Cross-sectional study | India | Adults **20 years and older** (80) | ≥20/ 50% | 20-34 years group: 916.1 pg/ml;  35-49 years group: 829.6 pg/ml;  0-64 years group: 722.6 pg/ml;  ≥ 65 years group: 703.0 pg/ml | Participants ≥20 years with serum in Biobank, completed chair stand and grip strength tests, and without current/past systemic or musculoskeletal diseases | Age <20 years |
| **Boeselt (2017)[26]** | Prospective study | Germany | Chronic obstructive pulmonary disease patients:  Exercise group (31)  Controls (18) | 65.73 ± 8.3/29.7% | Exercise (6. months)  633±235 pg/ml; Control (6 months)  602±158 pg/ml | Chronic obstructive pulmonary disease patients without exacerbations for at least 4 weeks | N/A |
| **Chen (2024)[27]** | Cross-sectional study | China | Subjects aged ≥40 years, grouped by weight change patterns (10972) | 54.0 (median)/ 51% | 858.0±308.9 pg/ml | Participants aged 40 years or older from NHANES 2007–2016 cycle | Pregnancy, age <40, self-reported cancer, energy intake <500 or >5000 kcal/day, missing data for baseline BMI, recalled body weight at both age 25 and 10 years before recruitment, or Klotho |
| **Collins (2023)[28]** | RCT | USA | Participants aged 18–55 years in diet and physical activity intervention groups (383) | 45.4 ± 8.0/ 77.6% | Responders:  936.2 pg/ml;  Non-responders: 926.1 pg/ml | Participants aged 18 to 55 years with a body mass index between 25 and <40 kg/m^2^ | Self-reporting ≥60 min/week physical activity, ≥5% weight loss in past 6 months, bariatric surgery, cardiometabolic disease, diabetes, cancer, use of medications affecting heart rate, blood pressure, body weight; psychological treatment, pregnancy; planning relocation, non-compliance, contraindications to cardiac MRI |
| **Correa (2021)[29]** | RCT | Brazil | Patients with stage 2 CKD (105) | Resistance training group:  58±6/ 34.2%  Controls: 58±5/ 31.4% | Increased Klotho levels in the resistance training group | Patients diagnosed with stage 2 CKD based on eGFR | Decompensated patients, CKD ≥ stage 3; neurodegenerative, osteoarticular, autoimmune, or congenital renal diseases, lupus, or comorbidities limiting physical activity |
| **Crasto (2012)[30]** | Cross-sectional study | Italy | Community-dwelling adults aged ≥65 years (802) | ≥ 65/ 55.9 % | With activities of daily living disability: 574 pg/ml;  Without: 676 pg/ml | Participants ≥65 years from the InCHIANTI study | Age <65 years |
| **Ercan**  **(2023)[31]** | Case-control study | Turkey | Rheumatoid arthritis patients (40)  Controls (40) | Patients:  45.30±9.59/72.5%  Controls:  44.13±6.31/67.5% | Postexercise  Patients:  1.84±1.08 ng/ml;  Controls:  1.71±1.16 ng/ml | Diagnosed with Rheumatoid arthritis, on stable medical treatment for at least 3 months, aged 18–65 years, and presenting with low disease activity | Pregnancy, malignancy, neurological involvement, immobility, cooperation problems, autoimmune or inflammatory diseases, regular exercise, use of anti-TNF therapy, presence of cardiac symptoms |
| **Fakhrpour (2020)[32]** | RCT | Iran | Hemodialysis patients (45) | 61 ± 9.02/ 15.5% | Exercise group:  397.25±133.36 pg/ml;  Controls: 362.93±147.05 pg/ml | Adult hemodialysis patients that can cooperate and tolerate the exercise program, on dialysis 3 sessions /week for more than 6 months | Patients with severe peripheral neuropathy, orthopedic limitations, cardiovascular disease, or other medical conditions preventing exercise during dialysis |
| **Ghadamyari (2024)[33]** | RCT | Iran | Overweight/  obese postmenopausal women with T2DM (30) | Endurance exercise 55.12±2.85/ 100%  Resistance exercise  58.12±3.18/ 100%  Control  55.75±3.61/ 100% | Endurance exercise: 1.83±0.18 ng/ml;  Resistance exercise:  1.79±0.14 ng/ml;  Control:  1.42±0.10 ng/ml | Postmenopausal (≥5 years) women aged 50–60, overweight/obese (BMI ≥25), T2DM >1 year, taking metformin, with poor glycemic control and no history of regular exercise | Hormone therapy, smoking ≥6 months, and chronic diseases (cardiovascular, pulmonary, cerebrovascular, fatty liver diseases, bone disorders, diabetic, deformities/ nephropathy) |
| **Girginer (2019)[34]** | Cross-sectional study | Turkey | Healthy male participants  60 healthy males (EMAG: n=22, RG: n=21, Sedentary: n=17) | 45–65/ 0% | Elite master athletes: 820.08±335.65 pg/ml;  Recreational athletes: 796.70±357.13 pg/ml;  Controls: 569.94±229.52 pg/ml | Males aged 45–65, living at sea level for at least 10 years. Elite master athletes: training for more than 10 years, at least 5 days/10 h/week. Recreational athletes: training for at least 10 years, at least 3 days/3h/week. Controls: sedentary, no exercise | Chronic diseases, regular medical treatment, Vit D/CK supplements, a BMI beyond the acceptable range, musculoskeletal disorders, smoking |
| **Iturriaga (2021)[35]** | RCT | Spain | Healthy, physically active men (82) | 29.9±9/ 0% | Cardiorespiratory exercise: 1172.28±238.14 pg/ml;  Strength exercise: 1001.97±231.78 pg/ml | Men aged 18–55 years, non-smokers, training at least three times a week for 6 months or longer, no musculoskeletal injuries | Maximal oxygen consumption below 40 ml/kg/min, incomplete exercise intervention, missing blood samples, and no consent |
| **Matsubara (2014)[36]** | Cross-sectional study | Japan | Healthy and postmenopausal women (aged 50–76 years old) (69) | 60±1/ 100% | Before intervention: 414 ± 28 pg/ml;  After intervention: 453 ± 36 pg/ml | Healthy postmenopausal women aged 50–76 years | N/A |
| **Middelbeek (2021)[37]** | RCT | USA | Healthy middle‐aged sedentary men (22) | 48±5/ 0% | Sprint intensity training: 4.3 ng/ml;  Moderate intensity training: 5.9 ng/ml | Age 40–55 years, BMI 18.5–30 kg/m^2^, VO_2Peak_ up to 40 mL/kg/min. | N/A |
| **Morishima (2021)[38]** | Pre-post study | Japan | Young, untrained, male subjects (12) | 20.4±0.3/ 0% | Klotho significantly increased following exercise | Untrained healthy males **(**BMI: 17.5±2.4 kg/m^2^**),** nonsmokers, no cardiovascular, pulmonary, metabolic or neurological disease | N/A |
| **Mostafidi (2016)[39]** | Case-control study | Iran | Trained healthy football players (males aged  18 – 22 years) (30)  Controls (28) | 18–22/ 0% | Athletes:  3.375±1.48 ng/ml  Controls:  1.39±0.43 ng/ml | Trained healthy male footballers aged 18-22 | N/A |
| **Rahimi (2018)[40]** | Case-control study | Iran | Athlete women (10)  Controls (10) | Athletes  31 ± 9/ 0%  Controls:  32 ± 7/ 0% | 72h after exercise Athletes:  549.5±80.4 pg/ml  Controls:  452.8±83.2 pg/ml | Premenopausal athlete women (30 ± 10 years), exercising regularly (at least 5 times/week for 2 hours), with no medication history, nonsmokers, and free of renal, thyroid, diabetes, liver, and cardiovascular diseases | N/A |
| **Rangraz (2023)[41]** | Semi-experimental study | Iran | Physically inactive young women (20) | 22–30/ 100% | Post-test  Aerobic exercise:  1433.33±314.11  pg/ml;  No exercise:  1000±209.76 pg/ml | Age range of 22-30 years and a BMI of 20-24 kg/m^2^ | Regular sports activity, chronic diseases, movement problems, drug use, and intake of antioxidant and nutritional supplements |
| **Saghiv**  **(2017)[42]** | Case-control study | Israel | CAD patients with aerobic exercise (group A) (41)  CAD patients without active exercise (group B) (17) | Group A:  59.6±2.2/ 26.8% Group B:  61±2.4/ 29.4% | Group A:  863.4±213.66 pg/ml;  Group B:  677.7±167.46 pg/ml | Group A: CAD patients, who had a recent (<45 days) myocardial infarction, **p**ercutaneous intervention, aorto coronary by- pass surgery | Renal/liver dysfunction, uncontrolled hypertension, neurological or orthopedic diseases |
| **Saghiv (2017)[43]** | Comparative Study | USA | National level male powerlifters (50)  Age matched untrained young male adults (50) | Powerlifters:  27.1±1.0/ 0%  Controls:  26.5±1.0/ 0% | Powerlifters:  421.0±76 pg/ml;  Controls:  435.2±89.0 pg/ml | Young adults following chronic powerlifting resistance training at for three or more years and, age matched untrained young adult subjects | N/A |
| **Santos Rosa (2020)[44]** | Cross-sectional study | Brazil | Master sprinters (13), master endurance runners (18), middle-aged untrained controls (12), young untrained controls (17) | Master sprinters: 50 ± 8.9 years, Master endurance: 53 ± 8.2 years, Middle-aged controls: 45.5 ± 9.8 years, young controls: 22.7 ± 3.9 years / 0% | Master sprinters had the highest levels (674 ± 96 pg/mL), followed by young controls (683 ± 101 pg/mL), master endurance runners (522 ± 133 pg/mL), and the lowest in middle-aged controls (489 ± 59 pg/mL) | For master athletes: male individuals aged 40 to 65 years, with at least 20 years of continuous training and current participation in national or international competitions. For control groups: healthy, untrained males within defined age ranges (20–30 years for young controls, 40–65 years for middle-aged controls) | Not clearly defined; individuals not meeting inclusion criteria were excluded |
| **Shardell (2015)[45]** | Prospective study | Italy | Participants **55 years and older** (860) | Klotho >669:  74.3±7.6/59.0%  Klotho ≤669: 76.3±8.2/52.1% | >669 pg/mL: n =432;  ≤669 pg/mL: n= 428 | Participants ≥55 years from the InCHIANTI study | Age <55 years |
| **Shardell (2020)[46]** | Cross-sectional study | USA | Community-dwelling older adults (2751) (687 with CKD) | 74.0 (median)/ 51.1% | CKD group:  589.4 pg/mL  Non-CKD group: 644.5 pg/mL | Older adults enrolled in Health ABC Study who were free of mobility disability, had no life-threatening illness, had no plans to leave the area for 3 years | Adults who had difficulty walking, life-threatening illness, plan to leave area in 3 years. |
| **Snigdha (2024)[47]** | RCT | India | Sedentary, community-  dwelling older adults aged 60–80 years (258= yoga: 132, control: 126) | 66 (median)/ 55.4% | N/A | Able to perform moderate yoga and/or with lifestyle diseases (e.g., diabetes, obesity, hypertension) | Recent engagement in any regular structured yoga, history of chronic or neurological diseases, any recent surgery limiting the practice of yoga |
| **Tan (2018)[48]** | Cohort study | Australia | Healthy adult volunteers  aged >18 years old (10) | 49 (median)/ 50% | Prior to exercise (T_pre_):  483 (423–767) pg/ml  Post-exercise (T_post_):  602 (514–831) pg/ml | Healthy adults aged >18 years, nonsmokers, not on medication affecting exercise capacity | History of cardiovascular/chronic kidney disease, DM, psychological/medical illness precluding informed consent, and physical disability limiting treadmill use |
| **Tetik Dündar (2023)[49]** | Pre–post study | Turkey | Athletes who were engaged in cross-country skiing at elite level (20) | 19.80±2.587/ 45% | Prior to test:  1.25 ± 0.35 ng/ml;  2 hours after the test:  2.61 ± 2.02 ng/ml;  48 hours after the test:  5.41 ± 5.67 ng/ml | Cross-country skiing at elite level, non-smokers, no regular medication use, cardiovascular disorders or hormone treatment history | N/A |
| **Valenzuela (2019)[50]** | Prospective cohort study | Spain | Male hemodialysis patients (30) | 71±9 / 0% | 358±135 pg/mL | Male dialysis patients with end-stage renal disease, and with available outcome data | Patients with recent cardiovascular events, musculoskeletal or respiratory complications, uncontrolled hypertension, peripheral vascular disease, chronic active liver disease, difficult vascular access |
| **Cognitive, Neuropsychiatric, and Psychological Health** | | | | | | | |
| **Ahmadi (2016)[51]** | Retrospective observational case-control study | Iran | New RRMS (15)  Chronic RRMS (15)  Controls (15) | Control: 30.93±9.72  New RRMS: 28.86±8.04 Chronic RRMS:  35.33±6.70/  Control: 93.3% New RRMS: 86.6%  Chronic RRMS:  60% | New RRMS:  585.56±153.99 pg/mL  Chronic RRMS: 696.94±170.52 pg/mL  Controls: 556.81±120.36 pg/mL | New RRMS: Patients with active RRMS who had not previously received any immunosuppressive, immunomodulatory, or vitamin D treatment.  Chronic RRMS: Patients with active RRMS for more than 3 years, undergoing regular treatment with interferon beta-1a. | Not specified |
| **Barbosa (2017)[52]** | Case-control study | Brazil | BD patients (40) Controls (30) | BD Patients: 50.28 ± 12.80;  Controls: 45.87 ± 9.61/  Patients: 60%  Controls: 73.2% | BD Patients:  165.89  (86.3-241.1) pg/mL  Controls: 73.95  (44.8-111.0) pg/mL | Type I BD diagnosed by Mini-International  Neuropsychiatric Interview (MINI-Plus) | Psychiatric disorder, family history of psychiatric disorder, cognitive deficits, renal, autoimmune or infectious diseases; use of anti-inflammatory, corticosteroids, or antibiotics within last 4 weeks |
| **Brombo (2018)[53]** | Cross-sectional study | Italy | Elderly individuals (≥65 years old) (320):  60 patients with normal cognition, 60 patients with vascular dementia, 100 patients with late-onset Alzheimer’s disease | Normal:  72.4 ± 9.6  Vascular demans: 78.4 ± 5.7  Alzheimer’s disease:  74.9 ± 7.1/  Normal: 63.3%  Vascular demans: 78%  Alzheimer’s disease: 59% | 1^st^ tertile:  ≤514.8 pg/mL,  2^nd^ tertile:  514.8–659.1 pg/mL,  3^rd^ tertile:  ≥659.1 pg/mL | Elderly individuals (≥65 years old) admitted from 2008 to 2015 to the Memory Clinic of the Department of Internal Medicine and Geriatrics | Not specified |
| **Cui (2024)[54]** | Randomized stratified study | China | Elderly patients with Mild cognitive impairment aged 60 years (161)  Controls (161) | Cognitive impairment:  68.48±7.15/49.7%  Controls: 67.27±6.87/50.3% | Cognitive impairment:  85.9±13.7 ng/ml;  Controls:  121.6±24.8 ng/ml | Memory loss confirmed by others, MoCA score between 19 and 26, objective evidence of mild cognitive impairment based on the Mini-Mental State Examination, reduced daily and social functioning, HIS score ≤4, disease duration longer than 3 months, does not meet the diagnostic criteria for dementia | Mental illness, congenital mental retardation, and depression, neurological diseases, history of head trauma, drug use, severe infections, toxic encephalopathy, and chronic conditions such as heart, lung, liver, or kidney disease, hypertension, and diabetes |
| **Çelebi (2025)[55]** | Case control study | Turkey | Male, bipolar disorder patients (48)  Controls (48) | Bipolar disorder:  37.31±10.82/ 0% Controls:  36.89±7.22/ 0% | Bipolar disorder:  0,12±0,15 ng/ml;  Controls:  0,17±0,16 ng/ml | Male patients diagnosed with bipolar disorder who attended the outpatient psychiatry clinics of SEAH | Patients with comorbid psychiatric disorders, chronic medical conditions, mental disabilities, alcohol/substance use disorders, or electroconvulsive therapy in the past six months |
| **Ellidag (2016)[56]** | Case-control study | Turkey | 63 (32 RRMS, 31 controls) | RRMS:  38.3±9.9 Controls: 38.5±7.5/  RRMS:  65.6%  Controls: 62.2% | RRMS:  3.31 (3.1–4.8) ng/ml  Controls:  2.25 (1.8–3.6) ng/ml | Patients with a clinically definite diagnosis of multiple sclerosis ccording to McDonald criteria | Patients with probable MS or clinically isolated syndrome or any other acute or chronic inflammatory disease, anaemia, malignancy, cerebrovascular disease, kidney disease, pregnancy, or patients using any antioxidant drugs that could influence the results |
| **Emir (2023)[57]** | Case-control study | Turkey | Patients with BD manic episodes (42)  Healthy controls (41) | 38.47±10.71/  0% | BD patients during manic episode: 1.05±0.75 ng/ml;  Controls: 0.51±0.29 ng/ml | Diagnosed with BD according to DSM-5 criteria, men, aged between 18 and 65 years, who voluntarily agreed to participate in the study, were literate, able to comprehend and complete the assessment scales used, and had no chronic somatic disorders, inflammatory diseases, or immune system disorders. | Mental retardation, alcohol and/or substance abuse, chronic somatic disorders, malignancy, presence of an active infection, use of corticosteroids or any drugs affecting the immune system within the last six months, a diagnosis of rheumatologic disease, illiteracy, and failure to provide written informed consent. |
| **Gaitan (2021)[58]** | RCT | USA | Middle-aged adults, with familial/genetic risk for Alzheimer’s disease (23) | Physical activity  group:  63.92±5.19/ 50%  Enhanced physical activity: 65.88±4.0/ 45.4% | No change after exercise | Asymptomatic, aged 45–80, cognitively healthy, physically inactive, with familial/genetic risk for Alzheimer’s disease | N/A |
| **Gao (2021)[59]** | Cross-sectional study | China | MDD patients (144)  Healthy controls (112) | Group 1: Young (30–40)  Group 2: Elderly (>65)/  Elderly: 54.3%  Young:65.7%  Control: 59.4% | MDD patients: 714.767 ± 313.075  Controls:  808.660 ± 312.629 pg/ml | Diagnosed by two experienced psychiatrists based on Diagnostic and Statistical Manual of Mental Disorders-IV; age 30-40 or >65 | Neurological/psychiatric disorder other than MDD; a history of substance abuse or dependency |
| **Ge (2024)[60]** | Cross-sectional study | USA | Older adults aged 60–79 years (2173) | 67.4/ 53% | Q1:<657.8 pg/mL  pg/ml  Q2:657.8 -797.7 pg/ml  Q3:797.8-958.2 pg/ml  Q4: ≥958.3 pg/ml | Participants who had available information on serum klotho levels and cognitive function performance | Participants who had available information on serum klotho levels and cognitive function performance |
| **Guo (2017)[61]** | Cross-sectional study | China | 180 (53 MSA patients, 65 4 Parkinson’s disease, 62 controls) | MSA patients:  30±56.6  Parkinson: 38±58.5  Controls:  34±54.8/  MSA patients:  43.4%  Parkinson: 41.5%  Controls: 45.2% | MSA patients:  2.41±1.51  Parkinson: 2.23±1.94  Controls:  3.06±1.28 | Patients who meet the consensus criteria for clinical diagnosis of MSA and the United Kingdom Parkinson's Disease Society Brain Bank criteria (UK-PDSBB) for diagnosis of Parkinson’s disease | Medication affecting vitamin D metabolism, Familial Parkinsonism, Lewy body dementia, cerebrovascular disease/sequelae. |
| **Kato**  **(2024)[62]** | Cross-sectional study | Japan | Hemodialysis patients aged≥65 years without diagnosed dementia (390) | 74 (median)/ 32.6% | 381 (300–517) pg/ml | Hemodialysis patients 65 years of age and older, who were not diagnosed with or treated for dementia and did not have medical conditions that could affect cognitive function | Underwent dialysis for less than 3 months and fewer than three times per week, had acute medical conditions, or had an inability to measure serum factors related to CKD-MBD |
| **Kılıcaslan (2022)[63]** | Case-control study | Turkey | 80 (40 schizophrenia, 40 controls) | Schizophrenia: 37.2 ± 11.1  Controls:  37.4 ± 11.9/  0% | Schizophrenia:  0.585 (0.16-2.03) ng/ml  Controls:  0.480 (0.15-1.89) ng/ml | Diagnosed with schizophrenia (DSM-5), male, aged 18–65 years, literate, able to understand study scales, volunteered to participate, without chronic somatic, inflammatory, or immune diseases. | Intellectual disability; alcohol/substance abuse; chronic/malignant disease; antioxidant use; active infection; immunological disease; corticosteroid/ immunosuppressive drug use in the past 6 months; illiteracy; lack of written informed consent. |
| **Kundu (2022)[64]** | Cross-sectional observational study | USA | Aged 39 to 83+ subjects (94) | 39–83+/47.8% | Klotho is associated with higher MMSE scores and lower clinical dementia ratings. | Subjects with a banked cerebrospinal fluid sample, who were non-smokers, in good general health, and without any past or current major psychiatric or neurological disorders (other than Alzheimer's disease). | N/A |
| **Kuriyama (2017)[65]** | Case Control study | Japan | Participants who underwent a medical check-up of the brain (280) | 70.8/ 33.2% | Groups by white matter lesion grade:  G0: 685.1 pg/ml;  G2: 634.1 pg/ml;  G3: 596.0 pg/ml;  G4: 571.6 pg/ml | Participants who underwent a medical check-up of the brain | Individuals with dementia and a past medical history of stroke and symptomatic brain hemorrhage |
| **Linghui (2023)[66]** | Cross-sectional study | USA | American  older adults (2171) | 67.58±5.46/ 51.45% | 851.52±294.07 pg/ml | Participants aged 60–79 years | N/A |
| **Prather (2015)[67]** | Cross-sectional observational study | USA | 178 healthy premenopausal women | 42.0±5.1  / 100% | Women under high chronic stress:  836.0± 327.7 pg/ml  Women under low-stress:  949.0± 311.8 pg/ml | Participants aged 20 to 50, who do not smoke, have at least one child aged 2 to 16, are premenopausal, and are in good general health. | Those who have had cancer or undergone chemotherapy or radiation therapy in the last 10 years, or who have a history of coronary heart disease, endocrine disorders, epilepsy, brain damage, autoimmune diseases, severe asthma, or lung disease. |
| **Sanz (2021)[68]** | RCT | Spain | Older individuals living in nursing homes (103) | 84.7±7.0/ 70.9% | 0.97±0.18 ng/ml | Aged ≥70 years; scored ≥50 on the Barthel Index for Activities of Daily Living; scored ≥20 on the MEC-35 test; and were capable of standing up and walking independently for at least ten meters | N/A |
| **Sartorius (2019)[69]** | Observational clinical study | Germany | Patients with a major depressive episode (53): undergoing electroconvulsive therapy (16) or receiving monotherapy with an antidepressant (37)  Controls (39) | Patients with major depressive episode:  45.2 ± 14.3  Controls: 44.5 ± 14.3/  Patients: 47.2%  Controls: 46.2% | Patients:  563.8 ± 153.1 pg/ml  Controls:  573.5 ± 208.7 pg/ml | Patients with major depression (DSM-IV  criteria) with a severity of at least 18 points on the Hamil  ton Depression Rating Scale (HDRS, 21 items).  No history of substance abuse or dependency and no evidence of neurological or relevant medical disorders. | Renal disorder, hypocalcemia, hypercalcemia |
| **Shardell (2016)[70]** | Prospective cohort study | Italy | Participants aged 55 or older without dementia (833) | ≥55/ 55.6% | 669 pg/mL | Participants aged ≥55 at blood draw for Klotho measurement, without a dementia diagnosis at enrollment | N/A |
| **Ushigusa (2016)[71]** | Comparative case-control study | Japan | NPSLE (34),  Controls:  SLE patients, without neurological manifestations (25),  RRMS (20)  Neuromyelitis optica (20),  Viral meningitis (19) | NPSLE:  37.5 (27-45)  SLE:  35 (28–48)/  NPSLE:  88.2%  SLE:  92% | NPSLE:  256 (127–309) pg/ml  SLE:  234 (160–382) pg/ml | Fulfilling at least four of the 11 revised American College of Rheumatology criteria for the classification of SLE and diagnosed with NPSLE by both rheumatologists and psychiatrists. | Uncertain NPSLE diagnosis, infections, corticosteroid-induced symptoms |
| **Vanquez-Lorente (2024)[72]** | RCT | Spain | Middle-aged sedentary adults  aged 45–65 years old (74) | 53.6±5.1/ 52.7% | Baseline: 775.3±363.7 pg/ml  Post intervention: 1070.4±460.3 pg/ml | Physically inactive, with stable weight in the past 12 weeks, free of disease, not pregnant or breastfeeding, non-smoker, not taking any medication | N/A |
| **Wu (2023)[73]** | Cross-sectional observational study | China | Older adults aged 60–79 years (1875) | 60-79/ 51.7% | Tertile 1: ≤ 704.2 pg/ml;  Tertile 2: 704.2−913.3 pg/ml;  Tertile 3:  > 913.3 pg/ml | Older adults aged 60–79 years | Participants who had not data on klotho, cognitive function, covariates |
| **Wu (2024)[74]** | Cross-sectional  study | China | Patients with non-alcoholic fatty liver disease (356) | 66 (medium)/ 49.4% | Q1: ≤ 656.6) pg/ml;  Q2: 656.7–801.3 pg/ml;  Q3: 801.4–983.7 pg/ml;  Q4: > 983.7 pg/ml | Participants over 60 years of age who met the diagnostic criteria for **non-alcoholic fatty liver disease** | Age <60, missing serum Klotho or cognitive data, heavy alcohol consumption, or positive for hepatitis B antigen, hepatitis C antibody, or HCV RNA |
| **Xiong (2020)[75]** | Cross-sectional study | China | Patients with schizophrenia (40)  Controls (40) | Patients: 30.36±7.97/ 45%  Controls: 33.23±8.57/52.5% | Patients: 1233.25±382.25 pg/ml;  Controls: 844.49±302.94 pg/ml | Patients aged 18–60, with ≥9 years of formal education, diagnosed with schizophrenia via SCID, PANSS score 60–120, no psychiatric or physical disorders, including recent cerebral infarction or pregnancy | Patients with neurological disorders, mental retardation, and drug/alcohol abuse or addiction |
| **Yalcin (2025)[76]** | Cross-sectional case-control study | Turkey | Patients with Parkinson’s Disease (157) Controls (157) | Patients: 72.09±5.9/61.32% | Patients:  0.85 nmol/L  Controls:  1.47 nmol/L | The presence of Parkinson’s Disease and being 60 years old | Comprised non-volunteer status, uncontrolled chronic disease, acute infection, acute decompensation of chronic disease, and active malignancy |
| **Yokoyama (2017)[77]** | Cross-sectional | USA | Healthy older adults aged 55-90 (136) | 74±0.7/49.2 | KL-VS genotype (heterozygotes): 891.7±45.3 pg/ml  Non-carriers: 780.9±19.6 pg/ml  KL-VS genotype (homozygotes):  599.1±75.7 pg/ml | Healthy older adults undergoing multistep screening, including neurologic exam, detailed cognitive assessment, and medical history | Individuals with a family history of autosomal dominant neurodegenerative or neuropsychiatric disease, and those with known disease-related mutations |
| **Zhang**  **(2023)[5]** | Cross-sectional  study | China | CKD patients with albuminuria (368) | 68 (median)/4.2% | 781.8 pg/ml | Patients with **Urine albumin to creatinine ratio** > 30mg/g from the NHANES | **Patients with urine albumin to creatinine ratio <30mg/g, and missing data of cognitive tests and serum Klotho** |
| **Zhang**  **(2023)[6]** | Cross-sectional study | China | Patients with diabetes (292)  Controls (150) | With mild cognitive impairment:  60.54±7.22/ 45% Without mild cognitive impairment:  59.07 ± 6.85/ 46.2% | With mild cognitive impairment:  260.9±33.9 pg/ml;  Without mild cognitive impairment:  296.6±33.5 pg/ml | The age range of 50 to 75, T2DM diagnosis made at least a year prior, and capacity to comprehend and comply with research procedures | Diabetes complications (e.g., hypoglycemia, coma, ketoacidosis), neurological or cerebrovascular disease, major organ diseases, mental illness (e.g., Parkinson’s, epilepsy), and recent use of drugs that may affect cognitive function |
| **Zhu (2018)[78]** | Prospective cohort study | China | CKD patients undergoing  hemodialysis (58)  Controls (20) | With **normal cognitive function:** 43.10±7.80/48.3%  With mild cognitive decline:  48.00±7.20/48.3%  Controls: 45.00±8.90/45% | Klotho levels were lower in the normal **cognitive function** and mild cognitive impairment groups compared to controls. | CKD patients undergoing hemodialysis | History of intracranial surgery, neurological or mental diseases (e.g., dementia, Alzheimer’s, schizophrenia), brain trauma, or use of antiepileptics, alcohol, or illicit drugs. |
| **Musculoskeletal Function, Aerobic Capacity** | | | | | | | |
| **Amaro-Gahete (2019)[79]** | Cross-sectional study | Spain | Middle-aged sedentary adults (74) | 53.7 ± 5.1/52.7% | All: 775.3±363.7 pg/ml  Men: 814.1±452.2 pg/ml;  Women: 741.4± 265.6 | Age 40–65, sedentary lifestyle, non-smoker, stable weight in last 3 months, normal ECG | Age <40 or >65 years |
| **Baldan[80] (2015)** | Case-control study | Italy | β-thalassemia major patients (106)  Healthy controls (95) | β-TM: 38.6 ± 6.5;  Control: 40.9 ± 7.8/  β-TM: 72%; Control: 28% | Normal bone status:  681.2±184.7 pg/ml; Osteopenic: 549.2±156.5 pg/ml;  Fragility fracture: 520.1±160.5 pg/ml | Age 30–49, Caucasian, normal renal function | Fractures due to major trauma |
| **Chalhoub (2016)[81]** | Longitudinal cohort study | USA | Community-dwelling adults (3075) | 78.7/ 52% | Q1: 397.8 pg/ml  Q2: 554.5 pg/ml  Q3: 709.9 pg/ml  Q4: 999.5 pg/ml | Free from life-threatening cancers; planning to remain in the study area for at least ≥3 years | Inability to perform daily physical activities (such as walk, climb stairs) |
| **Desbiens (2018)[82]** | Prospective cohort study | Canada | Hemodialysis patients (130) | 72± 63.77/ 53.1% | Non-fracture group: 314 pg/ml  Fracture group:  292 pg/ml | Age >18, stable hemodialysis for ≥3 months, No history of fractures in the past month | Fractured 1 month before recruitment |
| **Desbiens (2022)[83]** | Case-cohort study | Canada | Eligible population (18913)  Case-cohort population (312=159 non-CKD; 153 CKD) | 53/ 50.7% | Non-CKD: 713 pg/ml;  CKD: 651 pg/ml | Age 40–69, eGFR <60 mL/min/1.73 m^2^ for CKD, >60 mL/min/1.73 m^2^ for non-CKD | No plasma sample, eGFR <30, dialysis, renal replacement therapy, kidney transplant, CKD stage 4–5, teriparatide use, no creatinine measurement at baseline |
| **Ferreira (2021)[84]** | Cross-sectional  study | Portugal | Renal transplanted patients (69) | 53.0 (median)/ 31.3% | 945.2 pg/ml | Age 18–66, renal transplant, Patients who underwent to a bone biopsy | Double transplantation, major cognitive dysfunction, outside age range |
| **Ferreira (2022)[85]** | Prospective observational cohort study | Portugal | Renal transplanted patients (69) | 53.0 (median)/ 31.3% | Baseline:  571.0 pg/ml;  One year after transplantation:  945.2 pg/ml | Age 18–66 | Double transplantation, major cognitive dysfunction, outside age range |
| **Fukasawa (2014)[86]** | Cross-sectional study | Japan | Haemodialysis patients (77) | 67.0 (median)/ 32% | All: 613 pg/ml;  Men: 596 pg/ml;  Women: 614 pg/ml | Aged less than 85 years and undergoing hemodialysis for at least three consecutive months | N/A |
| **Gözükara (2019)[87]** | Case-control study | Turkey | Participants **65 years and older** (89= case: 43 controls: 46) | 75 (median)/ 65% | Case group:  408.9 pg/ml  Controls:  455.9 pg/ml | Patients >65 years diagnosed with sarcopenia according to EWGSOP criteria. | Recent cardiovascular events, chronic obstructive pulmonary disease exacerbation, malignancy, infection, malnutrition, advanced dementia, immobility, or comorbidities affecting muscle mass measurement |
| **Han (2018)[88]** | Community-based cross-sectional study | China | Postmenopausal women (355) | 62.9 ± 8.8/ 100% | LgKlotho:  Normal BMD: 2.34±0.21;  Osteopenia: 2.33±0.21; Osteoporosis: 2.28±0.23 | Postmenopausal women, ≥35 years, no history of disease or drug use, not any health conditions, good cognitive and physical ability, able to take care of themselves, sign the informed consent. | Non-menopausal woman aged under 35 |
| **Huang (2024)[89]** | Cross-sectional study | China | Middle-aged and elderly patients with End-stage renal disease (87) | 58.9 ± 13.8/34.5% | Normal bone mass: 0.54±0.14 ng/ml;  Low bone mass: 0.45±0.10 ng/ml | Age >45, the duration of the dialysis was > 3 months, complete data, no change in their recent weight, no history of fractures within the past 6 months. | Severe disease, history of transplantation, malignancy, mental disorder, use of hormone or immunosuppressive therapy, incomplete data,  pregnancy/lactation |
| **Hughes-Austin (2020)[90]** | Longitudinal  study | USA | CKD patients who underwent bone biopsy (39)  Participants in the Health  ABC cohort with CKD (641) | Biopsy group: 64±13/ 85%  Health ABC cohort: 75±3/49% | Biopsy group: 713±257 pg/ml; Health ABC cohort: 589 pg/ml | Age ≥18, patients who underwent bone biopsy | History of renal transplantation, parathyroidectomy, on dialysis, use of medications known to affect bone metabolism, malignancy, systemic illnesses |
| **Jiang**  **(2023)[3]** | Retrospective study | China | Postmenopausal women (871) | Non-osteoporosis group:  61.9±7.5/ 100%  Osteoporosis group:  65.7±7.5/ 100% | LgKlotho:  Non-osteoporosis group: 9.70 pg/ml;  Osteoporosis group: 9.57 pg/ml | Age>50, Postmenopausal women | Age <50, regular menstruation periods in past 12 months, missing data |
| **Jiang (2024)[91]** | Case-control study | China | Elderly patients with osteoporosis (62)  Controls (62) | Study group: 79.32±7.11/51.6%  Controls:  76.34±6.24/46.7% | Study group: 883.7±40.24 pg/ml; Control: 673.5±41.4 pg/ml | Age ≥60, confirmed osteoporosis (via BMD) and single vertebral osteoporotic fracture, signed informed consent | Secondary osteoporosis, serious disease, recent bone-affecting drugs, infection, mental illness, malignancy, poorly controlled diabetes mellitus, hyperthyroidism |
| **Katsaounis**  **(2023)[92]** | Case-control study | Greece | Patients with liver cirrhosis (74)  Controls (25) | Patients: 59.2 (median)/ 33%  Controls:57/ 32% | Patients:  1.051 pg/ml;  Controls:  1.842 pg/ml | Cirrhosis confirmed by biopsy, clinical evaluation, or imaging | Long-term use of glucocorticoids or osteoporosis medications |
| **Kureya (2016)[93]** | Cross-sectional | Japan | Non-smokers (16), smokers (13) without COPD, and  smokers with COPD (24) | 62-78/ N/A | Smokers without COPD:  400 pg/mL;  Non-smokers without COPD: 354 pg/mL;  Smokers with COPD: 250 pg/ml | Non-smokers, smokers without COPD, and smokers with COPD | Subjects with main comorbidities such as endocrine  disorders, hypertension, cardiovascular disorders, and renal  impairment |
| **Kuzmova (2021)[94]** | Cross-sectional study | Slovakia | CKD outpatients (74)  (According to eGFR levels: G1–3, G4–5) | G1–3: 70.5 ± 11.4  G4–5: 68.5±11.1/  43.2% | G1–3:  0.176±0.15 ng/ml;  G4–5:  0.195±0.1 ng/ml | Diagnosed with CKD (according to KDIGO guidelines); no specific osteoporosis treatment other than vitamin D or calcium | Acute or chronic illness, hospitalization, immobility, secondary osteoporosis, specific osteoporosis treatment |
| **Lima (2019)[95]** | Cross-sectional  study | USA | Patients with CKD stages 2 – 5D underwent  bone biopsies (104)  Controls (19) | 59 ± 15/ 72.1%, | Controls:720 pg/ml;  CKD 2: 837 pg/ml;  CKD 3: 669 pg/ml;  CKD 4/5: 691 pg/ml;  CKD 5D: 530 pg/ml | Age ≥18, diagnosed with CKD stages 2-5D | History of renal transplantation, parathyroidectomy, use of medications affecting bone metabolism, malignancy, hepatic disease, active infection |
| **Marchelek-Mysliwiec (2018)[96]** | Case-control study | Poland | Patients with CKD in stage 2–4 (52)  Controls (23) | Patients: 60.07±9.8  Controls: 53.7±13.2 (p=0.059) | Patients: 168.9±166.3 ng/ml  Controls:  280.45±376.01ng/ml | CKD patients in stages 2–4 with eGFR of 15–60 ml/min/1.73 m² | Osteoporosis, inflammation, malignancy, thyroid disease, use of glucocorticosteroids, anticoagulants, erythropoietin, bisphosphonates |
| **Martin-Gonzalez (2023)[97]** | Cross-sectional study | Spain | Participants aged **18 years and older** diagnosed with SLE (195)  Controls (169) | SLE patients:  51 ± 17/ 95%  Controls:  50±11 / 96% | No differences between SLE patients and controls | Participants ≥18 years with clinical SLE diagnosis and ≥4 American College of Rheumatology criteria met | Cancer, inflammatory rheumatic disease, chronic disease, active infection |
| **Matei (2022)[98]** | Cross-sectional study | Romania | Kidney transplant recipients (59)  Volunteers (59) | Recipients:  44 ± 1.5/ 49.1%  Volunteers:  43.7 ± 1.9/ 49.1% | Recipients:  1.37 ± 0.13 ng/ml | Kidney transplant recipients aged 20–60 with a successful kidney transplant | Pregnancy, dialysis, graft rejection, urinary tract infections, bone-active therapy (except vitamin D), diabetes, menopause,malignancy, secondary osteoporosis other than CKD-MBD |
| **Neves (2021)[99]** | RCT | Brazil | Maintenance hemodialysis patients (193) (Control (60), DRT (66), IRT (67)) | Control:  55±12/ 42.3%)  DRT:  58±15/ 43.1%  IRT:  56±19 / 39.2% | Control:108±63 pg/ml  DRT: 279±51 pg/ml  IRT: 156 ± 65 pg/ml | Age ≥18, GFR <15 mL/min/1.73 m², hemodialysis duration > 3 months, clinically stable (no major metabolic, cardiovascular, or orthopedic complications), blood pressure <180/100mmHg | Severe metabolic decompensation, regular exercise, recent stroke (<6 months), dialysis adequacy < 1.2, recent arteriovenous fistula access (<3 months), autoimmune diseases |
| **Pedersen (2020)[100]** | Cross-sectional | Denmark | Healthy adults aged 19–66 years (120) | 39.5 (27–54)/51.7% | 472±137 pg/ml | Healthy blood donors aged 19-66 years | N/A |
| **Ribeiro (2020)[101]** | Prospective study | Portugal | T2DM patients with CKD stage 2–3 (126)  (26 with bone fractures and 100 without)  Controls (27) | 58.10 ± 6,66/ 41.8% | With fractures: 113.31±13.89 pg/ml;  Without fractures: 379.08±15.07 pg/ml;  Controls: 490.67±8.76 pg/ml | T2DM with CKD stage 2–3, stable clinical condition | Bone disease, age <18 or >75, uncontrolled hypertension, albumin/  creatinine ratio >500, eGFR ≤29 or >90, T1DM, neoplastic/  infectious diseases, non-diabetic renal diseases, parathyroid hormone ≥350, phosphorus >5.5, use of vitamin D, phosphate binders, anticoagulants |
| **Salamanna (2024)[102]** | Cohort study | Italy | Participants ≥40 years with or without osteopenia, sarcopenia, or osteosarcopenia (18) | ≥40/ 67% | Lower in osteosarcopenic patients compared to those with sarcopenic and osteopenic patients | Patients ≥40 with degenerative lumbosacral spine conditions undergoing posterior vertebral surgery | Patients with tumor- or trauma-related spine conditions, a history of musculoskeletal infections, or prior spinal surgery |
| **Sawada (2024)[103]** | Cross-sectional study | Brazil | Elderly participants (55) | 85.63±4.02/ 78% | 733.43±360.83 pg/ml | Elderly participants aged ≥80 years living independently in the community | Dementia, severe acute/ chronic illness, dialysis, chemo/radiotherapy, neoplasm, hospitalization within 3 months, history of cerebrovascular accident or heart attack, sensory deficits impairing questionnaire response |
| **Semba (2016)[104]** | Prospective cohort study | USA | Older adults (1983) | 74.5±2.9/ 49.6% | Q1: :<536 pg/ml  Q2: 536–747 pg/ml  Q3: >747 pg/ml | Participants from the Health ABC Study (community-based cohort); functionally independent, no life-threatening illness, no plans to leave the area for 3 years | Use of steroids, systolic ≥200 or diastolic blood pressure ≥100 mmHg, history of cerebral aneurysm/bleeding, knee replacement, severe knee/hand pain, or recent surgery |
| **Teng-Lee (2023)[105]** | Cross-sectional study | Taiwan | Chronic hemodialysis patients from outpatient clinic (43) | 59.4±12.3/ 34.9% | 102±75,161 pg/ml | Aged 20–84, receiving hemodialysis ≥3 months | Myocardial infarction or stroke within180 days, infection-related hospitalization (<90 days), Bone mineral density <17 kg/m², malignancy history, or missing BMD data |
| **Wang (2024)[106]** | Cross-sectional study | China | NHANES participants (3635) (Q1: without alveolar bone loss, Q2–4: with bone loss)  Living kidney recipients (71) | 56±10.29/ 58.8% | Q1: 0.8955±0.3177 ng/mL  Q2: 0.8558±0.2904 ng/mL  Q3: 0.8521±0.3375 ng/mL  Q4: 0.8410±0.2905 ng/mL | Aged >30, free from trauma or severe periodontitis  Age >30, no trauma, without severe periodontal disease; living kidney transplant recipients | Incomplete records of periodontal attachment loss examination, attachment loss <5 mm, and missing data on α-Klotho level |
| **Wei (2019)[107]** | Cross-sectional study | China | Hemodialysis patients (88) | 57±14/ 36.4% | All patients:  119.10±47.29 pg/ml; With cerebrovascular disease:  91.65±28.19 pg/ml;  Without cerebrovascular disease:  131.90±49.09 pg/ml | Hemodialysis patients | Patients with a history of stroke or without head CTor MRI in the past year, with an active psychiatric disorder, Alzheimer's disease, or significant visual or hearing impairment |
| **Xie (2024)[108]** | Cross-sectional study | USA | Middle-aged adults: Aged 40–59 (3803) | 49.68±0.14/ 50.93% | Normal muscle mass 0.81±0.00 pg/ml;  Low muscle mass 0.51±0.01 pg/ml | Aged 40 to 59 years | Missing data on appendicular lean mass, BMI, serum Klotho, or use of estrogen-based drugs |
| **Yun (2020)[109]** | Prospective study | South Korea | Hemodialysis patients (57) (with and without a past cardiovascular events) | 56.8±15.9/ 50.9% | With cardiovascular events:  474.6±370.0 ng/ml  Without:  582.8±740.1 ng/ml | Hemodialysis patients aged over 19 years | Cirrhosis, pacemaker, chemotherapy, pregnancy, psychiatric disorder |
| **Zhang (2023)[7]** | Cross-sectional  study | China | Individuals aged 40–79 years from the NHANES database (3120) | 49.27±5.62/ 50.57 | Q1: <678.6 pg/ml  Q2: 678.6–821.7 pg/ml  Q3: 821.8–1018.7 pg/ml  Q4: >1018.7 pg/ml | Adult subjects aged 40–79 years | Missing bone mineral density or covariate data, and presence of cancer or malignancy |
| **Zheng (2018)[110]** | Cross-sectional study | China | Maintenance hemodialysis patients with normal bone mass, osteopenia, or osteoporosis (125) | 63.35±14.69/ 41.6% | Normal bone mass: 429.88±41.77 ng/ml;  Osteopenia: 410.59±61.05 ng/ml;  Osteoporosis: 387.17±54.13 ng/ml | Maintenance hemodialysis patients | Autoimmune disease, malignancy, hepatitis, cirrhosis, thyroid disease, infection, malnutrition, mental disorder |

*: Although we categorized the studies into four subgroups, certain studies could reasonably fit into more than one category; in such instances, we assigned each to the most appropriate subgroup and selected the title accordingly.

**Abbreviations: BD:** Bipolar disorder, **BMD**: Bone mineral density, **BMI:** Body mass index, **CAD:** Coronary artery disease, **CKD**: Chronic kidney disease, **CKD-MBD:** Chronic kidney disease-mineral and bone disorder, **COPD:** Chronic obstructive pulmonary disease, **DRT:** Dynamic resistance training, **EMAG**: Elite master athletes performing high-intensity training, **HIIT**: High-intensity interval training group, **HIIT-EMS**: High-intensity interval training with whole-body electromyostimulation, **IRT:** Isometric resistance training, **PAR**: Physical activity recommendation from the WHO**, MDD**: Major depressive disorder, **MSA:** Multiple system atrophy, **NPSLE:** Neuropsychiatric systemic lupus erythematosus, **RCT:** Randomized controlled trial, **RG:** Recreational athletes performing moderate-intensity training, **RRMS:** Relapsing-remitting multiple sclerosis**,**  **SLE:** Systemic lupus erythematosus, **T2DM:** Type 2 diabetes mellitus.

**Supplementary Table S8**. **Full** **citations of included studies**

| **No** | **Study** | **Full Text Citation** |
| --- | --- | --- |
| 1 | Aczel 2023 | Aczel D, Torma F, Jokai M, McGreevy K, Boros A, Seki Y, et al. The circulating level of klotho is not dependent upon physical fitness and age-associated methylation increases at the promoter region of the klotho gene. Genes. 2023;14(2):525. https://doi.org/10.3390/genes14020525 |
| 2 | Ahmadi 2016 | Ahmadi M, Emami Aleagha MS, Harirchian MH, Yarani R, Tavakoli F, Siroos B. Multiple sclerosis influences on the augmentation of serum Klotho concentration. J Neurol Sci. 2016;doi:10.1016/j.jns.2016.01.012. |
| 3 | Amaro-Gahete 2019 | Amaro-Gahete FJ, de-la-O A, Jurado-Fasoli L, Gutiérrez Á, Ruiz JR, Castillo MJ. Association of physical activity and fitness with S-Klotho plasma levels in middle-aged sedentary adults: The FIT-AGEING study. Maturitas. 2019;123:25–31. doi:10.1016/j.maturitas.2019.02.001. |
| 4 | Amaro-Gahete 2019 | Amaro-Gahete FJ, De-la-O A, Jurado-Fasoli L, Espuch-Oliver A, de Haro T, Gutierrez A, et al. Exercise training increases the S-Klotho plasma levels in sedentary middle-aged adults: A randomised controlled trial. The FIT-AGEING study. J Sports Sci. 2019;37(19):2175–83. doi:10.1080/02640414.2019.1626048. |
| 5 | Amaro-Gahete 2019 | Amaro-Gahete FJ, De-la-O A, Jurado-Fasoli L, Espuch-Oliver A, de Haro T, Gutiérrez Á, et al. Body composition and S-Klotho plasma levels in middle-aged adults: A cross-sectional study. Rejuvenation Res. 2019;22(5):380–7. doi:10.1089/rej.2018.2092. |
| 6 | Amaro-Gahete 2024 | Amaro-Gahete FJ, Espuch-Oliver A, Cano-Nieto A, Alcantara JMA, García-Lario JV, De Haro T, et al. Impact of 24-week supervised concurrent exercise on S-Klotho and vitamin D levels: A randomized controlled trial. J Sports Sci. 2025 Jan 20. https://doi.org/10.1080/02640414.2025.2453328 |
| 7 | Arroyo 2023 | Arroyo E, Leber CA, Burney HN, Narayanan G, Moorthi R, Avin KG, et al. Relationship between klotho and physical function in healthy aging. Sci Rep. 2023;13:21158. doi:10.1038/s41598-023-47791-5. |
| 8 | Baldan 2015 | Baldan A, Giusti A, Bosi C, Malaventura C, Musso M, Forni GL, et al. Klotho, a new marker for osteoporosis and muscle strength in β-thalassemia major. Blood Cells Mol Dis. 2015;55(4):396–401. |
| 9 | Barbosa 2017 | Barbosa IG, Rocha NP, Alpak G, et al. Klotho dysfunction: A pathway linking the aging process to bipolar disorder? J Psychiatr Res. 2017;95:80–83. doi:10.1016/j.jpsychires.2017.08.007. |
| 10 | Boeselt 2017 | Boeselt T, Nell C, Lütteken L, Kehr K, Koepke J, Apelt S, et al. Benefits of high-intensity exercise training to patients with chronic obstructive pulmonary disease: a controlled study. Respiration. 2017;93(5):365–74. doi:10.1159/000464139. |
| 11 | Branco 2023 | Branco P, Calça R, Martins AR, Mateus C, Jervis MJ, Gomes DP, et al. Fibrosis of peritoneal membrane, molecular indicators of aging and frailty unveil vulnerable patients in long-term peritoneal dialysis. Int J Mol Sci. 2023;24(5):5020. doi:10.3390/ijms24055020. |
| 12 | Brombo 2018 | Brombo G, Bonetti F, Ortolani B, Morieri ML, Bosi C, Passaro A, et al. Lower plasma Klotho concentrations are associated with vascular dementia but not late-onset Alzheimer’s disease. Gerontology. 2018;64(2):141–7. doi:10.1159/000488318. |
| 13 | Celebi 2025 | Çelebi Z, Yazıcı E, Güzel Erdoğan D, Davutoglu O, Yazıcı AB. Relationship between klotho, neurotrophic factors (BDNF, NGF, GDNF) and cognitive functions in patients with bipolar disorder. BMC Psychiatry. 2025;25:53. doi:10.1186/s12888-025-06469-0 |
| 14 | Chalhoub 2016 | Chalhoub D, Marques E, Meirelles O, Semba RD, Ferrucci L, Satterfield S, et al. Association of serum klotho with loss of bone mineral density and fracture risk in older adults. J Am Geriatr Soc. 2016;64(12):e304–8. |
| 15 | Chen 2024 | Chen S, Kong Y, Wang N, Kang N, Chen H, Zhang Z, et al. Association between weight change and serum anti-aging protein α-Klotho: a cross-sectional study in middle-aged and older adults. Sci Rep. 2024;14:18624. doi:10.1038/s41598-024-69556-4. |
| 16 | Chuang 2023 | Chuang M-H, Wang H-W, Huang Y-T, Jiang M-Y. Association between soluble α-klotho and mortality risk in middle-aged and older adults. Front Endocrinol (Lausanne). 2023;14:1246590. |
| 17 | Collins 2023 | Collins KA, Ambrosio F, Rogers RJ, Lang W, Schelbert EB, Davis KK, et al. Change in circulating klotho in response to weight loss, with and without exercise, in adults with overweight or obesity. Front Aging. 2023;4:1213228. doi:10.3389/fragi.2023.1213228. |
| 18 | Corrêa 2021 | Corrêa HL, Neves RVP, Deus LA, Souza MK, Haro AS, Costa F, et al. Blood flow restriction training blunts chronic kidney disease progression in humans. Med Sci Sports Exerc. 2021;53(2):249–57. doi:10.1249/MSS.0000000000002465. |
| 19 | Crasto 2012 | Crasto CL, Semba RD, Sun K, Cappola AR, Bandinelli S, Ferrucci L. Relationship of low-circulating “anti-aging” Klotho hormone with disability in activities of daily living among older community-dwelling adults. Rejuvenation Res. 2012;15(3):295–301. doi:10.1089/rej.2011.1268. |
| 20 | Cui 2024 | Cui L, Gao L, Geng H, Zhang H, Wei H. Analysis of the relationship between mild cognitive impairment and serum klotho protein and insulin-like growth factor-1 in the elderly. Technol Health Care. 2024;32(5):1455-1462. doi:10.3233/THC-230462 |
| 21 | Desbiens 2018 | Desbiens LC, Sidibé A, Ung RV, et al. FGF23-klotho axis, bone fractures, and arterial stiffness in dialysis: a case-control study. Osteoporos Int. 2018;29(10):2345–2353. doi:10.1007/s00198-018-4598-2 |
| 22 | Desbiens 2022 | Desbiens LC, Sidibé A, Ung RV, Mac-Way F. FGF23-klotho axis and fractures in patients without and with early CKD: a case-cohort analysis of CARTaGENE. J Clin Endocrinol Metab. 2022;107(6):e2502–e2512. doi:10.1210/clinem/dgac071 |
| 23 | Ellidag 2016 | Ellidag HY, Yilmaz N, Kurtulus F, et al. The three sisters of fate in multiple sclerosis: Klotho, fibroblast growth factor-23, and vitamin D. Ann Neurosci. 2016;23(3):155–161. doi:10.1159/000449181. |
| 24 | Emir 2023 | Sırlıer Emir B, Yıldız S, Kazğan Kılıçaslan A, et al. The roles of Klotho and FGF-23 in bipolar manic episode. Eur Rev Med Pharmacol Sci. 2023;27(5 Suppl):101–108. |
| 25 | Ercan 2023 | Ercan Z, Deniz G, Yentur SB, Arikan FB, Karatas A, Alkan G, et al. Effects of acute aerobic exercise on cytokines, klotho, irisin, and vascular endothelial growth factor responses in rheumatoid arthritis patients. Ir J Med Sci. 2023;192(2):491–7. doi:10.1007/s11845-022-02970-7. |
| 26 | Fakhrpour 2020 | Fakhrpour R, Tayebi Khosroshahi H, Ahmadizad S, Mesgari Abbasi M, et al. Effect of sixteen weeks combined training on FGF-23, klotho, and fetuin-A levels in patients on maintenance hemodialysis. Iran J Kidney Dis. 2020 May;14(3):205–12. |
| 27 | Ferreira 2021 | Ferreira AC, Mendes M, Silva C, et al. Bone densitometry versus bone histomorphometry in renal transplanted patients: a cross-sectional study. Transpl Int. 2021;34(6):1065–1073. doi:10.1111/tri.13888 |
| 28 | Ferreira 2022 | Ferreira AC, Mendes M, Silva C, et al. Improvement of mineral and bone disorders after renal transplantation. Transplantation. 2022;106(5):e251–e261. doi:10.1097/TP.0000000000004099 |
| 29 | Fukasawa 2014 | Fukasawa H, Ishigaki S, Kinoshita-Katahashi N, Niwa H, Yasuda H, Kumagai H, et al. Plasma levels of fibroblast growth factor-23 are associated with muscle mass in haemodialysis patients. Nephrology (Carlton). 2014;19(12):784–90. doi:10.1111/nep.12333. |
| 30 | Gaitán 2021 | Gaitán JM, Moon HY, Stremlau M, Dubal DB, Cook DB, Okonkwo OC, et al. Effects of aerobic exercise training on systemic biomarkers and cognition in late middle-aged adults at risk for Alzheimer’s disease. Front Endocrinol. 2021;12:660181. doi:10.3389/fendo.2021.660181. |
| 31 | Gao 2021 | Gao X, Sun Z, Ma G, et al. Reduced plasma levels of α-Klotho and their correlation with Klotho polymorphisms in elderly patients with major depressive disorders. Front Psychiatry. 2021;12:682691. doi:10.3389/fpsyt.2021.682691. |
| 32 | Ge 2024 | Ge S, Dong F, Tian C, Yang CH, Liu M, Wei J. Serum soluble alpha-klotho and cognitive functioning in older adults aged 60–79: an analysis of cross-sectional data of the NHANES 2011–2014. BMC Geriatr. 2024;24:245. doi:10.1186/s12877-024-04661-7 |
| 33 | Ghadamyari 2024 | Ghadamyari N, Zolfaghari MR, Tolouei Azar J, Fattahi A. The effect of 8 weeks of endurance and resistance exercises on the serum levels of FGF23 and s-Klotho in type 2 diabetic women. Int J Diabetes Dev Ctries. 2024. doi:10.1007/s13410-024-01343-3. |
| 34 | Girginer 2019 | Girginer F, Büyükyazı G, Ulman C, Doğru Y, Taneli F, Yıldız R, et al. Comparison of some plasma inflammation markers in elite master athletes, recreational athletes and sedentary males. Turkiye Klinikleri J Med Sci. 2019;39(2):202-11. doi:10.5336/medsci.2018-63235 |
| 35 | Gözükara 2019 | Gözükara I, Yılmaz N, Onaran I, Güneş FE, Alkan A, Aydın S, et al. Investigation of the relation between serum Klotho levels and osteoporosis in women. Turk J Med Sci. 2019;49(1):117–23. |
| 36 | Guan 2023 | Guan Z, Ma L, Wu C. Association between serum klotho and physical frailty in middle-aged and older adults: finding from the National Health and Nutrition Examination Survey. J Am Med Dir Assoc. 2023;24(6):1173–1178. doi:10.1016/j.jamda.2023.02.103. |
| 37 | Guo 2017 | Guo Y, Zhuang XD, Xian WB, et al. Serum Klotho, vitamin D, and homocysteine in combination predict the outcomes of Chinese patients with multiple system atrophy. CNS Neurosci Ther. 2017;23(6):512–520. doi:10.1111/cns.12711. |
| 38 | Han 2018 | Han W, Bai XJ, Han LL, Sun XF, Chen XM. The relationship between serum fibroblast growth factor 23, Klotho, and lumbar spine bone mineral density in northern Chinese postmenopausal women. Menopause. 2019;26(5):000–000. doi:10.1097/GME.0000000000001276 |
| 39 | Huang 2023 | Huang T, He Y, Li Y, et al. The relationship between serum fibroblast growth factor 23 and Klotho protein and low bone mineral density in middle-aged and elderly patients with end-stage renal disease. Horm Metab Res. 2024;56(2):142–149. doi:10.1055/a-2168-5089 |
| 40 | Hughes-Austin 2020 | Hughes-Austin JM, Katz R, Semba RD, et al. Biomarkers of bone turnover identify subsets of chronic kidney disease patients at higher risk for fracture. J Clin Endocrinol Metab. 2020;105(8):e2903–e2911. doi:10.1210/clinem/dgaa317 |
| 41 | Iturriaga 2021 | Iturriaga T, Yvert T, Sanchez-Lorente IM, Diez-Vega I, Fernandez-Elias VE, Sanchez-Barroso L, et al. Acute impacts of different types of exercise on circulating α-klotho protein levels. Front Physiol. 2021;12:716473. https://doi.org/10.3389/fphys.2021.716473 |
| 42 | Jiang 2023 | Jiang J, Liu Q, Mao Y, Wang N, Lin W, Li L, et al. Klotho reduces the risk of osteoporosis in postmenopausal women: a cross-sectional study of the National Health and Nutrition Examination Survey (NHANES). BMC Endocr Disord. 2023;23:151. https://doi.org/10.1186/s12902-023-01380-9 |
| 43 | Jiang 2023 | Jiang Z, Wang J, Cai X, Wang P, Liu S. L-shaped association of serum α-Klotho and frailty among the middle-aged and older adults: results from NHANES 2007–2016. BMC Geriatr. 2023;23:716. doi:10.1186/s12877-023-04324-z. |
| 44 | Jiang 2024 | Jiang K, Zhang Z, Hao C, et al. The analysis of serum Klotho protein level and related gene polymorphism in osteoporotic fracture of elderly patients with osteoporosis. Altern Ther Health Med. [Epub ahead of print]. |
| 45 | Kato 2024 | Kato K, Nakashima A, Shinagawa S, Kobayashi A, Ohkido I, Urashima M, et al. Association between serum magnesium levels and cognitive function in patients undergoing hemodialysis. Clin Exp Nephrol. 2024;28:1187-1196. doi:10.1007/s10157-024-02528-0 |
| 46 | Katsaounis 2022 | Katsaounis PV, Hadjiyannis ES, Skaltsi T, Anargyrou VA, Alexopoulou AA, Dourakis SP, et al. Bone disease in patients with cirrhosis of different etiology and severity; are Klotho protein and osteoprotegerin potential biomarkers? Scand J Gastroenterol. 2022;58(2):185–92. https://doi.org/10.1080/00365521.2022.2114813 |
| 47 | Kılıçaslan 2022 | Kazgan Kılıçaslan A, Yıldız S, Sırlıer Emir B, et al. Serum klotho and FGF23 levels in patients with schizophrenia. Psychiatry Clin Psychopharmacol. 2022;32(3):229–236. doi:10.5152/pcp.2022.22406. |
| 48 | Koyama 2015 | Koyama D, Sato Y, Aizawa M, Maki T, Kurosawa M, Kuro-o M, et al. Soluble α-Klotho as a candidate for the biomarker of aging. Biochem Biophys Res Commun. 2015;467(4):1019–1025. doi:10.1016/j.bbrc.2015.10.018. |
| 49 | Kundu 2022 | Kundu P, Zimmerman B, Quinn JF, Kaye J, Mattek N, Westaway SK, et al. Serum Levels of α-Klotho Are Correlated with Cerebrospinal Fluid Levels and Predict Measures of Cognitive Function. J Alzheimers Dis. 2022;86(4):1471–1481. doi:10.3233/JAD-215719. |
| 50 | Kuriyama 2017 | Kuriyama N, Ozaki E, Mizuno T, et al. Association between α-Klotho and deep white matter lesions in the brain: a pilot case control study using brain MRI. J Alzheimers Dis. 2018;61(1):145–155. doi:10.3233/JAD-170466. |
| 51 | Kureya 2016 | Kureya Y, Kanazawa H, Ijiri N, Tochino Y, Watanabe T, Asai K, Hirata K. Down-regulation of soluble α-Klotho is associated with reduction in serum irisin levels in chronic obstructive pulmonary disease. Lung. 2016;194(3):345–51. |
| 52 | Kuzmova 2021 | Kužmová Z, Kužma M, Gažová A, Kovářová M, Jackuliak P, Killinger Z, et al. Fibroblast growth factor 23 and Klotho are associated with trabecular bone score but not bone mineral density in the early stages of chronic kidney disease: results of the cross-sectional study. Physiol Res. 2021;70(Suppl 1):S43–51. https://doi.org/10.33549/physiolres.934773 |
| 53 | Lee 2023 | Lee WT, Fang YW, Chen M, Liou HH, Lee CJ, Tsai MH. Serum Intact Fibroblast Growth Factor 23 Levels Are Negatively Associated with Bone Mineral Density in Chronic Hemodialysis Patients. J Clin Med. 2023;12(4):1550. doi:10.3390/jcm12041550. |
| 54 | Li 2023 | Li H, Miao S, Zhang M, Zhang P, Li YB, Duan RS. U-shaped association between serum klotho and accelerated aging among the middle-aged and elderly US population: a cross-sectional study. BMC Geriatr. 2023;23:780. doi:10.1186/s12877-023-04479-9. |
| 55 | Lima 2019 | Lima F, Mawad H, El-Husseini AA, Davenport DL, Malluche HH. Serum bone markers in ROD patients across the spectrum of decreases in GFR: Activin A increases before all other markers. Clin Nephrol. 2019;91(4):222–30. <https://doi.org/10.5414/CN109650> |
| 56 | Linghui 2023 | Linghui D, Simin Y, Zilong Z, Yuxiao L, Shi Q, Birong D. The relationship between serum klotho and cognitive performance in a nationally representative sample of US adults. Front Aging Neurosci. 2023;15:1053390. doi:10.3389/fnagi.2023.1053390. |
| 57 | Longo 2022 | Longo PL, de Aquino RdC, Ortiz SRM, de Oliveira RS, Gavioli A, do Amaral JB, et al. Effects of physical distancing by COVID-19 pandemic on diet quality, neurological and immunological markers, and fecal microbiota of Brazilian older women. Front Nutr. 2022;9:972100. https://doi.org/10.3389/fnut.2022.972100 |
| 58 | Luo 2024 | Luo H, Zheng Z, Hu H, Sun C. Serum klotho levels and mortality patterns in frail individuals: unraveling the u-shaped association. Aging Clin Exp Res. 2024;36:92. doi:10.1007/s40520-024-02730-w. |
| 59 | Marchelek-Mysliwiec 2018 | Marchelek-Mysliwiec M, Wisniewska M, Nowosiad-Magda M, et al. Association between plasma concentration of Klotho protein, osteocalcin, leptin, adiponectin, and bone mineral density in patients with chronic kidney disease. Horm Metab Res. 2018;50(11):816–821. doi:10.1055/a-0752-4615 |
| 60 | Martin-Gonzalez 2023 | Martin-Gonzalez C, Gomez-Bernal F, Quevedo-Abeledo JC, Ferrer-Moure C, Espelosin-Ortega E, Gonzalez-Gay MA, et al. Alpha-Klotho protein in systemic lupus erythematosus. Clin Exp Rheumatol. 2023;41(1):41–7. |
| 61 | Matei 2022 | Matei A, Bilha SC, Constantinescu D, Pavel-Tanasa M, Cianga P, Covic A, et al. Body composition, adipokines, FGF23-Klotho and bone in kidney transplantation: is there a link? J Nephrol. 2022;35:293–304. <https://doi.org/10.1007/s40620-021-00972-9> |
| 62 | Matsubara 2014 | Matsubara T, Miyaki A, Akazawa N, Choi Y, Ra SG, Tanahashi K, et al. Aerobic exercise training increases plasma Klotho levels and reduces arterial stiffness in postmenopausal women. Am J Physiol Heart Circ Physiol. 2014;306(3):H348–H355. https://doi.org/10.1152/ajpheart.00429.2013 |
| 63 | Middelbeek 2021 | Middelbeek RJW, Motiani P, Brandt N, Nigro P, Zheng J, Virtanen KA, et al. Exercise intensity regulates cytokine and klotho responses in men. Nutr Diabetes. 2021;11(1):5. doi:10.1038/s41387-020-00144-x. |
| 64 | Morishima 2021 | Morishima T, Ochi E. Impact of a single bout of resistance exercise on serum Klotho in healthy young men. Physiol Rep. 2021;9(21):e15087. doi:10.14814/phy2.15087. |
| 65 | Mostafidi 2016 | Mostafidi E, Moeen A, Nasri H, Hagjo AG, Ardalan M. Serum Klotho Levels in Trained Athletes. Nephro-Urol Mon. 2016;8(1):e30245. doi:10.5812/numonthly.30245 |
| 66 | Neves 2021 | Neves RVP, Corrêa HL, Deus LA, Reis AL, Souza MK, Simões HG, et al. Dynamic not isometric training blunts osteo-renal disease and improves the sclerostin/FGF23/Klotho axis in maintenance hemodialysis patients: a randomized clinical trial. J Appl Physiol (1985). 2021;130(2):508–16. |
| 67 | Pedersen 2013 | Pedersen L, Pedersen SM, Brasen CL, Rasmussen LM. Soluble serum Klotho levels in healthy subjects. Comparison of two different immunoassays. Clin Biochem. 2013;46(12):1079–1083. doi:10.1016/j.clinbiochem.2013.05.046. |
| 68 | Polat 2020 | Polat Y, Yalcin A, Yazihan N, et al. The relationship between frailty and serum alpha klotho levels in geriatric patients. Arch Gerontol Geriatr. 2020;91:104225. doi:10.1016/j.archger.2020.104225 |
| 69 | Prather 2015 | Prather AA, Epel ES, Arenander J, et al. Longevity factor klotho and chronic psychological stress. Transl Psychiatry. 2015;5:e585. doi:10.1038/tp.2015.81. |
| 70 | Rahimi 2018 | Rahimi S, Khademvatani K, Zolfaghari MR. Association of circular Klotho and insulin-like growth factor 1 with cardiac hypertrophy indexes in athlete and non-athlete women following acute and chronic exercise. Biochem Biophys Res Commun. 2018;505(2):448–452. doi:10.1016/j.bbrc.2018.09.138. |
| 71 | Rangraz 2023 | Haji Abedin Rangraz M, Hojjat S. The Effects of a Six-Week Aerobic Exercise on Serum Levels of Klotho, IL-10, IL-1, and Oxidative Stress in Sedentary Young Women. Crescent J Med Biol Sci. 2023;10(2):67–72. doi:10.34172/cjmb.2023.11. |
| 72 | Ribeiro 2020 | Ribeiro AL, Mendes F, Carias E, Rato F, Santos N, Neves PL, et al. FGF23-Klotho axis as predictive factors of fractures in type 2 diabetics with early chronic kidney disease. J Diabetes Complications. 2020;34(1):107476. https://doi.org/10.1016/j.jdiacomp.2019.107476 |
| 73 | Saghiv 2017 | Saghiv M, Goldhammer E, Radzishevski E, Sherve C, Maor I, Rosenschein U. The impact of 12 weeks exercise training on circulating soluble-Klotho and Pro-BNP in coronary artery disease patients. Cardiol Vasc Res. 2017;1(1):1-4. |
| 74 | Saghiv 2017 | Saghiv M, Sherve C, Goldhammer E, Ben-Sira D, Sagiv M. Long lasting chronic resistive training effects on circulating S-Klotho and IGF-1. Arch Clin Biomed Res. 2017;1(2):69-75. |
| 75 | Sagi 2020 | Sagi B, Peti A, Lakatos O, et al. Pro- and anti-inflammatory factors, vascular stiffness and outcomes in chronic hemodialysis patients. Physiol Int. 2020;107(2):256–266. doi:10.1556/2060.2020.00026. |
| 76 | Salamanna 2024 | Salamanna F, Faldini C, Veronesi F, Borsari V, Ruffilli A, Manzetti M, et al. A Pilot Study on Circulating, Cellular, and Tissue Biomarkers in Osteosarcopenic Patients. Int J Mol Sci. 2024;25(11):5879. |
| 77 | Sanz 2021 | Sanz B, Arrieta H, Rezola-Pardo C, et al. Low serum klotho concentration is associated with worse cognition, psychological components of frailty, dependence, and falls in nursing home residents. Sci Rep. 2021;11:9098. doi:10.1038/s41598-021-88455-6 |
| 78 | Santos Rosa 2020 | Santos Rosa T, Neves RVP, Deus LA, Sousa CV, Aguiar SS, de Souza MK, Moraes MR, Rosa ECCC, Andrade RV, Korhonen MT, Simões HG. Sprint and endurance training in relation to redox balance, inflammatory status and biomarkers of aging in master athletes. *Nitric Oxide*. 2020 Sep 1;102:42-51. doi:10.1016/j.niox.2020.05.004. Epub 2020 Jun 18. |
| 79 | Sartorius 2019 | Sartorius A, Gilles M, Pfeifer A-M, Deuschle M, Hoyer C, Haffner D, et al. Peripheral levels of the anti-aging hormone Klotho in patients with depression. J Neural Transm (Vienna). 2019;126(6):771–6. doi:10.1007/s00702-019-02008-w. |
| 80 | Sawada 2024 | Sawada KM, Moraes NS, Araújo LM, Gazoni FM, Lazaretti-Castro M, Cendoroglo MS, et al. Blood concentrations of α-Klotho and FGF-23 exhibit no correlation with bone mineral density in elderly individuals. einstein (São Paulo). 2024;22:eAO0412. doi:10.31744/einstein_journal/2024AO0412. |
| 81 | Semba 2012 | Semba RD, Cappola AR, Sun K, Bandinelli S, Dalal M, Crasto C, et al. Relationship of low plasma klotho with poor grip strength in older community-dwelling adults: the InCHIANTI study. Eur J Appl Physiol. 2012;112(4):1215–20. |
| 82 | Semba 2016 | Semba RD, Moghekar AR, Hu J, Sun K, Turner R, Ferrucci L, O'Brien R. Klotho in the cerebrospinal fluid of adults with and without Alzheimer's disease. Neurosci Lett. 2014 Jan 13;558:37-40. doi: 10.1016/j.neulet.2013.10.058. |
| 83 | Shardell 2015 | Shardell M, Semba RD, Kalyani RR, et al. Serum 25-Hydroxyvitamin D, Plasma Klotho, and Lower-Extremity Physical Performance Among Older Adults: Findings From the InCHIANTI Study. J Gerontol A Biol Sci Med Sci. 2015;70(9):1156-1162. doi:10.1093/gerona/glv017 |
| 84 | Shardell 2016 | Shardell M, Semba RD, Rosano C, Kalyani RR, Bandinelli S, Chia CW, Ferrucci L. Plasma klotho and cognitive decline in older adults: findings from the InCHIANTI study. J Gerontol A Biol Sci Med Sci. 2015;70(12):1585–1591. doi:10.1093/gerona/glv140. |
| 85 | Shardell 2019 | Shardell M, Semba RD, Kalyani RR, et al. Plasma Klotho and Frailty in Older Adults: Findings From the InCHIANTI Study. J Gerontol A Biol Sci Med Sci. 2019;74(7):1052-1058. doi:10.1093/gerona/glx202 |
| 86 | Shardell 2020 | Shardell M, Drew DA, Semba RD, Harris TB, Cawthon PM, Simonsick EM, Kalyani RR, Schwartz AV, Kritchevsky SB, Newman AB. Plasma Soluble αKlotho, Serum Fibroblast Growth Factor 23, and Mobility Disability in Community-Dwelling Older Adults. J Endocr Soc. 2020 May;4(5):bvz032. doi:10.1210/jendso/bvz032. |
| 87 | Snigdha 2024 | Snigdha A, Majumdar V, Manjunath NK, Jose A. Yoga-based lifestyle intervention for healthy ageing in older adults: a two-armed, waitlist randomized controlled trial with multiple primary outcomes. GeroScience. 2024;46:6039–6054. doi:10.1007/s11357-024-01149-5. |
| 88 | Tan 2018 | Tan SJ, Chu MM, Toussaint ND, Cai MMX, Hewitson TD, Holt SG. High-intensity physical exercise increases serum α-klotho levels in healthy volunteers. J Circ Biomark. 2018;7:1849454418794582. |
| 89 | Tetik Dündar 2023 | Tetik Dündar S, Kuzucu M, Varol SR. Effect of the aerobic power test performed at low-medium altitude on the myostatin, pgc-1 alpha and klotho levels. Gazz Med Ital Arch Sci Med. 2023;182(6):342. https://doi.org/10.23736/S0393-3660.22.04972-5 |
| 90 | Ushigusa 2016 | Ushigusa T, Ichinose K, Sato S, Michitsuji T, Shimizu T, Umeda M, et al. Soluble α-klotho is a potential biomarker associated with neuropsychiatric systemic lupus erythematosus. Clin Immunol. 2016;165:29–34. doi:10.1016/j.clim.2016.03.001. |
| 91 | Valenzuela 2019 | Valenzuela PL, Cobo F, Diez-Vega I, Sánchez-Hernández R, Pedrero-Chamizo R, Verde-Rello Z, et al. Physical performance, plasma S-klotho, and all-cause mortality in elderly dialysis patients: A prospective cohort study. Exp Gerontol. 2019;122:123–8. doi:10.1016/j.exger.2019.05.003. |
| 92 | Vázquez-Lorente 2024 | Vázquez-Lorente H, De-la-O A, Carneiro-Barrera A, Molina-Hidalgo C, Castillo MJ, Amaro-Gahete FJ. Physical exercise improves memory in sedentary middle-aged adults: Are these exercise-induced benefits associated with S-Klotho and 1,25-dihydroxivitamin D? The FIT-AGEING randomized controlled trial. Scand J Med Sci Sports. 2024;34(3):e14519. doi:10.1111/sms.14519. |
| 93 | Wang 2024 | Wang Z, Xue H, Sun Y, Wang Q, Sun W, Zhang H. Deciphering the Biological Aging Impact on Alveolar Bone Loss: Insights From α-Klotho and Renal Function Dynamics. J Gerontol A Biol Sci Med Sci. 2024;79(9):glae172. doi:10.1093/gerona/glae172. |
| 94 | Wei 2019 | Wei H, Li H, Song X, Du X, Cai Y, Li C, et al. Serum klotho: a potential predictor of cerebrovascular disease in hemodialysis patients. BMC Nephrol. 2019;20:63. doi:10.1186/s12882-019-1232-2. |
| 95 | Wu 2023 | Wu Y, Lei S, Li D, Li Z, Zhang Y, Guo Y. Relationship of klotho with cognition and dementia: Results from the NHANES 2011–2014 and Mendelian randomization study. Transl Psychiatry. 2023;13:337. https://doi.org/10.1038/s41398-023-02632-x |
| 96 | Wu 2024 | Wu F, Pan J, Chen M, Lai X, Gu Y, Pei L, Yang L. Association of serum klotho with cognitive function among individuals with nonalcoholic fatty liver disease. Front Aging Neurosci. 2024;16:1487182. doi:10.3389/fnagi.2024.1487182. |
| 97 | Xie 2024 | Xie Y, Huang K, Li H, Kong W, Ye J. High serum klotho levels are inversely associated with the risk of low muscle mass in middle-aged adults: results from a cross-sectional study. Front Nutr*.* 2024;11:1390517. doi:10.3389/fnut.2024.1390517. |
| 98 | Xiong 2020 | Xiong J, Zhan J, Luo T, Chen H, Wan Q, Wang Y, et al. Increased Plasma Level of Longevity Protein Klotho as a Potential Indicator of Cognitive Function Preservation in Patients With Schizophrenia. Front Neurosci. 2020;14:610. doi:10.3389/fnins.2020.00610. |
| 99 | Yalcin 2025 | Yalcin A, Gemci E, Yurumez B, Yilmaz R, Varli M, Atmis V, et al. Serum alpha klotho levels in Parkinson’s Disease. Neurol Sci. 2025;46:743–749. doi:10.1007/s10072-024-07809-w. |
| 100 | Yokoyama 2017 | Yokoyama JS, Marx G, Brown JA, Bonham LW, Wang D, Coppola G, et al. Systemic klotho is associated with KLOTHO variation and predicts intrinsic cortical connectivity in healthy human aging. Brain Imaging Behav. 2017;11(2):391–400. doi:10.1007/s11682-016-9598-2. |
| 101 | Yuan 2024 | Yuan M, Zhang Y, Hu R, Yuan L, Ma J, Xu Y. Linear-inverse associations of serum Klotho protein with prevalence of frailty among adults in the United States. Am J Transl Res. 2024;16(7):3090–8. |
| 102 | Yun 2020 | Yun HJ, Ryoo SR, Kim JE, Choi YJ, Park I, Shin GT, et al. Trabecular bone score may indicate chronic kidney disease-mineral and bone disorder (CKD-MBD) phenotypes in hemodialysis patients: a prospective observational study. BMC Nephrol. 2020;21:299. doi:10.1186/s12882-020-01944-0. |
| 103 | Zelazniewic 2022 | Zelazniewicz A, Nowak-Kornicka J, Pawłowski B. S-Klotho level and physiological markers of cardiometabolic risk in healthy adult men. Aging (Albany NY). 2022;14(2):708–23. |
| 104 | Zhang 2022 | Zhang C, Zhang Z, Li J, Deng L, Geng J, Jin K, Zheng X, Qiu S, Dong B. Association between Dietary Inflammatory Index and serum Klotho concentration among adults in the United States. BMC Geriatr. 2022;22:528. |
| 105 | Zhang 2023 | Zhang J, Zhang A. Relationships between serum Klotho concentrations and cognitive performance among older chronic kidney disease patients with albuminuria in NHANES 2011–2014. Front Endocrinol. 2023;14:1215977. doi:10.3389/fendo.2023.1215977. |
| 106 | Zhang 2023 | Zhang H, Yu L, Yun G. Reduced Serum Levels of Klotho are Associated with Mild Cognitive Impairment in Patients with Type 2 Diabetes Mellitus. Diabetes Metab Syndr Obes. 2023;16:129–137. doi:10.2147/DMSO.S394099. |
| 107 | Zhang 2023 | Zhang Y, Zhao C, Zhang H, Chen M, Meng Y, Pan Y, et al. Association between serum soluble α-Klotho and bone mineral density (BMD) in middle-aged and older adults in the United States: a population-based cross-sectional study. Aging Clin Exp Res. 2023;35:2039–2049. doi:10.1007/s40520-023-02483-y. |
| 108 | Zheng 2018 | Zheng S, Chen Y, Zheng Y, Zhou Z, Li Z. Correlation of serum levels of fibroblast growth factor 23 and Klotho protein levels with bone mineral density in maintenance hemodialysis patients. Eur J Med Res. 2018;23:18. doi:10.1186/s40001-018-0315-z. |
| 109 | Zhu 2018 | Zhu B, Jin LN, Shen JQ, Liu JF, Jiang RY, Yang L, et al. Differential expression of serum biomarkers in hemodialysis patients with mild cognitive decline: A prospective single-center cohort study. Sci Rep. 2018;8:12250. doi:10.1038/s41598-018-29760-5. |

**Supplementary Table S9**. Summary of the outcome measures and methods of measurement employed in the included studies.

| **No** | **Study** | **Method of α-klotho measurement** | **Exposure or outcomes** | **Method of exposure and outcome measurement** | **Follow-up duration** |  |
| --- | --- | --- | --- | --- | --- | --- |
|  | **Frailty and Multidimensional Geriatric Assessment** | | | | |  |
| 1 | Branco (2023)[8] | ELISA  (Bionava assay) | Peritoneal dialysis-related outcomes | Major cardiovascular event was monitored after 3 months on PD and defined according to validated clinical criteria. It included coronary heart disease, congestive heart failure, acute myocardial infarction, acute cerebral infarction, and cardiac death due to AMI, arrhythmias, or HF | 60 months |  |
|  |  |  | Cardiovascular outcomes  All-cause mortality | Peritoneal dialysis technique failure was defined as ultrafiltration failure, peritonitis, or dialysis inefficacy. Patients who completed 60 months of follow-up were considered to have no technical failure |  |  |
| 2 | Chuang (2023)[9] | ELISA  (IBL Co., Ltd., Japan) | Mortality | Mortality status was determined by linking NHANES data with death records from the National Death Index | Median follow-up: 99 months (interquartile range: 68–129 months) |  |
| 3 | Guan (2023)[10] | ELISA  (IBL Co., Ltd., Japan) | Frailty Assessment | Physical Frailty Phenotype: five criteria: weakness (difficulty lifting 10 lbs), slowness (difficulty walking between rooms), exhaustion (tiredness/low energy), inactivity (highest quintile of sedentary time), and shrinking (BMI <18.5 kg)  Robust: 0 criteria  Prefrail: 1-2 criteria  Frail:3-5 criteria  FI: 34-variable index assessing deficits in health, vital signs, and labs. FI = number of deficits/total deficits.  Robust: FI ≤ 0.10  Prefrail: FI > 0.10 to <0.25  Frail: FI ≥ 0.25 | No follow-up period  Data from NHANES cycles (2007–2016) |  |
| 4 | Jiang (2023)[4] | ELISA  (IBL International Co., Ltd., Japan) | Frailty Assessment | FI:7 categories: cognition (1 item), dependence (16 items), depressive conditions (7 items), comorbidities (13 items), hospital and care (5 items), physical anthropometry (1 item), and laboratory results (6 items) | No follow-up period  Data from NHANES cycles (2007–2016) |  |
| 5 | Koyama  (2015)[11] | ELISA  (IBL Co., Ltd., Japan) | Osteoporosis | Fragility fracture of the vertebral body or proximal femur, or other fractures with BMD <80% of young adult mean, or BMD <70% without fractures | No follow-up; data collected at a single time point (Dec 2014 – Feb 2015) |  |
|  |  |  | Bone Metabolism Factors | Fibroblast Growth Factor 23, phosphorus |  |  |
| 6 | Li (2023)[12] | ELISA  (ImmunoBiological Laboratories,  Gunma, Japan) | Phenotypic age acceleration | Calculated using chronological age and 9 aging-related biomarkers, including albumin, creatinine, glucose, CRP, lymphocyte percent, mean cell volume, red blood cell distribution width, alkaline phosphatase, and white blood cell count | No follow-up period Data from NHANES cycles (2007–2010) |  |
|  |  |  | Physical activity | Calculated according to the metabolic equivalent |  |  |
| 7 | Longo (2022)[13] | ELISA  (R&D Systems, Minneapolis, MN, USA) | BMI | Weight and height measurement | 6 months |  |
|  |  |  | Physical Activity | IPAQ |  |  |
|  |  |  | Diet Quality | Dietary Screening Tool |  |  |
| 8 | Luo (2024)[14] | ELISA (IBL International, Japan) | **Mortality** | Determined via linkage to the National Death Index (NDI), classified according to ICD-10 codes (all-cause, cancer-specific, CVD-specific). | 6.95 years |  |
|  |  |  | **Frailty** Assessment | Assessed using a **Frailty Index based on 49 health deficits**, including domains such as cognition, functional dependence, depression, comorbidities, hospital utilization, general health, physical performance, anthropometry, and laboratory markers (Score ≥ 0.25 = frail) |  |  |
| 9 | Polat (2020)[15] | ELISA  (IBL Co., Ltd., Japan) | Frailty Assessment | Daily Living Activities:   - Basic (Katz ADL, 0–6): Higher = more independent. - Instrumental (Lawton IADL, 0–17): Lower = more dependent   Cognitive Function: MMSE (0–30, lower = impairment)  Depression: Geriatric Depression Scale-15 (≥5 = depression)  Nutritional Status: Mini-Nutritional Assessment Test (≤17 = malnutrition).  Frailty Screening: Turkish FRAIL Scale (0 = robust, 1–2 = pre-frail, 3–5 = frail)  Physical Performance:  Walking Speed: 4-m test (≤0.8 m/s = slow)  Muscle Strength: Handgrip dynamometer | At the 3rd year and 6th year |  |
| 10 | Sagi (2020)[16] | ELISA  (IBL International GmbH, Germany) | Vascular stiffness | Carotid-femoral PWV and augmentation index | 3-year |  |
|  |  |  | Clinical outcomes such as cardiovascular events and mortality | 3-year follow-up |  |  |
| 11 | Semba (2012)[17] | ELISA  (IBL Co., Ltd., Japan) | Hand Grip Strength | JAMAR hand-held isometric dynamometer | Every 3 years for a total of 3 visits:  3-year follow-up (2001–2003, n = 926)  6-year follow-up (2004–2006, n = 844)  9-year follow-up (2007–2009, n = 768) |  |
| 12 | Shardell (2019)[18] | ELISA  (IBL Co., Ltd., Japan) | Frailty Assessment | Frailty: Presence of ≥3 /5 criteria  Prefrailty: 1–2 criteria  Robustness: 0 criteria  Unintentional Weight Loss: >4.5 kg (10 lbs) in the past year (excluding dieting)  Exhaustion: Feeling that “everything was an effort” (Center for Epidemiologic Studies Depression Scale: occasionally/often)  Sedentariness: Self-reported inactivity or <1 hour/week of low-intensity activity  Slow Walking Speed: Lowest quintile for sex and height (measured over 4m)  Muscle Weakness: Grip strength in the lowest quintile for sex and BMI (dynamometer) | 6 years  Assessments: At 3 and 6 years  **α-**Klotho measurement: 3-year visit |  |
| 13 | Yuan (2024)[19] | ELISA  (IBL International Co., Ltd., Japan) | Frailty Assessment | FI:53 Deficits Categories:  Vital signs (Blood pressure, heart rate variability)  Lab values (glucose, kidney function, cholesterol)  Chronic conditions (diabetes, cardiovascular disease, arthritis)  Physical/cognitive (walking speed, grip strength, memory)  Psychosocial (depression, isolation) | No follow-up Data from five NHANES cycles (2007–2016)​ |  |
| 14 | Żelaźniewicz (2022)[20] | ELISA  (IBL Co., Ltd., Japan) | Physical activity | Self-reported through a questionnaire (number and length (in minutes) of training sessions per week and the type of sport practiced) | No follow-up period |  |
|  |  |  | BMI | Weight and height measurement |  |  |
| 15 | Zhang (2022)[21] | ELISA kit  (IBL International, Japan) | Dietary inflammatory index | Calculated based on data from **24-hour dietary recall interviews** conducted as part of the NHANES survey | No follow-up Data from five NHANES cycles (2007–2016)​ |  |
|  | **Physical Function, Exercise, and Mobility Interventions** | | | | |  |
| 16 | Aczel (2023)[22] | ELISA  (R&D Systems, Minneapolis, MN, USA) | Hand Grip Strength | Maximum hand-grip force measured using the CAMRY EH101 dynamometer, three times per hand for ~2 s | No follow-up duration |  |
|  |  |  | Physical Fitness | based on participation in sports activities (trained vs. sedentary |  |  |
|  |  |  | Physical Performance:  VO2 max (the maximum oxygen uptake during intense exercise, indicating cardiorespiratory fitness) | Chester Step Test |  |  |
| 17 | Amaro- Gahete  (2019)[111] | ELISA  (Demeditec, Kiel, Germany) | Physical Activity | Wrist-worn accelerometer measured physical activity and sedentary time | Duration of intervention:12 weeks no long-term follow-up |  |
|  |  |  | Cardiorespiratory fitness | VO₂max, the peak oxygen uptake during intense exercise, assessed via a maximal treadmill test using the modified Balke protocol |  |  |
|  |  |  | Muscular strength:  Lower body strength | isokinetic strength test with a Gymnex Iso-2 dynamometer, focusing on knee flexor and extensor muscles |  |  |
|  |  |  | Muscular strength:  Upper body strength | hand grip strength using a T.K.K. 5401 digital hand dynamometer |  |  |
|  |  |  | Body composition | Measure weight and height →BMI  Assess fat and lean mass via DXA |  |  |
| 18 | Amaro-Gahete (2019)[2] | ELISA  (Demeditec, Kiel, Germany) | Physical activity and sedentary time | Using a wrist-worn accelerometer | No follow-up duration |  |
|  |  |  | Cardiorespiratory fitness | Determined via a maximal treadmill test with indirect calorimetry to estimate VO₂max |  |  |
|  |  |  | Lower-body muscular | Using an isokinetic strength test with a calibrated Gymnex Iso-2 dynamometer |  |  |
|  |  |  | Hand grip strength | Using a digital handgrip dynamometer |  |  |
| 19 | Amaro-Gahete (2024)[24] | ELISA  (Demeditec, Kiel, German) | Anthropometry and body composition | - Body weight   and height were measured using a SECA 799 electronic scale   - BMI (kg/m2)   Fat mass, visceral adipose tissue and lean mass, FMI, LMI measured with DXA | 24 weeks |  |
| 20 | Arroyo (2023)[25] | ELISA  (IBL Co., Ltd., Japan) | Physical Function | Grip Strength: Jamar Plus+ dynamometer  Chair Stand Test:   - 30s Chair Stand - 5-Stand Test   Gait Speed  Static Balance Test  SPPB Score  6MWT  Patient-Reported Outcomes Measurement Information System Physical Function Computerized Adaptive Test  Short Form-36 Physical Function Domain  7-day IPAQ  Total Physical Activity (MET·min/week)  Total Sedentary Time | No follow up duration |  |
| 21 | Boeselt (2017)[26] | ELISA  (IBL Co., Phoenix, AZ, USA) | High-intensity exercise training:  Exercise group: Strength training 2×/week for 3 months  Subgroup: Continued training for additional 3 months | 6-MWT, muscle mass (rectus femoris cross-sectional area) assessed with ultrasonography, and health-related quality of life (St. George’s Respiratory Questionnaire score) | 3 months for all participants (except 6 months for a subgroup of 14 patients) |  |
| 22 | Chen (2024)[27] | ELISA  (IBL Co., Ltd., Japan) | Weight Change | BMI  BMI25: Weight at 25 years / height² (m²)  BMI10prior: Weight 10 years before baseline / height² (m²)  BMIbaseline: Baseline weight / height² (m²) | No follow-up duration |  |
| 23 | Collins (2023)[28] | ELISA  (IBL Co., Ltd., Japan) | Weight loss intervention (diet alone or with exercise) | Body composition: DXA  Cardiorespiratory fitness: treadmill-based submaximal cardiopulmonary exercise test | 12 months |  |
| 24 | Correa (2021)[29] | ELISA  (IBL Co., Ltd., Japan) | Exercise Intervention: Resistance Training with and without Blood Flow Restriction | Renal function,  Inflammation markers,  α-Klotho– Fibroblast  Growth Factor 23 axis | 6 months |  |
| 25 | Crasto (2012)[30] | ELISA  (IBL Co., Ltd., Japan) | ADL disability | Need for help with at least one ADL (bed/chair transfer, bathing, or dressing) | Every 3 years  3-year follow-up:  2001–2003  (n = 926)  6-year follow-up:  2004–2006  (n = 844)  9-year follow-up:  2007–2009  (n = 768) |  |
|  |  |  | Cognitive Impairment | MMSE score < 24 |  |  |
|  |  |  | Physical Activity | Scored 0–7 (inactive to intense); categorized as inactive, low, or moderate-high |  |  |
|  |  |  | Physical Function | Assessed by physiotherapists (muscle strength, gait, balance) |  |  |
|  |  |  | Lower Extremity Function | SPPB (0–12) based on walking speed (4m), five chair rises, and balance. Categorized as <6 (low), 6–10 (moderate), ≥10 (high) |  |  |
| 26 | Ercan (2023)[31] | ELISA  (Elabscience Biotechnology Inc., Houston, Texas) | Exercise Intervention:  Resistance Training + Aerobic Exercise + Blood Flow Restriction for  6 months, 3 sessions/week | Inflammatory markers, **α-**Klotho, Irisin, and VEGF levels | No follow-up duration |  |
| 27 | Fakhrpour (2020)[32] | ELISA  (IBL Co., Ltd., Japan) | Combined aerobic and resistance exercise program, including stationary cycling and lower extremity resistance training | Dialysis-specific questionnaire (quality of life), 1RM test (physical function), **α-**Klotho, Fibroblast Growth Factor 23, Fetuin-A, P, Parathyroid Hormone, ALP | 16 weeks (4 months) |  |
| 28 | Ghadamyari (2024)[112] | ELISA  (Bioassay Technology-Klotho, E4142Hu, China) | Exercise Intervention:   - Endurance exercise | Endurance exercise: Walking and jogging at 70–75% Maximal Heart Rate Reserve for 3 days/week | 8 weeks |  |
|  |  |  | Resistance Exercise | Resistance exercise: 60-minute weight training sessions at 70–75% 1-RM, 3 days/week |  |  |
| 29 | Girginer (2019)[113] | ELISA  (IBL Co., Ltd., Japan) | Long term exercise habit | - Elite Master Athletes (EMAG): High-intensity endurance training for over 10 years - Recreational Athletes (RG): Moderate-intensity training for over 10 years - Sedentary Controls (CG): No regular exercise | No follow-up duration |  |
| 30 | Iturriaga (2021)[35] | ELISA  (IBL Co., Ltd., Japan) | Acute exercise: cardiorespiratory (CR) exercise and strength (ST) exercise | Treadmill running for CR and plyometric exercises for ST | CR Group: No follow-up  ST Group: 72 hours |  |
| 31 | Matsubara (2014)[36] | ELISA  (IBL Co., Ltd., Japan) | Aerobic training (3 days/week, 12 weeks): Supervised cycling/walking (2–3 sessions) + home-based training | Device: Lifecorder pedometer (14 days, except bathing)  Metrics: Daily step count & physical activity level  Estimation: Non-measured activities (e.g., cycling) calculated using 5 MET × time (h) × body mass (kg) × 1.05 | No follow-up (assessed pre- and post-12-week intervention) |  |
| 32 | Middelbeek (2021)[37] | ELISA  (NeoScientific Co., Cambridge, Massachusetts) | Exercise Intervention:  Sprint Interval Training (SIT):6 sessions of 6 × 30 s all-out cycling sprints with 4 min recovery | VO₂ peak test, Indirect calorimetry, [Fluorine-18 Fluorodeoxyglucose Positron Emission Tomography/  Computed Tomography scanning for glucose uptake in skeletal muscle and adipose tissue, Body composition via MRI and BIA | 2 weeks |  |
|  |  |  | Moderate-Intensity Continuous Training (MIT):6 sessions of cycling at 60% VO₂ peak, increasing from 40 to 60 min |  |  |  |
| 33 | Morishima (2021)[38] | ELISA  (Not detailed) | Resistance exercise (leg extension, 5 sets of 10 reps at 70% 1RM) | sKl, Endothelin-1 levels, blood pressure, and Flow-Mediated Dilation ​via duplex-Doppler ultrasound​ | 60 minutes |  |
| 34 | Mostafidi (2016)[39] | ELISA  (Hangzhou Eastbiopharm Co., Ltd., China) | Exercise Intervention:  aerobic and anaerobic components  (running, drills, endurance training) | Plasma **α-**Klotho level | No follow-up |  |
| 35 | Rahimi (2018)[40] | ELISA  (IBL International GmbH, Germany) | Bruce test and a 12-week water aerobics program (3 sessions per week) | **α-**Klotho and IGF-I levels, cardiac hypertrophy indexes (Left Ventricular End-Diastolic Diameter Index, Left Ventricular Mass Index) | 12 weeks for chronic exercise + short-term assessment after acute exercise |  |
| 36 | Rangraz (2023) [114] | ELISA  (Demeditec, Kiel, Germany) | Aerobic exercise (running on a treadmill at 55%-70% max heart rate, 3 times per week for 6 weeks) | Serum levels of **α-**Klotho, Interleukin-10, Interleukin-1 and oxidative stress (Hydrogen peroxide via autoanalyzer) | 6 weeks |  |
| 37 | Saghiv (2017)[115] | ELISA  (IBL Co., Ltd., Japan) | aerobic exercise training (45 min, 4–5 sessions/week) for coronary artery disease patients | **α**-Klotho and Pro-BNP (brain natriuretic peptide) levels | 12 weeks |  |
| 38 | Saghiv (2017)[116] | ELISA  (Not detailed) | Chronic powerlifting resistance training (≥3 years) | **α**-Klotho and Insulin-like Growth Factor 1 | No longitudinal follow-up |  |
| 39 | Santos Rosa (2020)[44] | ELISA  (IBL Co., Ltd., Japan) | Exercise modality (master sprinters vs. endurance athletes vs. untrained controls) | Classification into groups based on self-reported lifelong training history and athletic participation | Cross-sectional study, no follow-up period |  |
| 40 | Shardell (2015)[45] | ELISA  (IBL Co., Ltd., Japan) | Lower-extremity physical performance | SPPB  Components:   - Balance assessment - Chair stand ability   Walking speed | Total 9 years  3-year follow-up: 2001–2003  6-year follow-up: 2004–2006  9-year follow-up: 2007–2009 |  |
|  |  |  | Cognitive Impairment | MMSE score |  |  |
|  |  |  | Physical Activity | - Moderate-to-High Activity: ≥3 hrs/week of moderate-to-intense exercise - Low Activity: ≥2 hrs/week of light exercise or 1–2 hrs/week of moderate exercise   Inactive: Sedentary, mostly sitting, some walking |  |  |
|  |  |  | Fall History | Number of falls in the past 12 months |  |  |
| 41 | Shardell (2020)[46] | ELISA  (IBL Co., Ltd., Japan) | Mobility disability | Based on self-reported difficulty.  Walking Disability: "A lot of difficulty" or inability to walk 1/4 mile.  Stair Climb Disability: "A lot of difficulty" or inability to climb 10 stairs | 6 years |  |
| 42 | Snigdha (2024)[47] | ELISA  (R&D Systems, Minneapolis, MN, USA) | Yoga-based lifestyle intervention (YBI) | BMI, blood biomarkers (HbA1c, LDL-C, CRP), cognitive function (Digit Symbol Substitution Test, TMT A/B), physical capability (gait speed, grip strength), and psychological/social well-being | 26 weeks (approximately 6 months)​ |  |
| 43 | Tan (2018)[48] | ELISA  (IBL Co., Ltd., Japan) | High-intensity Bruce protocol treadmill exercise | sKl, serum phosphate, blood glucose levels | 1-week post-exercise |  |
| 44 | Tetik Dündar (2023)[117] | ELISA  (Elabscience Biotechnology Inc., Houston, Texas) | Aerobic power test at low-medium altitude (2000 m) | Aerobic power test using the Åstrand-Rhyming bicycle ergometer test with progressively increasing workload | 48 hours |  |
| 45 | Valenzuela (2019)[50] | ELISA  (IBL Co., Ltd., Japan) | Physical performance | assessed by means of the 6MWT,  handgrip strength, and the Sit-to-Stand Test | 18 months |  |
|  | **Cognitive, Neuropsychiatric, and Psychological Health** | | | | |  |
| 46 | Ahmadi (2016)[51] | ELISA  [Immuno-Biological Laboratories (IBL), Co, Ltd, Japan] | Multiple sclerosis status: newly diagnosed relapsing-remitting patients (untreated), chronic patients (on interferon beta-1a), and healthy controls | Revised McDonald’s criteria, clinical history, and medical records | Cross-sectional study, no follow-up period |  |
| 47 | Barbosa (2017)[52] | ELISA  (R&D Systems, Minneapolis, MN, USA) | Bipolar disorder status: mania, remission, vs. healthy control | Diagnosis via Mini-International Neuropsychiatric Interview; manic and depressive symptom severity assessed by YMRS and HAM-D, respectively | Cross-sectional study, no follow-up period |  |
| 48 | Brombo (2018)[53] | ELISA  (IBL Co., Ltd., Japan) | Cognitive status classified into four groups: vascular dementia, late-onset Alzheimer’s disease, mild cognitive impairment, and cognitively normal controls | Assessed using neuropsychological testing (MMSE), functional evaluations (Basic ADL, Instrumental ADL), neuroimaging (CT/MRI), and diagnostic criteria (NINDS-AIREN for vascular dementia, NINCDS-ADRDA for Late-onset Alzheimer’s disease) | Cross-sectional study, no follow-up period |  |
| 49 | Cui (2024)[54] | ELISA  (Wuhan USCN Business, Wuhan, China) | Mild Cognitive Impairment (MCI) | MCI Diagnosis Criteria of DSM-IV  MoCA Scale  MMSE | No follow-up; data collected at a single point |  |
| 50 | Çelebi (2025)[55] | ELISA  (Elabscience Biotechnology Inc., Houston, Texas) | Executive Function and Selective Attention | Stroop Test | No follow-up; data collected at a single point |  |
|  |  |  | Memory and Visual Learning | Wechsler memory scale – visual production subtest |  |  |
| 51 | Ellidag (2016)[56] | ELISA  (YH Biosearch, Shanghai, China) | Multiple sclerosis status (relapsing-remitting patients vs. healthy controls) | Diagnosis confirmed using revised McDonald criteria; disability assessed via the Expanded Disability Status Scale (EDSS) and progression index (EDSS/disease duration) | Cross-sectional study, no follow-up period |  |
| 52 | Emir (2023)[57] | ELISA  (Elabscience Biotechnology Inc., Houston, Texas) | Bipolar disorder (manic episode) vs. healthy controls | Diagnosed using DSM-5 criteria; severity assessed using YMRS and HAM-D; plasma collected after overnight fasting | Cross-sectional study, no follow-up period |  |
| 53 | Gaitan (2021)[58] | ELISA  (R&D Systems, Minneapolis, MN, USA) | Cardiorespiratory Fitness Assessment | VO₂ peak Assessment: Graded treadmill test with 2.5% incline increases every 2 min until exhaustion. Measured via metabolic cart. | 26 weeks |  |
|  |  |  | Free-Living Physical Activity Assessment | Triaxial accelerometer worn for 7 days (≥10  hrs/day, ≥3  weekdays, 1  weekend) classified  as sedentary, light,  moderate, or vigorous |  |  |
|  |  |  | Cognitive Impairment | MMSE for global  cognition, California  Verbal Learning  Test-II for verbal  memory (Total &  Long Delay scores),  and Delis–Kaplan  Executive Function  System Color-Word  Interference for  executive function |  |  |
| 54 | Gao (2021)[59] | ELISA  (IBL Co., Ltd., Japan) | Major depressive disorder status (young/elderly; first-episode/recurrent) | Diagnosed using DSM-IV; symptom severity assessed using HAM-D | Cross-sectional study, no follow-up period |  |
| 55 | Ge (2024)[60] | ELISA  (IBL International Co., Ltd., Japan) | Memory | Consortium to Establish a Registry for Alzheimer’s Disease Word List | No follow-up, data obtained from NHANES (2011-2014) |  |
|  |  |  | Language Function and Executive Function | Animal Fluency Test |  |  |
|  |  |  | Processing Speed, Working Memory | Digit Symbol Substitution Test |  |  |
| 56 | Guo (2017)[61] | ELISA  (R&D Systems, Minneapolis, MN, USA) | Motor function | Hoehn and Yahr Staging Scale  Unified Multiple System Atrophy Rating Scale  Webster Scale | Cross-sectional study, no follow-up period |  |
|  |  |  | Non-motor symptoms | Non-Motor Symptoms Scale  Schwab and England ADL Scale |  |  |
|  |  |  | Cognitive function | MMSE |  |  |
| 57 | Kato (2024)[62] | ELISA  (IBL International Co., Ltd., Japan) | Cognitive Impairment | MoCa  MMSE | No follow-up; data collected at a single point |  |
| 58 | Kılıçaslan (2022)[63] | ELISA  (Elabscience Biotechnology Inc., Houston, Texas) | Schizophrenia diagnosis and symptom assessment | Diagnosis based on DSM-5; symptom severity assessed using the Positive and Negative Syndrome Scale and the Clinical Global Impression Scale | Cross-sectional study, no follow-up period |  |
| 59 | Kundu (2022)[64] | ELISA  (R&D Systems, Minneapolis, MN, USA) | Cognitive Impairment | MMSE | No follow-up; data collected at a single point |  |
|  |  |  | Dementia Status | Clinical Dementia Rating |  |  |
| 60 | Kuriyama (2017)[65] | ELISA  (IBL Co., Ltd., Japan) | Deep White Matter Lesions (DWML) | Fazekas scale (Grade)  Brain MRI FLAIR and T2 imaging.  T1-weighted Transverse images were acquired. Brain hemorrhages were assessed based on susceptibility-weighted imaging (SWI). | No follow-up; data collected at a single point. |  |
|  |  |  | Cognitive Function | MMSE |  |  |
| 61 | Linghui (2023)[66] | ELISA  (IBL International Co., Ltd., Japan) | Cognitive Function | Consortium to Establish a Registry for Alzheimer’s Disease  Animal Fluency Test  Digit Symbol Substitution Test | No follow-up; data obtained from NHANES (2011–2014) |  |
| 62 | Prather (2015)[67] | ELISA  (IBL Co., Ltd., Japan) | Chronic psychological stress | Stress assessed using Perceived Stress Scale; depressive symptoms via Inventory of Depressive Symptomatology | Cross-sectional study, no follow-up period |  |
| 63 | Sanz (2021)[68] | ELISA  (IBL Co., Ltd., Japan) | Dependence ADL | The Barthel Index | 6-month follow-up |  |
|  |  |  | Waist and Hip Circumference | Non-elastic anthropometric  Tape |  |  |
|  |  |  | Fat and Muscle Body Percentage | Bioelectrical impedance analyzer (Bodystat BIA  Quadscan 4000) |  |  |
|  |  |  | Physical Function | Jamar dynamometer for Hand Grip Strength  6MWT for gait speed  Berg Balance Scale for balance  SSPB |  |  |
|  |  |  | Cognitive Function | MoCa  Trail Making Test A  WAIS IV  MMSE |  |  |
|  |  |  | Frailty Status | Fried Frailty Index (FFI)  Tilburg Frailty Indicator (TFI)  Clinical Frailty Scale (CFS) |  |  |
|  |  |  | Fall History | Number of falls in the past 12 months |  |  |
| 64 | Sartorius (2019)[69] | ELISA  (IBL Co., Ltd., Japan) | Major depressive episode; antidepressant treatment (electroconvulsive therapy or pharmacotherapy) | Diagnosis via DSM-IV; severity via HDR-S 21; serum collected pre- and post-treatment | 4 weeks (pharmacotherapy); electroconvulsive therapy duration of (mean ~2 weeks) |  |
| 65 | Shardell (2016)[70] | ELISA  (IBL Co., Ltd., Japan) | Cognitive Function | MMSE and Trail-Making Test A & B administered at baseline, 3-year, and 6-year follow | Longitudinal study with 6-year follow-up. |  |
| 66 | Ushigusa (2016)[71] | ELISA  (IBL Co., Ltd., Japan) | Diagnosis of neuropsychiatric systemic lupus erythematosus, viral meningitis, multiple sclerosis, and neuromyelitis optica | American College of Rheumatology criteria, clinical symptoms, cerebrospinal fluid analysis, MRI , and single-photon emission computed tomography; cerebrospinal fluid cytokines measured using multiplex bead assay | No follow-up; cross-sectional (retrospective data from 2006–2015) |  |
| 67 | Vázquez- Lorente (2023)[118] | ELISA  (Demeditec, Kiel, Germany) | Exercise intervention:  The physical activity recommendation (PAR) group  HIIT group,  HIIT plus whole-body electromyostimulation (HIIT-  EMS) group. | The PAR group performed a concurrent training intervention combining aerobic and resistance training.The participants trained 3 days/ week for 12 weeks. The training volume was 150 min  The HIIT group performed a high-intensity interval program. The participants exercised 2 days/week for 12 weeks.  The HIIT-EMS group performed a training  program with the same structure as the HIIT, and received electrical stimulations. | 12-week randomized controlled trial |  |
|  |  |  | Memory function Immediate Memory (IM), Long-Term Memory (LTM), and Recognition | Wechsler Memory Scale-Third Edition (WMS-III) |  |  |
| 68 | Wu (2023)[73] | ELISA  (IBL International Co., Ltd., Japan) | Cognitive Function | Digit Symbol Substitution Test | No follow-up duration, data from the NHANES 2011–2014 |  |
| 69 | Wu (2024)[74] | ELISA  (IBL International Co., Ltd., Japan) | Cognitive Function | Memory: Consortium to Establish a Registry for Alzheimer’s Disease Word List (Immediate & Delayed Recall) – Assesses verbal learning.  Executive Function: Animal Fluency Test – Names as many animals in 1 min Processing Speed & Working Memory: Digit Symbol Substitution Test (WAIS-III) – 2 min symbol-number matching. | No follow-up duration, data from the NHANES 2011–2014 |  |
| 70 | Xiong (2020)[75] | ELISA  (Wuhan USCN Business, Wuhan, China) | Cognitive Function | Trail Making Test (TMT-A), Brief Assessment of Cognition in Schizophrenia (BACS), Continuous Performance Test (CPT-IP), Wechsler Memory Scale (WMS-III), Hopkins Verbal Learning Test-Revised-R, Brief Visual-Spatial Memory Test (BVMT-R), and Stroop Color-Word Test. | No follow-up period |  |
| 71 | Yalcin (2025)[76] | ELISA  (Bioassay Technology Laboratory, BT LAB) | Cognitive Function | MMSE | No follow-up period |  |
| 72 | Yokoyama (2017)[77] | ELISA (Immuno-Biological Laboratories, Takasaki, Japan) | Cognitive Function | Cortical gray matter volume | No follow-up period |  |
| 73 | Zhang (2023)[5] | ELISA (IBL International Co., Ltd., Japan) | Cognitive Function | Consortium to Establish a Registry for Alzheimer’s Disease Word List & Consortium to Establish a Registry for Alzheimer’s Disease-DR (word list learning and recall tests for verbal memory), Digit Symbol Substitution Test, Animal Fluency Test | No follow-up duration, data from the NHANES 2011–2014 |  |
| 74 | Zhang (2023)[6] | ELISA  Cloud-Clone Corp., Houston, TX, USA) | Mild Cognitive Impairment | MoCA | No follow-up period |  |
| 75 | Zhu (2018)[78] | ELISA  (IBL Co., Ltd., Japan) | Cognitive Function | MoCA and MMSE scores | prospective cohort (February–May 2017); long-term follow-up not specified |  |
|  | **Musculoskeletal Function, Aerobic Capacity** | | | | |  |
| 76 | Amaro-Gahete  (2019)[1] | ELISA  (Demeditec, Kiel, Germany) | LMI | DXA | Cross-sectional study, no follow-up period, data obtained between September and October in 2015 and 2016 |  |
|  |  |  | FMI |  |  |  |
|  |  |  | BMD |  |  |  |
|  |  |  | BMI | Weight and height measurement |  |  |
| 77 | Baldan  (2015)[80] | ELISA  (IBL Co., Ltd., Japan) | BMD | DXA | Between November 2011 and February 2012 |  |
|  |  |  | Hand grip strength | JAMAR hand-held isometric dynamometer |  |  |
| 78 | Chalhoub  (2016)[81] | ELISA  (IBL Co., Ltd., Japan) | BMD | DXA | Median of 5 years |  |
|  |  |  | BMI | Weight and height measurement |  |  |
|  |  |  | Physical Activity | Kcal per kg per week attributed to walking or climbing stairs |  |  |
|  |  |  | Gait Speed | Over 6 m expressed as meters per second |  |  |
|  |  |  | Muscle strength | Maximum grip strength attempt |  |  |
|  |  |  | ALM | Sum of lean mass in the arms and legs obtained by whole body DXA |  |  |
| 79 | Desbiens  (2018)[82] | ELISA  (IBL, Minneapolis, MN, USA) | Fractures | Patients’ clinical records and two reviewers | Median of 42 months |  |
|  |  |  | Arterial Stiffness Parameters | Aortic PWV (m/s) |  |  |
|  |  |  |  | Brachial PWV(m/s) |  |  |
|  |  |  |  | Aortic-brachial PWV ratio |  |  |
|  |  |  | Bone Mineral Parameters | Albumin (g/L)  Calcium (mmol/L)  Phosphate (mmol/L)  Parathyroid Hormone (ng/L)  Alkaline phosphatase Dickkopf-related Protein 1 (pg/mL)  Fibroblast Growth Factor 23 (RU/L)  Procollagen Type 1-N Terminal Propeptide (pg/mL)  Sclerostin (ng/mL)  Tartrate-resistant Acid Phosphatase 5b (U/L) |  |  |
| 80 | Desbiens  (2022)[83] | ELISA  (IBL, Minneapolis, MN, USA) | Fractures | C-terminal Fibroblast Growth Factor 23, Parathyroid Hormone, phosphate, and Calcium | Median of 70 months |  |
|  |  |  | BMD  (Calcaneal) | Calcaneal quantitative ultrasound |  |  |
| 81 | Ferreira  (2021)[84] | ELISA  (IBL, Minneapolis, MN, USA) | BMD | DXA | 1 year after renal transplantation |  |
| 82 | Ferreira (2022)[85] | ELISA  (IBL, Minneapolis, MN, USA) | Bone histomorphometric parameters | Cortical bone, trabecular bone,turnover, mineralization,  volume, Parathyroid Hormone, bone alkaline phosphatase, Fibroblast Growth Factor 23, and sclerostin | 12 months |  |
|  |  |  | Extraosseous calcifications | Agatston percentiles score |  |  |
| 83 | Fukasawa  (2014)[86] | ELISA  (IBL Co., Ltd., Japan) | Muscle mass indices:   - Abdominal muscle area (AMA) | CT | No follow-up duration |  |
|  |  |  | - Creatinine production rate (Cr-CKM) | Creatinine kinetic model |  |  |
| 84 | Gözükara (2019)[119] | ELISA  (IBL Co., Ltd., Japan) | Sarcopenia | Muscle Strength:   - Handgrip Strength - Knee   Flexion/Extension  Muscle Mass:   - Anthropometry (arm/calf circumference) - BIA   MRI/CT/DXA Muscle Performance  SPPB (balance, walking, endurance)  Gait Speed Test (6MWT)  Timed Up and Go (dynamic balance, fall risk) | Follow-up not specified |  |
| 85 | Han (2018)[120] | ELISA  (Wuhan USCN Business, Wuhan, China) | BMD | DXA | No follow-up period |  |
| 86 | Huang (2024)[89] | ELISA  (Elabscience Biotechnology Co., Ltd, China) | BMD | DXA | 1-month follow-up after BMD examination |  |
| 87 | Hughes-Austin (2020)[90] | ELISA  (IBL Co., Ltd., Japan) | Bone histomorphometric parameters | Parathyroid Hormone, Fibroblast Growth Factor 23, bone structure, formation, resorption, turnover, Dickkopf-related Protein 1, Sclerostin, Tartrate-resistant Acid Phosphatase 5b, bone-specific alkaline phosphatase | Began in 1997 followed through 2011  (for 14 years)  mean 8.4 years |  |
|  |  |  | BMD | DXA |  |  |
| 88 | Jiang (2023)[3] | ELISA  (IBL International Co., Ltd., Japan) | BMD | DXA | Began in 1999, analyzed three two-year cycles (2007–  2010 and 2013–2014) |  |
| 89 | Jiang(2024)[91] | ELISA  (CUSABIO Biotech, Newark, DE) | BMD | Quantitative Computed Tomography | January 2021 – June 2022 (≈1.5 years) |  |
| 90 | Katsaounis (2022)[92] | ELISA  (Cloud-Clone Corp Wuhan, China) | BMD | DXA | Not applicable |  |
| 91 | Kureya (2016)[93] | ELISA (Phoenix Pharmaceuticals, Burlingame,  CA, USA) | Lung function test | **Incremental exercise capacity** was assessed using a **symptom-limited incremental exercise test** on an **upright electromagnetically-braked cycle ergometer** (EM840, Siemens, Munich, Germany) | No follow-up |  |
|  |  |  | Cardiopulmonary exercise test |  |  |  |
|  |  |  | Skeletal muscle function |  |  |  |
|  |  |  | Irisin levels | ELISA (Immuno-Biological Laboratories, Gunma,  Japan) |  |  |
| 92 | Kuzmova (2021)[94] | ELISA  (Not detailed) | BMD | DXA | No follow-up; data collected at a single time point (July 2018 – July 2019) |  |
|  |  |  | Trabecular Bone Score | DXA |  |  |
| 93 | Lima (2019)[95] | ELISA  (IBL Co., Ltd., Japan) | Bone histomorphometric parameters | bone turnover, formation,  resorption, bone-specific alkaline phosphatase, Dickkopf-related Protein 1, Fibroblast Growth Factor 23, Parathyroid Hormone, sclerostin, TRAP-5b,1,25D | No follow-up; data collected at a single time point during bone biopsy |  |
| 94 | Marchelek-Myśliwiec (2018)[96] | ELISA  (Cusabio Biotech, Wuhan, China) | BMD | DXA | Not applicable |  |
| 95 | Martin-Gonzalez (2023)[97] | ELISA  (Elabscience Biotechnology Inc., Houston, Texas) | Body Composition | - BMI (kg/m^2^) - Abdominal circumference, cm | No follow-up duration |  |
| 96 | Matei (2022)[98] | ELISA  (Elabscience Biotechnology Inc., Houston, Texas) | Body composition parameters (total fat mass, trunk and  lower limbs fat mass, total and ALM) | DXA | No follow-up  Mean Post- Kidney Transplantation Duration: 58 months (long-term changes) |  |
|  |  |  | BMD |  |  |  |
|  |  |  | Bone Mineral Parameters | Parathyroid Hormone, Fibroblast Growth Factor 23, osteocalcin, Calcium, phosphate |  |  |
| 97 | Neves (2021)[99] | ELISA  (IBL Co., Ltd., Japan) | Exercise Intervention:  Dynamic resistance training (DRT) | BMD, which was assessed with DXA | 6 months |  |
|  |  |  | Isometric resistance training (IRT) |  |  |  |
|  |  |  | Hand Grip Strength | Average of three (trials using the contralateral arm to the arteriovenous fistula) measurements |  |  |
| 98 | Pedersen (2013)[100] | ELISA (Immuno-  Biological laboratories, IBL) | Estimated glomerular filtration rate (eGFR) | Calculated using the MDRD Study Equation | No follow-up duration |  |
|  |  |  | Serum FGF-23 concentrations | ELISA (Kainos Laboratories, Tokyo, Japan) |  |  |
|  |  |  | Body weight  Biomarkers of mineral metabolism |  |  |  |
| 99 | Ribeiro (2020)[101] | ELISA  (IBL Co., Ltd., Japan) | Fractures | Fracture occurrence based on clinical records or medical history | Every 3 months for 7 years (2010–2017) |  |
| 100 | Salamanna (2024)[102] | ELISA  (Bio-Techne, MN, USA) | Osteosarcopenia, osteopenia, sarcopenia | Interleukin-15, Dehydroepiandrosterone Sulfate -S,  Bone & muscle structure: Histology & immunohistochemistry (Osteoprotegerin, Receptor Activator of Nuclear Factor Kappa-B Ligand, Bone Morphogenetic Protein-2, Tumor Necrosis Factor-α, Collagen Type I Alpha 1 Chain),  Spontaneous osteoclastogenesis | No follow-up duration |  |
| 101 | Sawada (2024)[103] | ELISA  (R&D Systems, Minneapolis, MN, USA) | BMD | DXA | from January 2010 to January 2016 (6 years) |  |
| 102 | Semba (2016)[104] | ELISA  (IBL Co., Ltd., Japan) | Knee Extensor Strength | Measured with an isokinetic dynamometer (Kin-Com, 125 AP) at 60°/sec  Right knee tested unless contraindicated  Peak torque (N·m) averaged from the best 3 of 6 trials | 4 years  Assessment Timepoints: Baseline, 2-year, and 4-year follow-ups  Participants:  Visit 2 (Baseline): 2,775  Visit 4 (2-year): 2,401  Visit 6 (4-year): 2,105 |  |
|  |  |  | Hand Grip Strength | JAMAR hand-held isometric dynamometer |  |  |
|  |  |  | BMI | Weight and height measurement |  |  |
|  |  |  | Cognitive Impairment | MMSE score |  |  |
| 103 | Teng Lee (2023)[121] | ELISA  (IBL, Minneapolis, MN, USA) | BMD | DXA | No follow-up |  |
| 104 | Wang (2024)[106] | ELISA  (IBL International Co., Ltd., Japan) | Alveolar Bone Loss | Severe Periodontal Attachment Loss (>5 mm attachment loss) assessed by counting affected teeth in oral exams | No follow-up  Data obtained from NHANES (2009–2014) |  |
| 105 | Wei (2019)[107] | ELISA  (IBL Co., Ltd., Japan) | Cognitive Function | Global Cognition: Assessed with the MMSE  Verbal Memory: Evaluated using the Hopkins Verbal Learning Test-Revised-R (immediate and delayed recall)  Spatial Memory – Measured with the Brief Visuospatial Memory Test-Revised (BVMT-R)  Executive Function – Assessed using the Trail A Test  Verbal Fluency – Evaluated with the Verbal Fluency Test (VFT)  Cognitive impairment: ≥1.5 SD below norms in ≥2 domains | 24 months |  |
| 106 | Xie  (2024)[108] | ELISA  (IBL Co., Ltd., Japan) | Body Composition:  ALM | DXA | No follow-up duration |  |
|  |  |  | BMI | Weight and height measurement (kg/m^2^) |  |  |
| 107 | Yun (2020)[109] | ELISA  (IBL Co., Ltd., Japan) | Trabecular Bone Score | DXA | Average of 20 months |  |
|  |  |  | Frailty Assessment | Tilburg frailty indicator |  |  |
|  |  |  | Body composition | BIA |  |  |
|  |  |  | Handgrip Strength | Jamar plus  Digital Hand Dynamometer |  |  |
| 108 | Zhang (2023)[7] | ELISA  (IBL International Co., Ltd., Japan) | BMD | DXA | No follow-up  Data obtained from NHANES (2011–2016) |  |
|  |  |  | Physical Activity | MET score, Total MET minutes/week, categorized as 0 MET min/week, ≤600 MET min/week, 600–1200 MET min/week (meets), and ≥1200 MET min/week (exceeds) |  |  |
| 109 | Zheng (2018)[110] | ELISA  (Bio-Swamp Company, NY, USA) | BMD | DXA | No follow-up Data obtained from January 2015 and November 2015 |  |
|  |  |  | Bone mineral parameters | Calcium, Phosphorus, Parathyroid Hormone, Fibroblast Growth Factor 23, 1,25(OH)2VitD3, albumin |  |  |

**Abbreviations:** LMI: Lean Mass Index, FMI: Fat Mass Index, BMI: Body Mass Index, BMD: Bone Mass Density, DXA: Dual-energy X-ray Absorptiometry, ALM: Appendicular Lean Mass, PWV: Pulse Wave Velocity, NHANES: National Health and Nutrition Examination Survey, BIA: Bioelectrical Impedance Analysis, MET: Metabolic Equivalent of Task, ADL: Activity of Daily Living, MMSE: Mini-Mental State Examination, SPPB: Short Physical Performance Battery, 6MWT: 6-Minute Walk Test, FI: Frailty Index, MRI: Magnetic Resonance Imaging, CT: Computed Tomography, IPAQ: International Physical Activity Questionnaire, 1RM: One-Repetition Maximum, S-Klotho: Soluble Klotho, sKl: serum klotho, HIIT: High-intensity interval training, NINDS-AIREN: National Institute of Neurological Disorders and Stroke – Association Internationale pour la Recherche et l’Enseignement en Neurosciences, NINCDS-ADRDA: National Institute of Neurological and Communicative Disorders and Stroke – Alzheimer’s Disease and Related Disorders Association, DSM-5: Diagnostic and Statistical Manual of Mental Disorders, Fifth Edition, YMRS: Young Mania Rating Scale, HAM-D: Hamilton Depression Rating Scale

**Supplement Table S10**. Summary of the key findings in the included studies.

| **No** | **Study** | **Key findings** | **Study limitations & key points** |
| --- | --- | --- | --- |
|  | **Frailty and Multidimensional Geriatric Assessment** | | |
| **1** | **Branco (2023)[8]** | - Low serum α-Klotho levels were associated with peritoneal membrane fibrosis. - α-Klotho also predicted MACE occurrence and timing, making it a promising non-invasive biomarker for cardiovascular risk in PD patients. - Frailty score was associated with worse outcomes (both MACE and PD failure). | - α-Klotho was explored as a surrogate instead of invasive techniques such as peritoneal biopsy |
| **2** | **Chuang (2023)[9]** | - Elevated systemic immune-inflammation index (SII) was independently associated with a higher risk of incident frailty over a median follow-up of 2.5 years. - Participants in the highest SII tertile had a 69% increased risk of becoming frail compared to those in the lowest tertile. | - Residual confounding from unmeasured factors such as physical activity cannot be ruled out. |
| **3** | **Guan (2023)[10]** | - Participants with higher α-klotho levels (>785.5 pg/mL) had a lower prevalence of frailty. - The inverse relationship between α-klotho and frailty was stronger in individuals under 60 years than in those aged over 60 years. | - This study uses data from the NHANES cohort. - The study utilized the Physical Frailty Phenotype and the Frailty Index to assess frailty status, showing consistent findings across these two measures. |
| **4** | **Jiang (2023)[4]** | - Higher α-klotho levels were significantly associated with lower frailty risk. - The relationship was L-shaped; the protective effect plateaued at 785.7 pg/ml. - Relationships between α-klotho and frailty remained significant when adjusted for other factors. | - This study used data from the NHANES cohort. - A comprehensive 49-item Frailty Index was used. - The relationship between α-klotho levels and frailty was visualized with Restricted Cubic Spline. |
| **5** | **Koyama (2015)[11]** | - Patients with osteoporosis had significantly lower α-klotho levels than those without osteoporosis. | - Potential confounding risk factors for osteoporosis were not analyzed thoroughly. |
| **6** | **Li (2023)[12]** | - There was a U-shaped relationship between serum Klotho levels and phenotypic age acceleration in US adults aged 40–79. - The U-shaped association remained significant after adjusting for a wide range of covariates | - The study used phenotypic age acceleration, which is based on 9 biomarkers. |
| **7** | **Longo (2022)[13]** | - Before the physical distancing period, light physical activity positively correlated with α-klotho levels. This correlation was lost after six months of physical distancing. | - This study included a small population, which may limit its statistical power. |
| **8** | **Luo (2024)[14]** | - Serum α-klotho levels showed a U-shaped relationship with all-cause and cancer-related mortality in frail adults, meaning both low and high levels were associated with higher risk. | - This study uses data from the NHANES cohort. |
| **9** | **Polat (2020)[15]** | - No significant correlation between frailty status and α-klotho level was shown. - There was a weak positive correlation between α-klotho levels and MMSE scores. - Correlations between α-klotho levels and frailty components such as grip strength and gait speed were not significant. | - Frailty was assessed with the Turkish version of the FRAIL scale.   This study also investigated the relationship between inflammatory biomarkers and α-klotho levels. |
| **10** | **Sagi (2020)[16]** | - α-Klotho levels were significantly lower in hemodialysis patients compared to healthy controls - α-Klotho levels did not correlate with pulse wave velocity or augmentation index, two markers of vascular stiffness. | - This study found no link between α-Klotho and vascular stiffness, whereas other literature suggests potential cardiovascular protective effects or links to vascular calcification. The control population is not matched and is small-sized. |
| **11** | **Semba (2012)[17]** | - Grip strength was positively associated with α-klotho levels in individuals with plasma α-klotho below 681 pg/mL, above this threshold, further increases in α-klotho were not associated with greater grip strength. |  |
| **12** | **Shardell (2019)[18]** | - Higher α-klotho was significantly associated with a 54 percent lower odds of frailty versus robustness per ln(α-klotho) (in pg/mL) after adjustment for covariates. However, each ln(α-klotho) (in pg/mL) was only associated with 6 percent lower odds of pre-frailty versus robustness, which was not statistically significant. - α-Klotho levels are associated with frailty components such as weight loss, exhaustion, sedentariness, weakness, and slowness. | - This study used data from InCHIANTI, a cohort comprising Italian adults. |
| **13** | **Yuan (2024)[19]** | - A significant inverse linear relationship between α-klotho levels and frailty risk was shown. Higher α-klotho levels were associated with lower frailty prevalence. - The protective effect of α-klotho was most pronounced in patients with a BMI higher than 25 kg/m² and patients under 65 years. | - This study uses data from the NHANES cohort. |
| **14** | **Żelaźniewicz (2022)[20]** | - No relationship between α-klotho levels and lifestyle-related risk factors, such as lack of physical activity or self-assessed psychological stress were shown | - This study also explores the relationship between α-klotho and cardiometabolic health parameters. |
| **15** | **Zhang (2022)[21]** | - A higher Dietary Inflammatory Index (DII) score was significantly associated with lower serum Klotho concentrations. - Participants in higher DII quartiles (especially Q3 and Q4) had significantly lower Klotho levels than those in the lowest quartile (Q1), after adjusting for confounders. | - Diet was assessed using a single 24-hour recall, which may not accurately reflect habitual dietary intake or account for day-to-day variability. |
|  | **Physical Function, Exercise, and Mobility Interventions** | | |
| **16** | **Aczel (2023)[22]** | - No significant correlation was found between oxygen consumption and α-klotho levels in either trained or sedentary individuals. - Higher grip strength was positively associated with α-klotho levels in the trained group | - α-Klotho promoter methylation was investigated to interpret epigenetic differences between populations. |
| **17** | **Amaro-Gahete Exercise (2019)[111]** | - All exercise training modalities induced an increase in the plasma α-klotho levels. - Positive association between changes in LMI and changes in the α-klotho levels, and a negative association between changes in fat mass outcomes and changes in the α-klotho levels were reported. | - This study employed RCT design and includes different training interventions, which is an advantage. - Changes in body composition parameters following a 12-week intervention were investigated. For long-term implications on the α-klotho-exercise relationship, studies with longer follow-up should be conducted. - Whole-body electromyostimulation, a training modality that is increasingly becoming popular in scientific studies, is also employed. - Participants in interventional groups had lower plasma klotho at baseline. |
| **18** | **Amaro-Gahete (2019)[2]** | - Moderate-to-vigorous physical activity (MVPA), hand grip strength, and VO₂max showed significant positive associations with α-Klotho plasma levels. | - The associations may be highly dependent on lean mass, which is closely related to both muscular strength and α-Klotho levels. |
| **19** | **Amaro-Gahete (2024)[24]** | - A 24-week supervised concurrent exercise training did not result in significant changes in α-klotho levels across different intensity groups. - No significant correlations were found between changes in α-klotho levels and changes in BMI, FMI, visceral adipose tissue, or LMI. | - This study investigates a young and healthy population (18-25) who already had normal α-klotho levels, which may be attributable to a lack of change in α-klotho levels. |
| **20** | **Arroyo (2023)[25]** | - α-Klotho was significantly lower in the 50-65 and 65 ≥ years groups compared to the 20-34 year group. - No significant difference in α-klotho levels was observed between low and high physical performers. - α-Klotho levels were not correlated with appendicular lean mass. - In the physical function domain, α-klotho was associated with the distance walked in the 6MWT; however, no association was shown for usual gait speed, STS, and grip strength. | - The relationship between α-klotho and physical function in a healthy cohort was investigated. - This study presents four age intervals, each consisting of 20 participants. Small population size is a limitation, given the physiological variability of α-klotho levels. |
| **21** | **Boeselt (2017)[26]** | - α-Klotho levels did not change significantly over the course of a 6-month high-intensity exercise training program. - Patients in the training group showed significant improvements in 6MWT distance, muscle mass, and quality of life. However, these benefits did not result in meaningful changes in α-klotho levels. | - Varying degrees of COPD severity may have influenced the α-klotho levels. |
| **22** | **Chen (2024)[27]** | - α-Klotho levels are significantly lower in individuals with maximal overweight, non-obese to obese transitions, and stable obesity compared to those maintaining normal weight within the ten years. - Participants moving from non-obese to obese had an average decrease of 39.16 pg/ml in α-klotho levels; those maintaining obesity had a decrease of 34.55 pg/ml. | - This study uses data from the NHANES cohort. - It was shown that α-klotho levels decreased with age, emphasizing its role as an aging biomarker. |
| **23** | **Collins (2023)[28]** | - α-Klotho concentration significantly increased from baseline to 6 months in participants with ≥10% weight loss. - Changes in α-klotho concentration at 6 months were inversely associated with weight, BMI, fat mass, and waist circumference. - Participants with less than 5% weight loss showed no significant change in α-Klotho levels at either 6 or 12 months. | - Initially, 383 participants were recruited for the study; however, 152 participants had complete α-klotho measurements, which potentially reduced statistical power. - Only participants with ≥10% weight loss (responders) or <5% weight loss (non-responders) were included, excluding those with moderate weight loss. |
| **24** | **Correa (2021)[29]** | - After 6 months of RT and RT + blood flow restriction, α-Klotho levels increased significantly compared to the control group. - The RT+Blood Flow Restriction group exhibited the greatest increase in α-Klotho levels. |  |
| **25** | **Crasto (2012)[30]** | - Older adults with lower α-klotho levels had a higher prevalence of disability in ADL. - When adjusted for confounding factors, higher α-klotho levels were independently associated with a lower risk of ADL disability.   Low α-klotho levels were associated with poor physical performance and cognitive decline. | - This study analyzed the older Italian participants from the InCHIANTI cohort. - ADL disabilities were reported as the participant stated the need for help with daily activities. |
| **26** | **Ercan (2022)[31]** | - Both rheumatoid arthritis patients and healthy controls showed a significant increase in α-klotho levels after a single session of aerobic exercise. | - Only patients with low disease activity were included in this study. - This study evaluated the acute effects of aerobic exercise, which does not interpret the longitudinal effects of exercise. |
| **27** | **Fakhrpour (2020)[32]** | - A 16-week combined aerobic and resistance exercise program led to a significant increase in α-klotho levels in the exercise group, whereas α-klotho levels significantly decreased in the control group. |  |
| **28** | **Ghadamyari (2024)[112]** | Both endurance and resistance exercise programs led to a significant increase in serum α-klotho levels after 8 weeks. The endurance exercise group showed the greatest increase in α-klotho levels compared to resistance training and the control group. | - Longer interventions are needed to investigate the effect of sustained exercise on metabolic parameters. |
| **29** | **Girginer (2019)[113]** | - Elite master athletes and recreational athletes had significantly higher plasma α-klotho levels compared to sedentary control groups. - No significant difference in α-klotho levels was found between the two exercise groups, yet both were higher than in the sedentary group. |  |
| **30** | **Iturriaga (2021)[35]** | - A single session of cardiorespiratory exercise led to a significant increase in α-klotho levels immediately post-exercise. - Immediately post-exercise, α-klotho levels decreased slightly after strength training, though this drop was not statistically significant. However, at 24 and 48 hours post-exercise interval, α-klotho levels significantly increased compared to baseline. | - The study included physically active men (ages 18-55), limiting generalizability to the general population. |
| **31** | **Matsubara (2014)[36]** | - α-Klotho concentrations significantly increased following a 12-week moderate-intensity aerobic exercise program - Despite the increase in α-klotho, there was no significant correlation between α-klotho levels and improvements in ventilatory threshold or oxygen consumption. | - The limited number of participants in this study limits the statistical power. |
| **32** | **Middelbeek (2021)[37]** | A two-week moderate-intensity exercise program significantly increased α-klotho levels. However, sprint interval training did not alter α-klotho levels. | - This study included middle-aged men, limiting the generalizability to the general population. - Long-term effects of different exercise intensities on α-klotho levels remain unknown. |
| **33** | **Morishima (2021)[38]** | - A single bout of resistance exercise significantly increased α-klotho levels. The increase in α-klotho was observed immediately post-exercise but returned to baseline within 30–60 minutes. | - This study included only 12 young, untrained male participants, limiting generalizability to the general population. - Long-term effects of exercise on α-klotho are not investigated in this study. |
| **34** | **Mostafidi (2016)[39]** | - Trained football players had significantly higher α-klotho levels compared to non-athletes. | - This study included a young male adult population, which may limit the generalizability. |
| **35** | **Rahimi (2018)[40]** | - Acute exercise significantly increased α-klotho levels in both athletes and non-athletes. - Chronic exercise also increased α-klotho levels, but only in the athlete group, while non-athletes showed a return to baseline levels after 72 hours post-exercise. | - The population of this study limits the statistical power and generalizability of the results. |
| **36** | **Rangraz (2023)[114]** | - A six-week moderate-intensity aerobic exercise program significantly increased serum α-klotho levels. | - The population of this study limits the statistical power and generalizability.   The long-term effects of aerobic exercise on α-klotho are not investigated. |
| **37** | **Saghiv (2017)[115]** | - A 12-week supervised aerobic exercise program significantly increased α-klotho levels.   The exercise group showed an increase in α-Klotho from 770.49 pg/ml to 863.39 pg/ml, while no significant change occurred in the control group. |  |
| **38** | **Saghiv (2017)[116]** | - No significant difference in α-klotho levels was observed between trained powerlifters and untrained individuals. |  |
| **39** | **Santos Rosa (2020)[44]** | - Master athletes had better redox balance, inflammatory profiles, and aging biomarkers than untrained controls. - Sprinters showed higher antioxidant capacity, lower oxidative damage, higher α-klotho and irisin, lower FGF-23, longer telomeres, and stronger anti-inflammatory cytokine ratios. - Endurance runners had higher NOx, SOD, and interleukin-15 and lower ADMA. - Sprinters had a superior aging biomarker profile; endurance runners showed better cardiovascular markers | - A small, selective sample of elite male master athletes limits generalizability. - Cross-sectional design prevents causal inference. - Potential confounders (e.g., diet, stress, recent training) were not fully controlled.   Despite this, it is among the first to compare sprint vs. endurance training on aging biomarkers, highlighting sprint training’s potential benefits for aging, oxidative stress, and inflammation. |
| **40** | **Shardell (2015)[45]** | - α-Klotho levels are correlated with SPPB, even when adjusted to other covariates. - Higher α-klotho levels were significantly associated with better lower-extremity physical performance, independent of vitamin D levels and other covariates. - Positive associations were shown for balance and walking speed, but not for STS. | - This study used data from InCHIANTI, a cohort comprising Italian adults.   The hypothesis that α-klotho mediates vitamin D is not supported in this study. |
| **41** | **Shardell (2020)[46]** | - Participants with higher α-klotho levels had significantly lower rates of walking disability. - The association remained significant even after adjusting for confounding factors. | - Mobility disability was assessed using self-reports rather than objective physical performance measures. |
| **42** | **Snigdha (2024)[47]** | - The 26-week yoga-based intervention is shown to increase α-klotho levels. - Yoga was protective against frailty. | - This study assesses yoga’s impact using a multidimensional healthy aging phenotype approach, covering physiological, metabolic, cognitive, psychological, and social well-being markers simultaneously. |
| **43** | **Tan (2018)[48]** | - Acute exercise significantly affects klotho levels in healthy adults. - α-Klotho levels peaked immediately post-exercise but returned to baseline within 30 minutes. | - The population of this study limits its statistical power and generalizability. |
| **44** | **Tetik Dündar (2023)[117]** | - Acute exercise and aerobic power tests increase the α-klotho levels. α-Klotho levels increased by 332.8% 48 hours after the intervention. | - This study interprets the acute effects of exercise on α-klotho; the long-term effect remains unknown. |
| **45** | **Valenzuela (2019)[50]** | - Patients with lower α-klotho had a higher risk of low performance in grip strength, 6MWT, and STS.   α-Klotho levels were not significantly associated with mortality. However, physical performance was a better predictor of mortality compared to α-klotho levels. | - This study contained a small number of participants. Furthermore, only male patients were analyzed due to the small number of female participants, which makes the results non-generalizable.   The lack of time to death makes the Kaplan-Meier survival analysis improbable. |
|  | **Cognitive, Neuropsychiatric, and Psychological Health** | | |
| **46** | **Ahmadi (2016)[51]** | - Patients with relapsing–remitting multiple sclerosis of >3 years duration under interferon beta-1a treatment had significantly higher serum α-Klotho concentrations than healthy controls. - In contrast, newly diagnosed, treatment-naive relapsing–remitting multiple sclerosis patients had comparable serum α-Klotho levels | - The small-sized study design limits the generalizability and statistical power. |
| **47** | **Barbosa (2017)[52]** | - Patients with bipolar disorder, both in mania and remission, had significantly higher α-klotho than healthy controls. - α-Klotho levels did not correlate with manic or depressive symptom scores, comorbidities, or use of lithium, anticonvulsants, or antipsychotics. | - Bipolar patients had higher rates of hypertension, diabetes, and hypothyroidism, which may influence α-Klotho levels, yet these were not fully controlled for in the analysis. |
| **48** | **Brombo (2017)[53]** | - Individuals in the lowest α-Klotho tertile had a significantly higher risk of vascular dementia. - α-Klotho levels did not differ significantly between patients with late-onset Alzheimer’s Disease and controls | Overlap between vascular dementia and late-onset Alzheimer’s disease may have been underestimated, particularly in older adults. |
| **49** | **Cui (2024)[54]** | - Patients with MCI had significantly lower serum α-klotho levels compared to healthy controls. - Multivariate logistic regression analysis and Receiver Operating Characteristic analysis identified high α-Klotho levels as a protective factor against MCI. | - In this study, patients were not stratified patients based on MCI subtypes. |
| **50** | **Çelebi (2025)[55]** | - High α-klotho levels were the predictors of worse Wechsler 40-min test scores, implying a link with delayed visual memory. - α-Klotho was a predictor of Stroop test color-word reading time, suggesting a relationship with attention and processing speed​. | - Only the Stroop test and Wechsler visual memory scale were used, which does not include other domains of cognitive dysfunction. |
| **51** | **Ellidağ (2016)[56]** | - α-Klotho levels were significantly elevated in relapsing-remitting multiple sclerosis patients compared to healthy controls | - There was no correlation analysis with MRI, lesion load, or other clinical measures beyond the Extended Disability Status Scale and disease duration. |
| **52** | **Emir (2023)[57]** | - α-Klotho levels were significantly elevated in male patients with bipolar disorder during manic episodes compared to healthy controls. - α-Klotho levels were not correlated with clinical severity measures such as the Young Mania Rating Scale or Hamilton Depression Rating Scale. | - The patient group was limited to male forensic psychiatric inpatients, potentially reducing generalizability to the broader bipolar population. |
| **53** | **Gaitan (2021)[58]** | - After 26 weeks of moderate-intensity aerobic exercise, there was no significant change in klotho levels compared to baseline. Baseline α-klotho levels were positively correlated with baseline executive function. However, despite significant improvements in executive function and verbal memory following aerobic exercise, no correlation with the α-klotho levels were demonstrated. | - Only 23 participants were included in this study, which limits the statistical power. - This study only assessed executive function and verbal memory in the cognitive function domain. |
| **54** | **Gao (2021)[59]** | - Elderly patients with first-episode major depressive disorder had significantly lower plasma α-klotho levels compared to age-matched healthy controls. | - The treatment history of major depressive disorder patients was incompletely recorded, especially regarding antidepressant use. |
| **55** | **Ge (2024)[60]** | - Lower α-klotho levels were associated significantly with lower z-scores for the DSST and global cognition compared to those with higher α-klotho levels. - No significant relationship was observed between continuous α-klotho levels and cognition; only the lowest levels of α-klotho were associated with cognitive impairment. - Women in the lowest quartile of α-klotho had increased risk of cognitive impairment compared to the highest. Whereas, such risk was not observed for men. | - This study uses data from the NHANES cohort. - The study assessed only global cognition and processing speed and did not include other aspects of cognition. |
| **56** | **Guo (2017)[61]** | - α-Klotho levels were significantly lower in Multiple System Atrophy patients compared to healthy controls and Parkinson’s disease patients. - ROC curve analysis showed that serum α-klotho had predictive value for Multiple System Atrophy outcomes, particularly in female patients. | - Potential confounders were not comprehensively explored. |
| **57** | **Kato (2024)[62]** | - There is no significant independent correlation between α-klotho levels and MoCA or MMSE scores in both unadjusted and multivariate linear regression analyses. |  |
| **58** | **Kılıçarslan (2022)[63]** | Klotho levels were significantly higher in male patients with schizophrenia compared to healthy male controls, even after adjusting for age and BMI | - Only 40 male forensic psychiatric patients were included. The narrow demographic limits generalizability. |
| **59** | **Kundu (2022)[64]** | - Higher α-klotho levels in both serum and CSF were strongly correlated with better MMSE scores. - High serum and CSF α-klotho levels are also negatively correlated with Clinical Dementia Rating score, which indicates a protective effect against cognitive decline. | - A notable number of participants scored the highest points in the MMSE, which may limit the sensitivity of stratification of cognitive status. - CSF α-klotho measurement is considerably novel in this study. |
| **60** | **Kuriyama (2017)[65]** | - Higher α-klotho levels correlated positively with cognitive test scores; there is a significant association with MMSE and AFT. Although presented with a positive trend, there is no significant association with the Vegetable word fluency test. | - This study investigates the relationship between α-klotho levels and deep white matter lesions, which is novel for the assessment of cognitive status. |
| **61** | **Linghui (2023)[66]** | - The highest quartile of α-klotho was significantly associated with higher cognitive scores in the CERAD Score for the immediate and delayed memory domain and the DSST Score for processing speed compared to those in the lowest quartile. - The association between higher α-klotho levels and better cognitive performance remained significant after adjusting for confounding factors. |  |
| **62** | **Prather (2015)[67]** | - Women experiencing chronic psychological stress (caregivers of children with autism) had 12% lower serum α-klotho levels than age-matched low-stress controls. - Lower α-klotho levels were associated with higher depressive symptoms | - Participants were premenopausal, highly educated women in the San Francisco Bay Area. Results may not generalize to the population. |
| **63** | **Sanz (2021)[68]** | - No significant associations were found between α-klotho levels and measures of physical fitness such as Hand grip strength, 6MWT, Gait speed, Berg Balance Scale.​ - Lower α-klotho levels were significantly associated with reduced processing speed. | - This study included nursing home residents, primarily women (*70.9% female*), potentially limiting generalizability to other populations. |
| **64** | **Sartorius (2019)[69]** | - α-Klotho levels were not significantly different between patients with major depressive episodes and healthy controls | - Many patients had previous failed antidepressant trials, which may have influenced baseline α-klotho levels, although not clinically effectively. |
| **65** | **Shardell (2016)[70]** | - Higher concentrations of plasma α-klotho were associated with better MMSE scores at the 3-year follow-up. - From enrolment to year 3, higher α-klotho was linked to a 0.75-point smaller average MMSE decline, but no significant association was observed from year 3 to year 6 | - Missing follow-up data due to non-response or mortality might have introduced bias, although the authors used inverse-probability weighting to mitigate this. |
| **66** | **Ushigusa (2016)[71]** | - Cerebrospinal fluid α-klotho levels were significantly lower in patients with neuropsychiatric systemic lupus erythematosus compared to patients with non-neuropsychiatric systemic lupus erythematosus and viral meningitis. | - The cohort included a range of neuropsychiatric symptoms, which may have different underlying mechanisms, complicating the interpretation of α-Klotho’s role. |
| **67** | **Vázquez-Lorente (2024)[118]** | - Higher α-klotho levels were significantly correlated with greater BMD at the lumbar spine and total hip. Participants in the highest quartile of α-klotho had a 23% lower risk of osteoporosis compared to those in the lowest quartile. | - In this study, high α-klotho in hormone replacement therapy users was observed; however, it did not stratify analyses by therapy duration or type, which could have a confounding effect on α-klotho and BMD association. |
| **68** | **Wu (2023)[73]** | - α-Klotho concentrations were positively associated with cognitive performance and DSST scores. - Mendelian randomization analysis showed no significant relationship between genetically predicted α-klotho levels and any type of dementia. | - The Mendelian randomization analysis only evaluated linear relationships, whereas the observational study suggested a nonlinear relationship between α-klotho and cognitive function. |
| **69** | **Wu (2024)[74]** | - Higher serum α-klotho levels were significantly associated with better cognitive performance in the AFT and the DSST. - α-Klotho levels showed no significant association with scores on the Immediate Recall Test or Delayed Recall Test. | - This study uses data from the NHANES cohort. |
| **70** | **Xiong (2020)[75]** | - α-Klotho levels were positively correlated with cognitive functions, specifically attention, working memory, verbal memory, executive function, and composite cognitive score. The strongest correlation was shown for executive function. | - Selected patients were in an acute exacerbation phase without antipsychotic medication for over three months, which may have influenced cognitive performance. |
| **71** | **Yalcin (2025)[76]** | - A significant positive correlation was shown between α-klotho levels and MMSE scores. - The analysis demonstrated that α-klotho has a sensitivity of 90% and a specificity of 65% for predicting Parkinson’s disease. | - In this study, different types of dementia in Parkinson’s Disease patients were not distinguished, which may influence the observed correlation with α-klotho levels. |
| **72** | **Yokoyama (2017)[77]** | - Higher serum klotho levels are associated with greater gray matter volume - Higher klotho levels predict stronger intrinsic functional connectivity in brain regions vulnerable to aging and Alzheimer’s disease. | - No direct cognitive performance measurements were analyzed in relation to klotho levels in this study, despite implications for cognitive resilience. |
| **73** | **Zhang (2023)[5]** | - Patients with type 2 diabetes and MCI had significantly lower serum α-Klotho levels than those without MCI. - Multivariate logistic regression showed that lower α-klotho levels were independently associated with MCI. | - The MoCA was the only cognitive test used, which may limit the assessment of cognitive status. |
| **74** | **Zhang (2023)[6]** | - No significant linear relationship was observed between α-klotho and cognitive function. - A nonlinear association was detected between α-klotho and DSST scores, implying that the cognitive benefits of α-klotho plateau at high levels. | - This study uses data from the NHANES cohort. |
| **75** | **Zhu (2018)[78]** | - α-Klotho levels showed a positive correlation with MoCA scores. - However, there was no significant correlation between α-klotho levels and MMSE scores.   The analysis showed that α-klotho had poor sensitivity and specificity for diagnosing mild cognitive decline in hemodialysis patients. | - This study included a small population of 58 hemodialysis patients (29 with mild cognitive decline, 29 with normal cognitive status), which may limit the statistical power. |
|  | **Musculoskeletal Function, Aerobic Capacity** | | |
| **76** | **Amaro-Gahete (2019)[1]** | - There was a strong positive association between LMI and α-klotho levels, which persisted after controlling for demographics as well as after controlling for FMI. - Significantly positive associations of BMI and BMD were also found with α-klotho levels, which disappeared after controlling for LMI. | - The population of this study is limited to middle-aged sedentary individuals; for generalizable results, more diverse populations should be investigated. |
| **77** | **Baldan (2015)[80]** | - Beta thalassemia patients had significantly lower α-klotho levels than healthy controls. - A weak but significant correlation between α-klotho and grip strength was found below a threshold value, which suggests a saturation effect. - α-Klotho levels were lower in the patients with osteopenia hip bones. Furthermore, a history of fragility-associated fracture was significantly associated with lower α-klotho levels. | - No control group for BMD and muscle strength was used in this study. - This study was conducted with a population that had a limited age range and racial profile, which reduces the generalizability. |
| **78** | **Chalhoub (2016)[81]** | - Baseline femoral neck and lumbar spine BMD and yearly BMD loss were similar across different α-klotho levels. Moreover, linear regression between baseline BMD and α-klotho levels showed no significant association. - No significant association between α-klotho levels and risk of nonspine, hip, and vertebral fractures was shown. | - Although α-klotho levels were analyzed within quartiles, lower α-klotho quartiles did not indicate true α-klotho deficiency, as participants had levels within normal reference ranges. Patients with disabilities in ADL and poor physical function were excluded from the study. |
| **79** | **Desbiens (2018)[82]** | - Higher FGF23 levels were significantly associated with increased fracture incidence, even after adjusting for other factors. - α-Klotho levels correlated positively with bone formation markers, yet it was not associated with bone resorption markers.   No significant association between α-klotho levels and fracture risk was shown. | This study offers a comprehensive view of bone metabolism and vascular health by measuring the multiple osteocyte markers. |
| **80** | **Desbiens (2022)[83]** | - Higher and lower α-klotho levels were both associated with increased fracture risk, with the lowest risk at 742 pg/mL, demonstrating a U-shaped relationship with fracture risk in CKD patients. However, this association weakened after adjusting for PTH, phosphate, and BMD. - A U-shaped association was also demonstrated in non-CKD patients. - cFGF23 (C-terminal FGF23) was linearly associated with increased fracture risk in both CKD and non-CKD participants. | - BMD was measured using calcaneal quantitative ultrasound instead of the standard DXA, which may limit comparability with other studies​. |
| **81** | **Ferreira (2021)[84]** | - DXA of the total femur and femoral neck showed the strongest correlation with bone volume measured by bone biopsy. - Higher α-klotho levels are shown to be correlated with increased BMD and better femoral neck T-scores. | - The observed positive association between BMD and α-klotho/sclerostin levels may reflect their roles in bone homeostasis, offering insight into potential pathophysiological mechanisms. However, the lack of correlation between these biomarkers and bone biopsy findings suggests the need for further mechanistic studies. |
| **82** | **Ferreira (2022)[85]** | - After the kidney transplantation, a large decrease in the number of patients with high-remodeling disease and a significant increase in patients with low-remodeling disease was shown. - Patients who had experienced decreases in bone remodeling categories had higher α-klotho levels after 1 year of transplantation. | - The 1-year follow-up may be too short to detect long-term changes in vascular outcomes or fracture rates. |
| **83** | **Fukasawa (2014)[86]** | - The study did not find a significant association between α-klotho and muscle mass/muscle mass indices or FGF23 levels in hemodialysis patients. | - The optimal value of nPCR in maintenance hemodialysis patients is recommended to be 1.2 (g/kg per day) in the K/DOQI guideline. The spectrum of nPCR in this study population is relatively low, and this lower spectrum (lower dietary protein intake) may affect the results of muscle mass. |
| **84** | **Gözükara (2019)[119]** | - Sarcopenic patients had significantly lower α-klotho levels compared to non-sarcopenic individuals. - Higher α-klotho levels were associated with better muscle strength and physical performance. - Physical activity was shown to increase α-klotho levels. |  |
| **85** | **Han (2018)[120]** | - α-Klotho levels and lumbar spine BMD were significantly associated, even after adjusting for confounders.   Although α-klotho levels decreased progressively from the normal BMD group to the osteoporosis group, binary logistic regression showed no significant independent association between α-klotho levels and osteoporosis after adjusting for confounders. | - Only lumbar spine BMD was measured; hip or femoral neck BMD was not assessed, which may cause site-specific bias in findings. |
| **86** | **Huang (2023)[89]** | - α-Klotho levels were positively correlated with BMD at the lumbar spine and femoral neck. - Binary logistic regression identified low α-klotho levels as an independent risk factor for low BMD | - This study does not present data regarding bone loss over time. |
| **87** | **Hughes-Austin (2020)[90]** | - Higher α-klotho levels were strongly associated with low-turnover bone disease. - Without considering BMD, individuals classified as having low turnover by biomarkers had a similar fracture risk to those with non-low turnover | This study highlights that certain biomarkers together form a highly specific panel for identifying low bone turnover, which could guide treatment decisions in CKD patients without the need for invasive bone biopsies. |
| **88** | **Jiang (2023)[3]** | - Higher α-klotho levels are associated with reduced osteoporosis risk. Moreover, - Risk of osteoporosis significantly decreased when α-klotho levels are above 824.09 pg/mL, as demonstrated by the restricted cubic spline analysis​. | - This study uses data from the large-scale cross-sectional NHANES cohort, which represents the US population - In this study, the lack of key bone markers such as vitamin D and PTH limits the possibility of analyzing whether higher α-klotho levels are independently associated with low osteoporosis risk. |
| **89** | **Jiang (2024)[91]** | - α-Klotho levels were lower in patients with osteoporotic fractures; however, no correlation between α-klotho levels and fracture incidence was shown. - The AA genotype of the α-Klotho G395-A gene was significantly more prevalent in the fracture group compared to the control group. Logistic regression analysis showed that the AA genotype was an independent risk factor for osteoporotic fractures. | - BMD was measured using Quantitative Computed - Tomography, which may limit comparability with other DXA-based osteoporosis studies. |
| **90** | **Katsaounis (2022)[92]** | - No significant correlation between α-klotho levels and BMD at the lumbar spine or femoral neck in the cirrhotic patient population was shown. | - α-Klotho measurement lacks a standardized protocol; results are not readily comparable to studies using different assay units (pg/ml vs. ng/ml).​ |
| **91** | **Kureya (2016)[93]** | - Soluble α-Klotho levels were significantly lower in smokers with COPD compared to both non-smokers and smokers without COPD. - Interestingly, smokers without COPD had higher soluble α-Klotho levels than non-smokers, potentially indicating a compensatory response to smoking-induced stress. | - The study did not include CT-based emphysema scores, which could have strengthened correlations with lung structure and Klotho levels. |
| **92** | **Kuzmova (2021)[94]** | - A positive correlation between α-klotho levels and TBS was shown in the CKD population. | - Despite measuring vitamin D and PTH, confounding effects from these markers on TBS and bone density were not clearly analyzed. |
| **93** | **Lima (2019)[95]** | - No correlation between histomorphometric bone parameters and α-klotho levels was demonstrated. - α-Klotho levels did not correlate with bone turnover. | - Vitamin D and phosphate binder usage were not fully controlled in the analysis, which may impact the integrity of the results. |
| **94** | **Marchelek-Mysliwiec (2018)[96]** | - There were no significant differences in α-klotho levels between the CKD and control groups. - There was no correlation between α-klotho levels and BMD or other bone markers. - A negative correlation between lean mass and α-klotho levels was shown. | - The authors suggest that heterogeneity in α-klotho assays may explain the discrepancy with the literature. |
| **95** | **Martin-Gonzalez (2023)[97]** | - No significant difference in α-klotho levels was observed between systemic lupus erythematosus patients and controls. - Musculoskeletal manifestations in patients correlated with increased α-klotho levels. - The relationship between α-klotho levels and muscle atrophy was shown to be statistically significant. | - Small sample size for subgroups; only 3 patients with muscle atrophy were analyzed, reducing statistical power.   Only female participants were included, limiting generalizability to male patients. |
| **96** | **Matei (2022)[98]** | - α-Klotho negatively predicted lumbar spine BMD in kidney transplant patients. - α-Klotho was a significantly positive predictor of osteocalcin, a bone turnover marker. | In the control group, α-klotho and osteocalcin levels were missing, which may limit the comparative interpretation of their effects on bone metabolism​. |
| **97** | **Neves (2021)[99]** | - Dynamic RT significantly increased BMD in multiple regions such as the femoral bone, neck, and lumbar spine. - This improvement is shown to be associated with an increase in α-klotho levels. |  |
| **98** | **Pedersen (2020)[100]** | - Serum α-Klotho levels measured by the TRF immunoassay were roughly 1000-fold higher than those measured by the IBL ELISA. - No correlation was found between the two assays. | - The epitope specificity and source of recombinant Klotho differ between assays, making it unclear which Klotho isoforms are being detected. |
| **99** | **Ribeiro (2020)[101]** | - Lower α-klotho levels were independently associated with an increased risk of bone fractures. - Very low α-klotho levels (<268 pg/mL) were associated with a 2.95-fold increased fracture risk. | - The study did not include measurements of BMD, which may limit the understanding of the link between α-klotho and fracture risk. |
| **100** | **Salamanna (2024)[102]** | - α-Klotho levels were significantly lower in osteosarcopenic patients compared to both osteopenic and sarcopenic patients | - The study was a pilot with a limited number of patients, which reduces the statistical power and generalizability of findings.   This study is one of the first to identify its direct link to osteosarcopenia, suggesting it could be a potential biomarker or therapeutic target. |
| **101** | **Sawada (2024)[103]** | No significant associations were shown between α-klotho or FGF23 levels and BMD at the lumbar spine, femoral neck, or total hip​. | - A very old (80> years old) population was investigated in this study.   54.5% of participants had osteopenia, and 57% were vitamin D deficient. These conditions may have a confounding relationship with the α-klotho and BMD. |
| **102** | **Semba (2016)[104]** | - Participants in the highest tertile of α-klotho had higher knee extension strength at baseline compared to those in the lowest tertile; this association remained significant after adjusting for confounding factors. - Over four years of follow-up, participants with higher α-klotho levels had a slower decline in knee strength. - This study demonstrated that grip strength and knee strength were correlated. | - The study included only adults aged 71–80 years, limiting generalizability to younger populations. |
| **103** | **Teng Lee (2023)[121]** | - α-Klotho levels were not significantly associated with BMD in this patient cohort. - However, intact FGF23 (iFGF23) levels were significantly associated with lower BMD at both the femoral neck and lumbar spine in hemodialysis patients. | - BMD at the lumbar spine may be affected by vascular calcifications in hemodialysis patients, which may potentially overestimate the bone density. |
| **104** | **Wang (2024)[106]** | - α-Klotho levels were shown to have a significantly negative correlation with alveolar bone loss. - Higher α-klotho levels were also protective against severe periodontal attachment loss. | - This study uses data from the NHANES cohort.   The analysis did not include the potential confounding factors such as medications, which may affect bone metabolism. |
| **105** | **Wei (2019)[107]** | - Higher α-klotho levels were significantly associated with higher lumbar spine and femoral neck BMD, which implies a protective role of α-klotho in maintaining bone density. - α-Klotho levels were negatively correlated with PTH. |  |
| **106** | **Xie (2024)[108]** | - In multivariate analysis, a significant negative correlation between α-klotho levels and the risk of low muscle mass was demonstrated. Correlation was   particularly prominent among female participants.   - A positive correlation between α-klotho and appendicular lean mass was shown. | - This study uses data from the NHANES cohort. |
| **107** | **Yun (2020)[109]** | - In both univariable and multivariable regression analyses, no significant association between α-klotho levels and TBS was shown. - There was no correlation between α-Klotho levels and fracture incidence. - No significant correlation was observed between α-klotho and FGF23 levels, which may imply a disrupted FGF23–α-klotho axis in hemodialysis patients. | - Potential confounding factors that are not controlled, such as vitamin D levels and phosphate binder use, may have influenced the statistical integrity. |
| **108** | **Zhang (2023)[7]** | - α-Klotho levels were positively correlated with thoracic spine and trunk bone BMD. - At low α-klotho concentrations, a significant positive correlation was shown at all sites except lumbar spine BMD. Whereas, at high α-klotho levels, only thoracic spine and trunk BMD maintained a stable positive relationship with α-klotho. - A non-linear relationship between α-klotho and BMD was demonstrated. | - This study uses data from the NHANES cohort. - Vitamin D, PTH, and other key bone metabolism markers that may have potential confounding effects were not fully controlled for in the correlation analysis. |
| **109** | **Zheng (2018)[110]** | - Higher α-klotho levels were associated with higher BMD in both the femoral neck and lumbar spine. - α-Klotho levels were significantly lower in osteoporotic patients compared to those with normal BMD. - Patients with lumbar spinal osteopenia also had significantly lower α-klotho levels than those with normal BMD. | - In this study, potential confounding factors such as phosphate binders, vitamin D supplements, and calcimimetics are not controlled in the analysis. |

**Abbreviations:** LMI: Lean Mass Index, FMI: Fat Mass Index, BMI: Body Mass Index, BMD: Bone Mass Density, CKD: Chronic Kidney Disease, DXA: Dual-energy X-ray Absorptiometry, PTH: Parathyroid Hormone, FGF23: Fibroblast Growth Factor 23, TBS: Trabecular Bone Score, NHANES: National Health and Nutrition Examination Survey, InCHIANTI: Invecchiare in Chianti, ADL: Activity of Daily Living, MMSE: Mini-Mental State Examination, SPPB: Short Physical Performance Battery, 6MWT: 6-Minute Walk Test, STS: Sit-to-Stand Test, RT: Resistance Training, CSF: Cerebrospinal Fluid, MCI: Mild Cognitive Impairment, MoCa: Montreal Cognitive Assessment, AFT: Animal Fluency Test, CERAD: Consortium to Establish a Registry for Alzheimer’s Disease, DSST: Digit Symbol Substitution Test, NOx: nitrite/nitrate, SOD: superoxide dismutase, ADMA: asymmetric dimethylarginine.

**
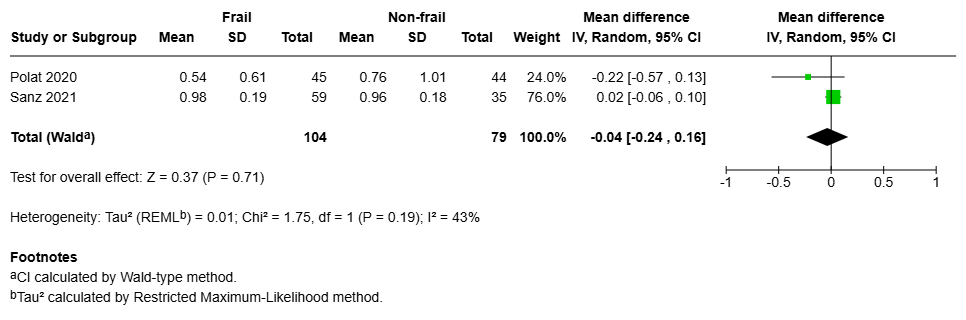
**

**Supplementary Figure S1.** Meta-analysis of the klotho and frailty relationship with studies reporting Klotho levels for frail and non-frail groups.

**A)
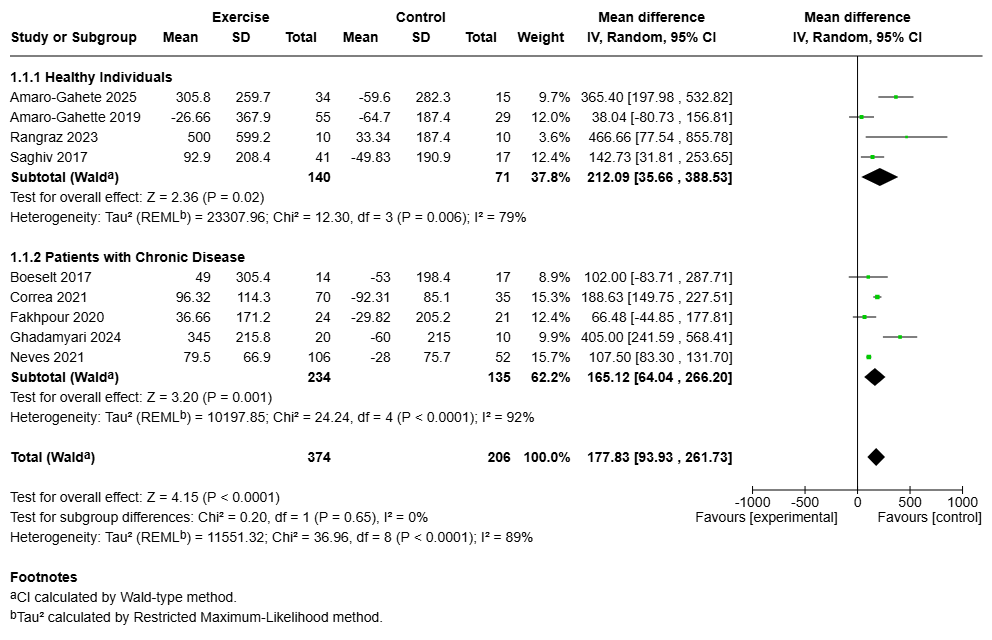
**

**B)
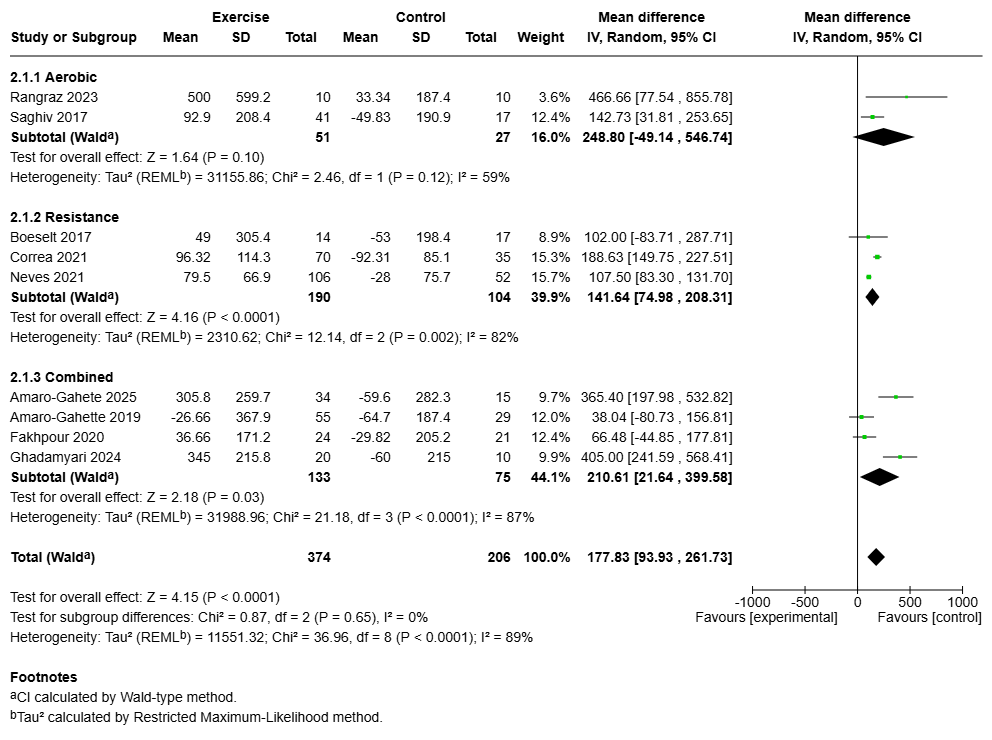
**

**Supplementary Figure S2.** Subgroup analyses of exercise effects on circulating α-klotho levels.

(A) Subgroup analysis by population (healthy vs. chronic disease).

(B) Subgroup analysis by exercise modality (aerobic, resistance, and combined training).


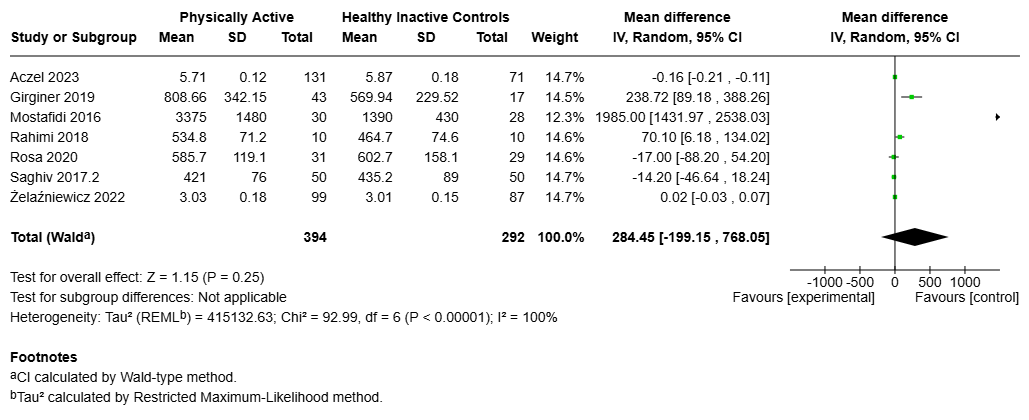
**Supplementary Figure S3.** Meta-analysis of the Association Between Habitual Physical Activity and Circulating α-Klotho Levels.


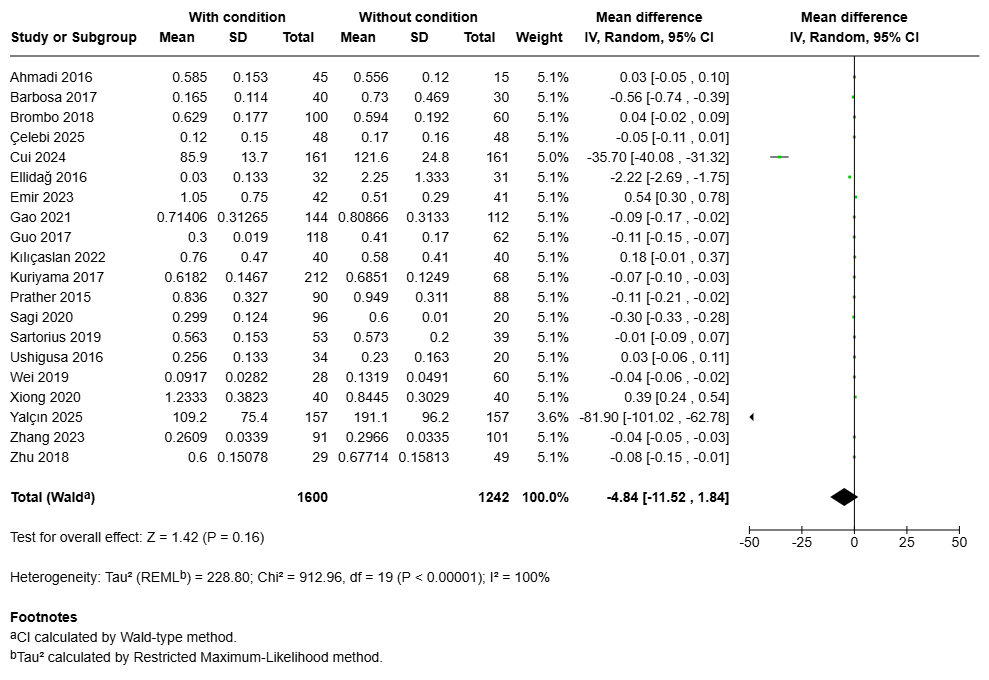


**Supplementary Figure S4.** Meta-analysis of Circulating α-Klotho Levels in Individuals With and Without Neuropsychiatric Conditions.


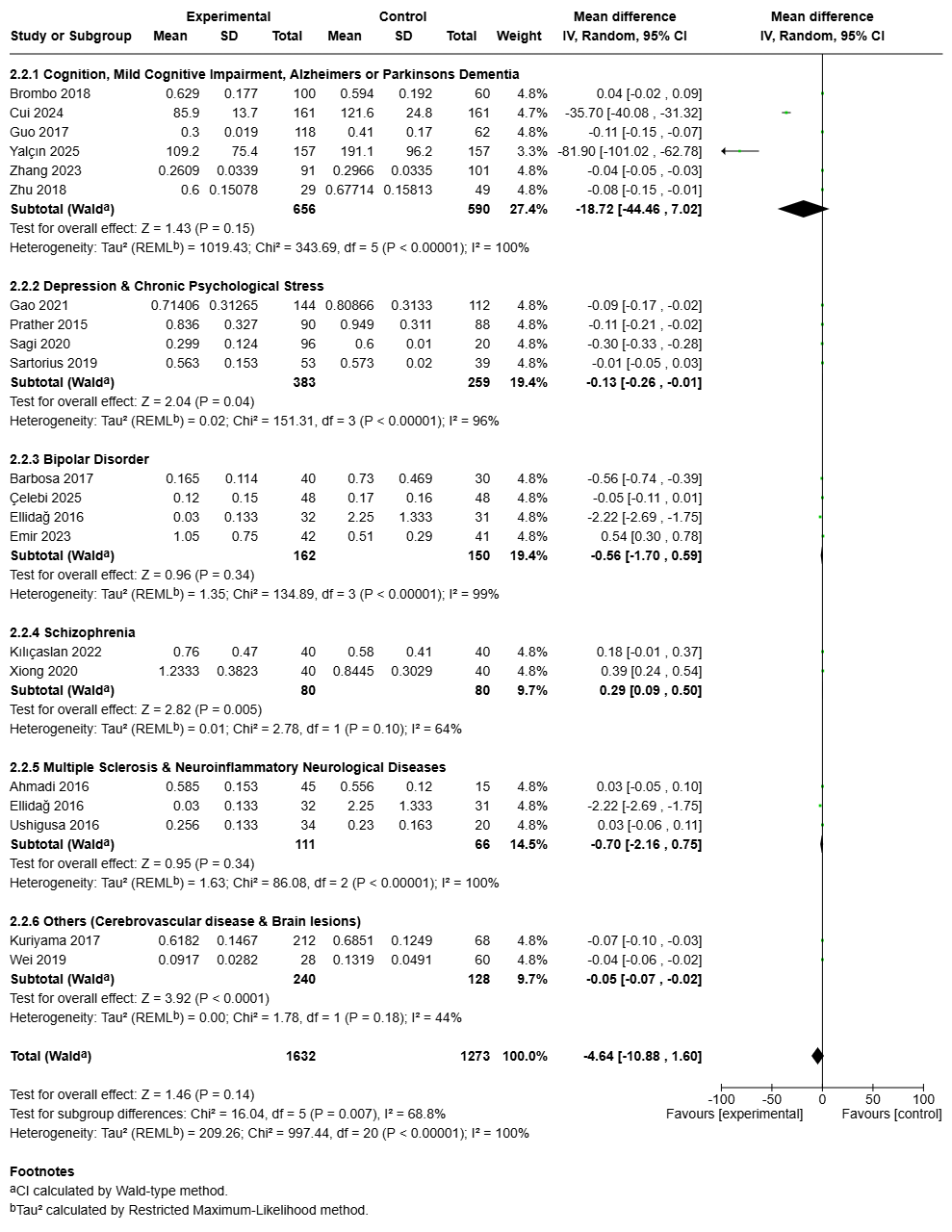


**Supplementary Figure S5.** Meta-analysis of the klotho level difference in patients with different subgroups of neuropsychiatric conditions (shown in detail in **Figure S6-S10**)

**
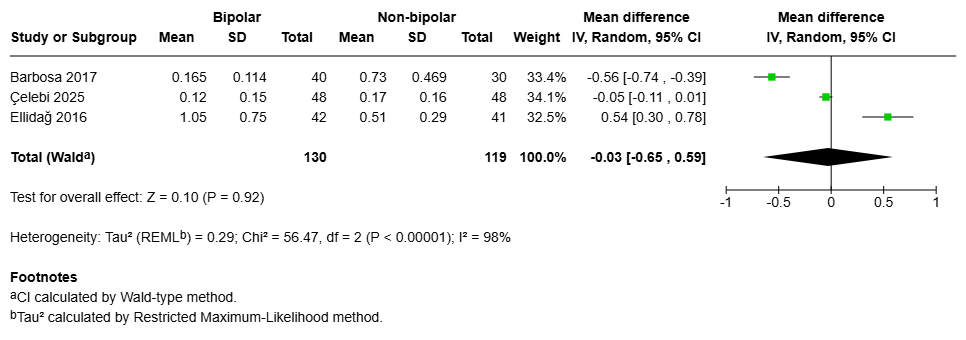
**

**Supplementary Figure S6.** Meta-analysis of the klotho level difference in patients with versus without bipolar disorder.

**
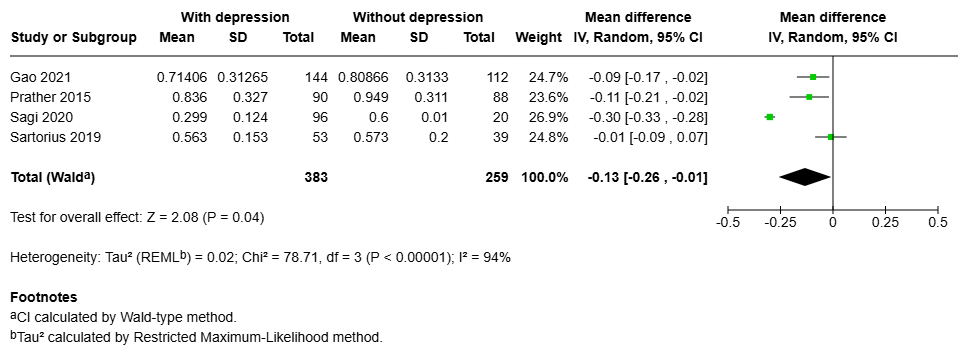
**

**Supplementary Figure S7.** Meta-analysis of the klotho level difference in patients with versus without depression.

**
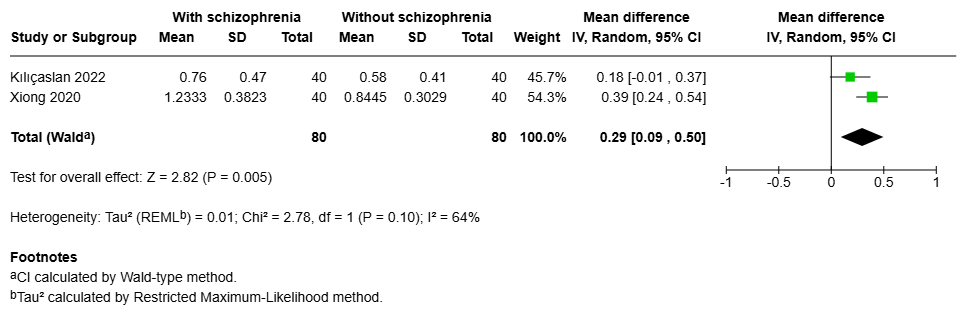
**

**Supplementary Figure S8.** Meta-analysis of the klotho level difference in patients with versus without schizophrenia.

**
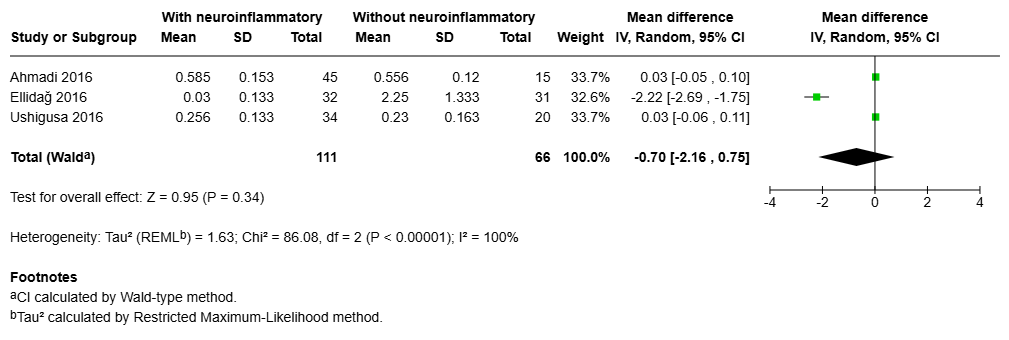
**

**Supplementary Figure S9.** Meta-analysis of the klotho level difference in patients with versus without neuroinflammatory conditions.

**
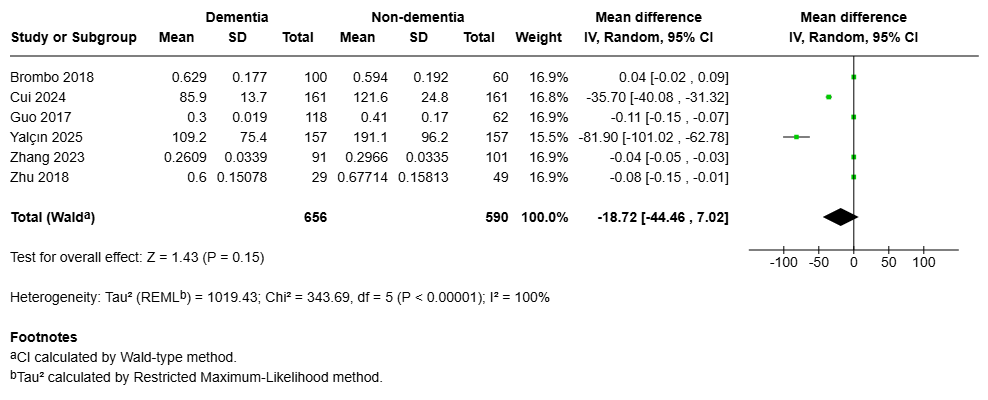
**

**Supplementary Figure S10.** Meta-analysis of the klotho level difference in patients with versus without dementia, mild cognitive impairment, Alzheimer’s dementia, and Parkinson’s disease dementia.

**
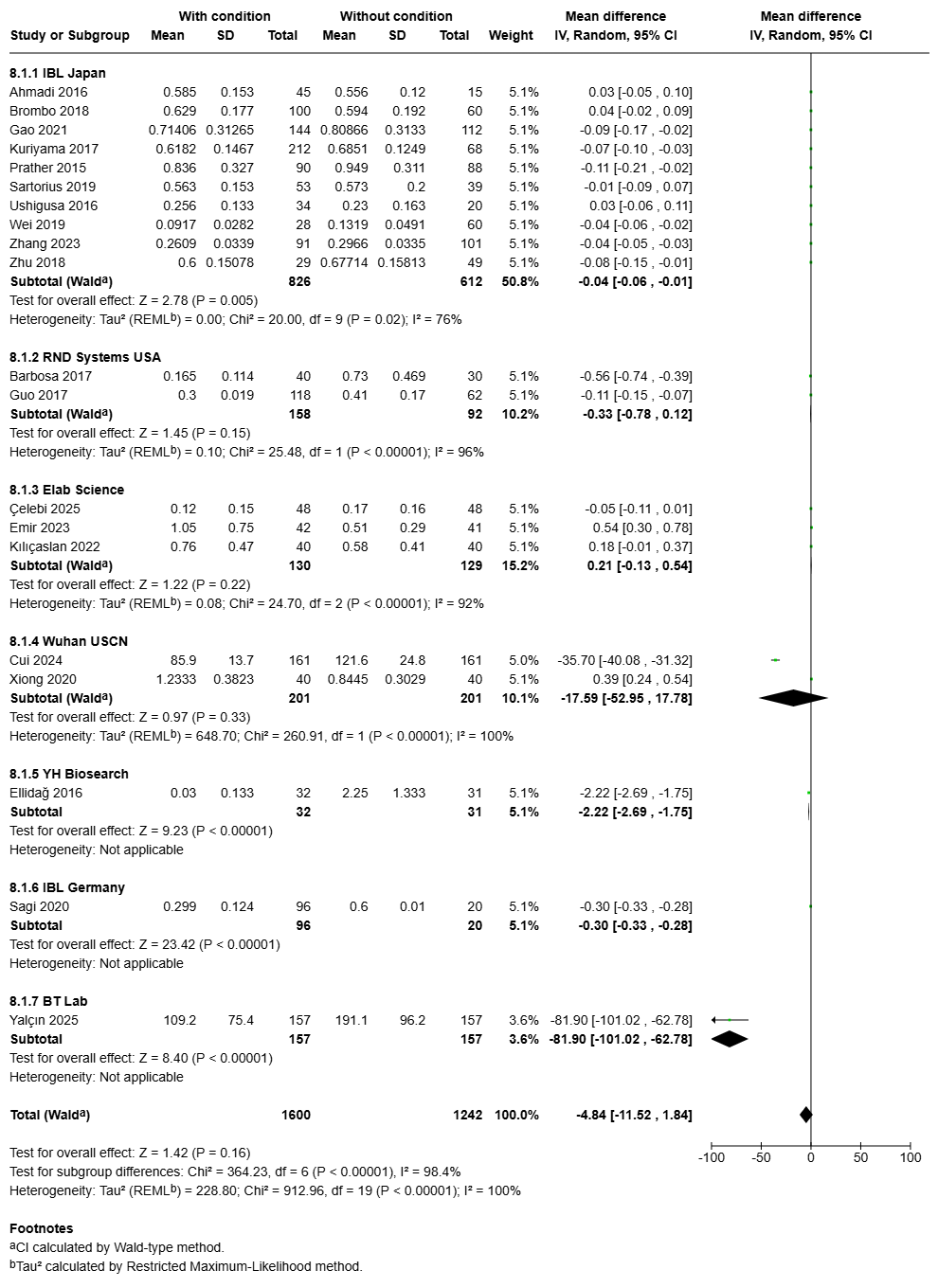
**

**Supplementary Figure S11.** Meta-analysis of the klotho level difference by the different subgroups classified by the type of ELISA assays.

1. **Osteopenia versus Non-osteopenia group**

**
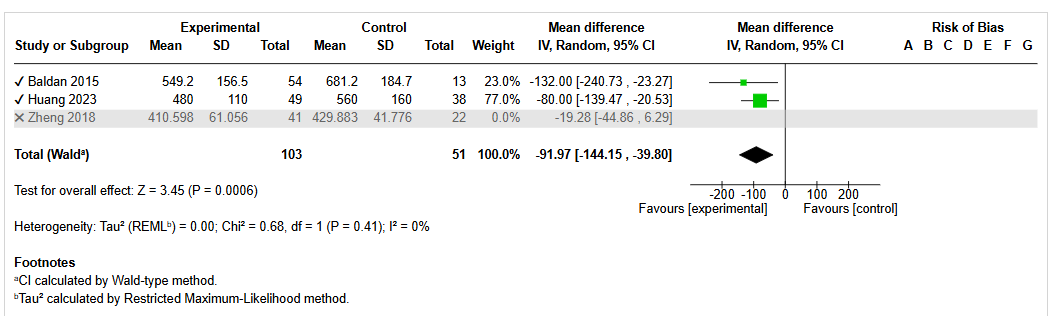
**

1. **Osteoporosis versus Non-osteoporosis Group**

**
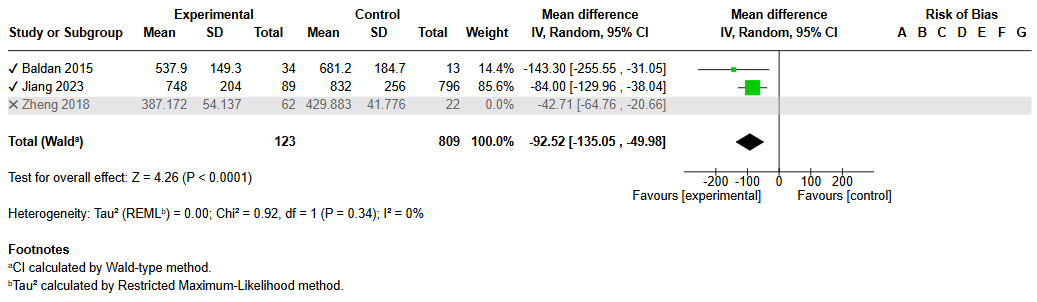
**

1. **Fractures versus Non-fractures Group
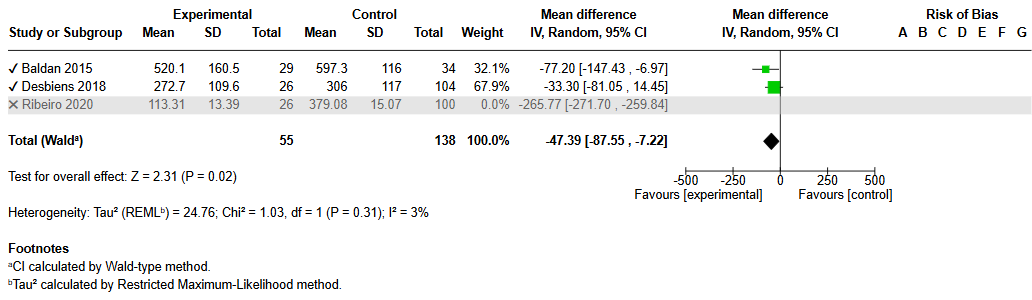
**
2. **Supplementary Figure S12.** Sensitivity analyses for osteopenia (A), osteoporosis (B), and fractures (C).

**REFERENCES**

1. Branco, P., et al., *Fibrosis of Peritoneal Membrane, Molecular Indicators of Aging and Frailty Unveil Vulnerable Patients in Long-Term Peritoneal Dialysis.* Int J Mol Sci, 2023. **24**(5).

2. Chuang, M.H., et al., *Association between soluble α-klotho and mortality risk in middle-aged and older adults.* Front Endocrinol (Lausanne), 2023. **14**: p. 1246590.

3. Guan, Z., L. Ma, and C. Wu, *Association between Serum Klotho and Physical Frailty in Middle-Aged and Older Adults: Finding From the National Health and Nutrition Examination Survey.* J Am Med Dir Assoc, 2023. **24**(8): p. 1173-1178.e2.

4. Jiang, Z., et al., *L-shaped association of serum α-Klotho and frailty among the middle-aged and older adults: results from NHANES 2007-2016.* BMC Geriatr, 2023. **23**(1): p. 716.

5. Koyama, D., et al., *Soluble αKlotho as a candidate for the biomarker of aging.* Biochem Biophys Res Commun, 2015. **467**(4): p. 1019-25.

6. Li, H., et al., *U-shaped association between serum Klotho and accelerated aging among the middle-aged and elderly US population: a cross-sectional study.* BMC Geriatr, 2023. **23**(1): p. 780.

7. Longo, P.L., et al., *Effects of physical distancing by COVID-19 pandemic on diet quality, neurological and immunological markers, and fecal microbiota of Brazilian older women.* Front Nutr, 2022. **9**: p. 972100.

8. Luo, H., et al., *Serum klotho levels and mortality patterns in frail individuals: unraveling the u-shaped association.* Aging Clin Exp Res, 2024. **36**(1): p. 92.

9. Polat, Y., et al., *The relationship between frailty and serum alpha klotho levels in geriatric patients.* Arch Gerontol Geriatr, 2020. **91**: p. 104225.

10. Sági, B., et al., *Pro- and anti-inflammatory factors, vascular stiffness and outcomes in chronic hemodialysis patients.* Physiol Int, 2020. **107**(2): p. 256-266.

11. Semba, R.D., et al., *Relationship of low plasma klotho with poor grip strength in older community-dwelling adults: the InCHIANTI study.* Eur J Appl Physiol, 2012. **112**(4): p. 1215-20.

12. Shardell, M., et al., *Plasma Klotho and Frailty in Older Adults: Findings From the InCHIANTI Study.* J Gerontol A Biol Sci Med Sci, 2019. **74**(7): p. 1052-1057.

13. Yuan, M., et al., *Linear-inverse associations of serum Klotho protein with prevalence of frailty among adults in the United States.* Am J Transl Res, 2024. **16**(7): p. 3090-3098.

14. Żelaźniewicz, A., J. Nowak-Kornicka, and B. Pawłowski, *S-Klotho level and physiological markers of cardiometabolic risk in healthy adult men.* Aging (Albany NY), 2022. **14**(2): p. 708-727.

15. Zhang, C., et al., *Association between Dietary Inflammatory Index and serum Klotho concentration among adults in the United States.* BMC Geriatr, 2022. **22**(1): p. 528.

16. Aczel, D., et al., *The Circulating Level of Klotho Is Not Dependent upon Physical Fitness and Age-Associated Methylation Increases at the Promoter Region of the Klotho Gene.* Genes (Basel), 2023. **14**(2).

17. Amaro-Gahete, F., et al., *Exercise training increases the S-Klotho plasma levels in sedentary middle-aged adults: A randomised controlled trial. The FIT-AGEING study.* Journal of sports sciences, 2019. **37**(19): p. 2175-2183.

18. Amaro-Gahete, F.J., et al., *Association of physical activity and fitness with S-Klotho plasma levels in middle-aged sedentary adults: The FIT-AGEING study.* Maturitas, 2019. **123**: p. 25-31.

19. Amaro-Gahete, F.J., et al., *Impact of 24-week supervised concurrent exercise on S-Klotho and vitamin D levels: A randomized controlled trial.* J Sports Sci, 2024. **42**(24): p. 2562-2571.

20. Arroyo, E., et al., *Relationship between klotho and physical function in healthy aging.* Sci Rep, 2023. **13**(1): p. 21158.

21. Boeselt, T., et al., *Benefits of High-Intensity Exercise Training to Patients with Chronic Obstructive Pulmonary Disease: A Controlled Study.* Respiration, 2017. **93**(5): p. 301-310.

22. Chen, S., et al., *Association between weight change and serum anti-aging protein α-Klotho: a cross-sectional study in middle-aged and older adults.* Sci Rep, 2024. **14**(1): p. 18624.

23. Collins, K.A., et al., *Change in circulating klotho in response to weight loss, with and without exercise, in adults with overweight or obesity.* Front Aging, 2023. **4**: p. 1213228.

24. Corrêa, H.L., et al., *Blood Flow Restriction Training Blunts Chronic Kidney Disease Progression in Humans.* Med Sci Sports Exerc, 2021. **53**(2): p. 249-257.

25. Crasto, C.L., et al., *Relationship of low-circulating "anti-aging" klotho hormone with disability in activities of daily living among older community-dwelling adults.* Rejuvenation Res, 2012. **15**(3): p. 295-301.

26. Ercan, Z., et al., *Effects of acute aerobic exercise on cytokines, klotho, irisin, and vascular endothelial growth factor responses in rheumatoid arthritis patients.* Ir J Med Sci, 2023. **192**(1): p. 491-497.

27. Fakhrpour, R., et al., *Effect of Sixteen Weeks Combined Training on FGF-23, Klotho, and Fetuin-A Levels in Patients on Maintenance Hemodialysis.* Iran J Kidney Dis, 2020. **14**(3): p. 212-218.

28. Ghadamyari, N., et al., *The effect of 8 weeks of endurance and resistance exercises on the serum levels of FGF23 and s-Klotho in type 2 diabetic women.* International Journal of Diabetes in Developing Countries, 2024: p. 1-8.

29. Girginer, F., et al., *Comparison of some plasma inflammation markers in elite Master athletes, recreational athletes and sedentary males.* Türkiye Klinikleri. Tip Bilimleri Dergisi, 2019. **39**(2): p. 202-211.

30. Iturriaga, T., et al., *Acute Impacts of Different Types of Exercise on Circulating α-Klotho Protein Levels.* Front Physiol, 2021. **12**: p. 716473.

31. Matsubara, T., et al., *Aerobic exercise training increases plasma Klotho levels and reduces arterial stiffness in postmenopausal women.* Am J Physiol Heart Circ Physiol, 2014. **306**(3): p. H348-55.

32. Middelbeek, R.J.W., et al., *Exercise intensity regulates cytokine and klotho responses in men.* Nutr Diabetes, 2021. **11**(1): p. 5.

33. Morishima, T. and E. Ochi, *Impact of a single bout of resistance exercise on serum Klotho in healthy young men.* Physiol Rep, 2021. **9**(21): p. e15087.

34. Mostafidi, E., et al., *Serum Klotho Levels in Trained Athletes.* Nephrourol Mon, 2016. **8**(1): p. e30245.

35. Rahimi, S., K. Khademvatani, and M.R. Zolfaghari, *Association of circular Klotho and insulin-like growth factor 1 with cardiac hypertrophy indexes in athlete and non-athlete women following acute and chronic exercise.* Biochem Biophys Res Commun, 2018. **505**(2): p. 448-452.

36. Rangraz, M.H.A. and S. Hojjat, *The Effects of a Six-Week Aerobic Exercise on Serum Levels of Klotho, IL-10, IL-1, and Oxidative Stress in Sedentary Young Women.* Crescent Journal of Medical & Biological Sciences, 2023. **10**(2).

37. Saghiv, M., E. Goldhammer, and E. Radzishevski, *The impact of 12 weeks exercise training on circulating soluble-klotho and Pro-BNP in coronary artery disease patients.* J Cardiol Vasc Res, 2017. **1**: p. 1-4.

38. Saghiv, M., et al., *Long lasting chronic resistive training effects on circulating S-Klotho and IGF-1.* Archives of Clinical and Biomedical Research, 2017. **1**(2): p. 69-75.

39. Rosa, T.S., et al., *Sprint and endurance training in relation to redox balance, inflammatory status and biomarkers of aging in master athletes.* Nitric Oxide, 2020. **102**: p. 42-51.

40. Shardell, M., et al., *Serum 25-Hydroxyvitamin D, Plasma Klotho, and Lower-Extremity Physical Performance Among Older Adults: Findings From the InCHIANTI Study.* J Gerontol A Biol Sci Med Sci, 2015. **70**(9): p. 1156-62.

41. Shardell, M., et al., *Plasma Soluble αKlotho, Serum Fibroblast Growth Factor 23, and Mobility Disability in Community-Dwelling Older Adults.* J Endocr Soc, 2020. **4**(5): p. bvz032.

42. Snigdha, A., et al., *Yoga-based lifestyle intervention for healthy ageing in older adults: a two-armed, waitlist randomized controlled trial with multiple primary outcomes.* Geroscience, 2024. **46**(6): p. 6039-6054.

43. Tan, S.J., et al., *High-intensity physical exercise increases serum α-klotho levels in healthy volunteers.* J Circ Biomark, 2018. **7**: p. 1849454418794582.

44. Tetik Dündar, S., M. Kuzucu, and S. Varol, *Effect of the aerobic power test performed at low-medium altitude on the myostatin, pgc-1 alpha and klotho levels.* Gazzetta Medica Italiana Archivio per le Scienze Mediche, 2023. **182**(2).

45. Valenzuela, P.L., et al., *Physical performance, plasma S-klotho, and all-cause mortality in elderly dialysis patients: A prospective cohort study.* Exp Gerontol, 2019. **122**: p. 123-128.

46. Ahmadi, M., et al., *Multiple sclerosis influences on the augmentation of serum Klotho concentration.* J Neurol Sci, 2016. **362**: p. 69-72.

47. Barbosa, I.G., et al., *Klotho dysfunction: A pathway linking the aging process to bipolar disorder?* J Psychiatr Res, 2017. **95**: p. 80-83.

48. Brombo, G., et al., *Lower Plasma Klotho Concentrations Are Associated with Vascular Dementia but Not Late-Onset Alzheimer's Disease.* Gerontology, 2018. **64**(5): p. 414-421.

49. Cui, L., et al., *Analysis of the relationship between mild cognitive impairment and serum klotho protein and insulin-like growth factor-1 in the elderly.* Technol Health Care, 2024. **32**(3): p. 1455-1462.

50. Çelebi, Z., et al., *Relationship between klotho, neurotrophic factors (BDNF, NGF, GDNF) and cognitive functions in patients with bipolar disorder.* BMC Psychiatry, 2025. **25**(1): p. 53.

51. Ellidag, H.Y., et al., *The Three Sisters of Fate in Multiple Sclerosis: Klotho (Clotho), Fibroblast Growth Factor-23 (Lachesis), and Vitamin D (Atropos).* Ann Neurosci, 2016. **23**(3): p. 155-161.

52. Sirlier Emir, B., et al., *The roles of Klotho and FGF-23 in bipolar manic episode.* Eur Rev Med Pharmacol Sci, 2023. **27**(5 Suppl): p. 101-108.

53. Gaitán, J.M., et al., *Effects of Aerobic Exercise Training on Systemic Biomarkers and Cognition in Late Middle-Aged Adults at Risk for Alzheimer's Disease.* Front Endocrinol (Lausanne), 2021. **12**: p. 660181.

54. Gao, J., et al., *Association of triiodothyronine levels with left ventricular function, cardiovascular events, and mortality in hemodialysis patients.* Int J Artif Organs, 2017. **40**(2): p. 60-66.

55. Ge, S., et al., *Serum soluble alpha-klotho klotho and cognitive functioning in older adults aged 60 and 79: an analysis of cross-sectional data of the National Health and Nutrition Examination Survey 2011 to 2014.* BMC Geriatr, 2024. **24**(1): p. 245.

56. Guo, Y., et al., *Serum Klotho, vitamin D, and homocysteine in combination predict the outcomes of Chinese patients with multiple system atrophy.* CNS Neurosci Ther, 2017. **23**(8): p. 657-666.

57. Kato, K., et al., *Association between serum magnesium levels and cognitive function in patients undergoing hemodialysis.* Clin Exp Nephrol, 2024. **28**(11): p. 1187-1196.

58. Kazgan Kılıçaslan, A., et al., *Serum Klotho and FGF23 Levels in Patients with Schizophrenia.* Psychiatry Clin Psychopharmacol, 2022. **32**(3): p. 229-236.

59. Kundu, P., et al., *Serum Levels of α-Klotho Are Correlated with Cerebrospinal Fluid Levels and Predict Measures of Cognitive Function.* J Alzheimers Dis, 2022. **86**(3): p. 1471-1481.

60. Kuriyama, N., et al., *Association between α-Klotho and Deep White Matter Lesions in the Brain: A Pilot Case Control Study Using Brain MRI.* J Alzheimers Dis, 2018. **61**(1): p. 145-155.

61. Linghui, D., et al., *The relationship between serum klotho and cognitive performance in a nationally representative sample of US adults.* Front Aging Neurosci, 2023. **15**: p. 1053390.

62. Prather, A.A., et al., *Longevity factor klotho and chronic psychological stress.* Transl Psychiatry, 2015. **5**(6): p. e585.

63. Sanz, B., et al., *Low serum klotho concentration is associated with worse cognition, psychological components of frailty, dependence, and falls in nursing home residents.* Sci Rep, 2021. **11**(1): p. 9098.

64. Sartorius, A., et al., *Peripheral levels of the anti-aging hormone Klotho in patients with depression.* J Neural Transm (Vienna), 2019. **126**(6): p. 771-776.

65. Shardell, M., et al., *Plasma Klotho and Cognitive Decline in Older Adults: Findings From the InCHIANTI Study.* J Gerontol A Biol Sci Med Sci, 2016. **71**(5): p. 677-82.

66. Ushigusa, T., et al., *Soluble α-klotho is a potential biomarker associated with neuropsychiatric systemic lupus erythematosus.* Clin Immunol, 2016. **165**: p. 29-34.

67. Vázquez‐Lorente, H., et al., *Physical exercise improves memory in sedentary middle‐aged adults: Are these exercise‐induced benefits associated with S‐Klotho and 1, 25‐dihydroxivitamin D? The FIT‐AGEING randomized controlled trial.* Scandinavian Journal of Medicine & Science in Sports, 2024. **34**(1): p. e14519.

68. Wu, Y., et al., *Relationship of Klotho with cognition and dementia: Results from the NHANES 2011-2014 and Mendelian randomization study.* Transl Psychiatry, 2023. **13**(1): p. 337.

69. Wu, F., et al., *Association of serum klotho with cognitive function among individuals with nonalcoholic fatty liver disease.* Front Aging Neurosci, 2024. **16**: p. 1487182.

70. Xiong, J.W., et al., *Increased Plasma Level of Longevity Protein Klotho as a Potential Indicator of Cognitive Function Preservation in Patients With Schizophrenia.* Front Neurosci, 2020. **14**: p. 610.

71. Yalcin, A., et al., *Serum alpha klotho levels in Parkinson's Disease.* Neurol Sci, 2025. **46**(2): p. 743-749.

72. Yokoyama, J.S., et al., *Systemic klotho is associated with KLOTHO variation and predicts intrinsic cortical connectivity in healthy human aging.* Brain Imaging Behav, 2017. **11**(2): p. 391-400.

73. Zhang, J. and A. Zhang, *Relationships between serum Klotho concentrations and cognitive performance among older chronic kidney disease patients with albuminuria in NHANES 2011-2014.* Front Endocrinol (Lausanne), 2023. **14**: p. 1215977.

74. Zhang, H., L. Yu, and G. Yun, *Reduced Serum Levels of Klotho are Associated with Mild Cognitive Impairment in Patients with Type 2 Diabetes Mellitus.* Diabetes Metab Syndr Obes, 2023. **16**: p. 129-137.

75. Zhu, B., et al., *Differential expression of serum biomarkers in hemodialysis patients with mild cognitive decline: A prospective single-center cohort study.* Sci Rep, 2018. **8**(1): p. 12250.

76. Amaro-Gahete, F.J., et al., *Body composition and S-Klotho plasma levels in middle-aged adults: A cross-sectional study.* Rejuvenation research, 2019. **22**(6): p. 478-483.

77. Baldan, A., et al., *Klotho, a new marker for osteoporosis and muscle strength in β-thalassemia major.* Blood Cells Mol Dis, 2015. **55**(4): p. 396-401.

78. Chalhoub, D., et al., *Association of Serum Klotho with Loss of Bone Mineral Density and Fracture Risk in Older Adults.* J Am Geriatr Soc, 2016. **64**(12): p. e304-e308.

79. Desbiens, L.C., et al., *FGF23-klotho axis, bone fractures, and arterial stiffness in dialysis: a case-control study.* Osteoporos Int, 2018. **29**(10): p. 2345-2353.

80. Desbiens, L.C., et al., *FGF23-Klotho Axis and Fractures in Patients Without and With Early CKD: A Case-Cohort Analysis of CARTaGENE.* J Clin Endocrinol Metab, 2022. **107**(6): p. e2502-e2512.

81. Ferreira, A.C., et al., *Bone densitometry versus bone histomorphometry in renal transplanted patients: a cross-sectional study.* Transpl Int, 2021. **34**(6): p. 1065-1073.

82. Ferreira, A.C., et al., *The Role of Bone Volume, FGF23 and Sclerostin in Calcifications and Mortality; a Cohort Study in CKD Stage 5 Patients.* Calcif Tissue Int, 2022. **110**(2): p. 215-224.

83. Fukasawa, H., et al., *Plasma levels of fibroblast growth factor-23 are associated with muscle mass in haemodialysis patients.* Nephrology (Carlton), 2014. **19**(12): p. 784-90.

84. Gözükara, B., *Geriatrik populasyonda sarkopeni ve alfa klotho proteini ilişkisi*. 2019, Ankara Universitesi (Turkey).

85. Han, W., et al., *The relationship between serum fibroblast growth factor 23, Klotho, and lumbar spine bone mineral density in northern Chinese postmenopausal women.* Menopause, 2019. **26**(5): p. 546-553.

86. Huang, T., et al., *The Relationship Between Serum Fibroblast Growth Factor 23 and Klotho Protein and Low Bone Mineral Density in Middle-Aged and Elderly Patients with End-Stage Renal Disease.* Horm Metab Res, 2024. **56**(2): p. 142-149.

87. Hughes-Austin, J.M., et al., *Biomarkers of Bone Turnover Identify Subsets of Chronic Kidney Disease Patients at Higher Risk for Fracture.* J Clin Endocrinol Metab, 2020. **105**(8): p. e2903-11.

88. Jiang, J., et al., *Klotho reduces the risk of osteoporosis in postmenopausal women: a cross-sectional study of the National Health and Nutrition Examination Survey (NHANES).* BMC Endocr Disord, 2023. **23**(1): p. 151.

89. Jiang, K., et al., *The Analysis of Serum Klotho Protein Level and Related Gene Polymorphism in Osteoporotic Fracture of Elderly Patients with Osteoporosis.* Altern Ther Health Med, 2024. **30**(9): p. 229-233.

90. Katsaounis, P.V., et al., *Bone disease in patients with cirrhosis of different etiology and severity; are Klotho protein and osteoprotegerin potential biomarkers?* Scand J Gastroenterol, 2023. **58**(2): p. 185-192.

91. Kureya, Y., et al., *Down-Regulation of Soluble α-Klotho is Associated with Reduction in Serum Irisin Levels in Chronic Obstructive Pulmonary Disease.* Lung, 2016. **194**(3): p. 345-51.

92. Kužmová, Z., et al., *Fibroblast Growth Factor 23 and Klotho Are Associated With Trabecular Bone Score but Not Bone Mineral Density in the Early Stages of Chronic Kidney Disease: Results of the Cross-Sectional Study.* Physiol Res, 2021. **70**(Suppl 1): p. S43-s51.

93. Lima, F., et al., *Serum bone markers in ROD patients across the spectrum of decreases in GFR: Activin A increases before all other markers*Clin Nephrol, 2019. **91**(4): p. 222-230.

94. Marchelek-Mysliwiec, M., et al., *Association Between Plasma Concentration of Klotho Protein, Osteocalcin, Leptin, Adiponectin, and Bone Mineral Density in Patients with Chronic Kidney Disease.* Horm Metab Res, 2018. **50**(11): p. 816-821.

95. Martín-González, C., et al., *Alpha-Klotho protein in systemic lupus erythematosus.* Clin Exp Rheumatol, 2023. **41**(1): p. 41-47.

96. Matei, A., et al., *Body composition, adipokines, FGF23-Klotho and bone in kidney transplantation: Is there a link?* J Nephrol, 2022. **35**(1): p. 293-304.

97. Neves, R.V.P., et al., *Dynamic not isometric training blunts osteo-renal disease and improves the sclerostin/FGF23/Klotho axis in maintenance hemodialysis patients: a randomized clinical trial.* J Appl Physiol (1985), 2021. **130**(2): p. 508-516.

98. Pedersen, L., et al., *Soluble serum Klotho levels in healthy subjects. Comparison of two different immunoassays.* Clin Biochem, 2013. **46**(12): p. 1079-1083.

99. Ribeiro, A.L., et al., *FGF23-klotho axis as predictive factors of fractures in type 2 diabetics with early chronic kidney disease.* J Diabetes Complications, 2020. **34**(1): p. 107476.

100. Salamanna, F., et al., *A Pilot Study on Circulating, Cellular, and Tissue Biomarkers in Osteosarcopenic Patients.* Int J Mol Sci, 2024. **25**(11).

101. Sawada, K.M., et al., *Blood concentrations of α-Klotho and FGF-23 exhibit no correlation with bone mineral density in elderly individuals.* Einstein (Sao Paulo), 2024. **22**: p. eAO0412.

102. Semba, R.D., et al., *Low Plasma Klotho Concentrations and Decline of Knee Strength in Older Adults.* J Gerontol A Biol Sci Med Sci, 2016. **71**(1): p. 103-8.

103. Lee, W.-T., et al., *Serum intact fibroblast growth factor 23 levels are negatively associated with bone mineral density in chronic hemodialysis patients.* Journal of Clinical Medicine, 2023. **12**(4): p. 1550.

104. Wang, Z., et al., *Deciphering the Biological Aging Impact on Alveolar Bone Loss: Insights From α-Klotho and Renal Function Dynamics.* J Gerontol A Biol Sci Med Sci, 2024. **79**(9).

105. Wei, H., et al., *Serum klotho: a potential predictor of cerebrovascular disease in hemodialysis patients.* BMC Nephrol, 2019. **20**(1): p. 63.

106. Xie, J., et al., *Correlation analysis between left ventricular global longitudinal strain and major adverse cardiovascular event occurrence in patients with end-stage renal disease.* Int Urol Nephrol, 2024. **56**(9): p. 3031-3037.

107. Yun, H.J., et al., *Trabecular bone score may indicate chronic kidney disease-mineral and bone disorder (CKD-MBD) phenotypes in hemodialysis patients: a prospective observational study.* BMC Nephrol, 2020. **21**(1): p. 299.

108. Zhang, Y., et al., *Association between serum soluble α-klotho and bone mineral density (BMD) in middle-aged and older adults in the United States: a population-based cross-sectional study.* Aging Clin Exp Res, 2023. **35**(10): p. 2039-2049.

109. Zheng, S., et al., *Correlation of serum levels of fibroblast growth factor 23 and Klotho protein levels with bone mineral density in maintenance hemodialysis patients.* Eur J Med Res, 2018. **23**(1): p. 18.
